# Novel amides of mycophenolic acid and some heterocyclic derivatives as immunosuppressive agents

**DOI:** 10.1080/14756366.2022.2127701

**Published:** 2022-10-03

**Authors:** Juliusz Maksymilian Walczak, Dorota Iwaszkiewicz-Grześ, Michalina Ziomkowska, Magdalena Śliwka-Kaszyńska, Mateusz Daśko, Piotr Trzonkowski, Grzegorz Cholewiński

**Affiliations:** a Department of Organic Chemistry, Faculty of Chemistry, Gdańsk University of Technology, Gdańsk, Poland; b Department of Medical Immunology, Faculty of Medicine, Medical University of Gdańsk, Gdańsk, Poland; c Perlan Technologies sp. z o.o, Warszawa, Poland; d Department of Inorganic Chemistry, Faculty of Chemistry, Gdańsk University of Technology, Gdańsk, Poland

**Keywords:** Mycophenolic acid, amide derivatives, heterocycles, benzoxazole, IMPDH inhibition

## Abstract

The group of 18 new amide derivatives of mycophenolic acid (MPA) and selected heterocyclic amines was synthesised as potential immunosuppressive agents functioning as inosine-5′-monophosphate dehydrogenase (IMPDH) uncompetitive inhibitors. The synthesis of 14 of them employed uronium-type activating system (TBTU/HOBt/DIPEA) while 4 of them concerned phosphonic acid anhydride method (T3P/Py) facilitating amides to be obtained in moderate to excellent yields without the need of phenolic group protection. Most of optimised protocols did not require complicated reaction work-ups, including chromatographic, solvent-consuming methods. The biological activity assay was performed on the T-Jurkat cell line and peripheral mononuclear blood cells (PBMCs) which are both dedicated for antiproliferative activity determination. Each of designed derivatives was characterised by reduced cytotoxicity and benzoxazole analogue (**A2**) revealed the most promising activity. Subsequently, an observed structure-activity relationship was discussed.

## Graphical Abstract

**Figure:**
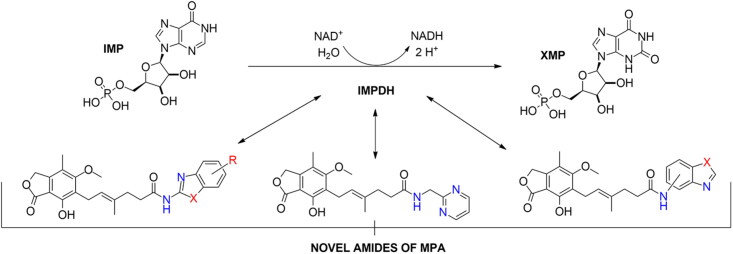

## Introduction

Inosine-5′-monophosphate dehydrogenase (IMPDH) is an enzyme responsible for oxidation of inosine-5′-monophosphate into xanthine-5′-monophosphate (XMP) exploiting water molecule as well as NAD^+^ redox potential. This biotransformation is crucial for proper cell growth due to the fact that it triggers guanyl nucleotides formation which serve as RNA and DNA precursors, the energy reservoir for translation process, the cofactor for G-proteins as well as glycosylation precursor. Depletion of guanine-based nucleotides, which is induced by redirecting biosynthesis into adenine-based nucleotides pathway, results in impairment of *de novo* and salvage route of purine and pyrimidine nucleotides biosynthesis. This misregulation proceeds from phosphoribosyl pyrophosphate synthetase (PRPP) and ribonucleotides reductase (RNR) stimulation dependence accruing from guanine and adenine nucleotides. When the previous one activates enzymes’ bioactivities, the latter one inhibits them. Eventually, enhancement of adenosine nucleotides pool effects in malfunctioning of rapidly proliferating human cells^1^.

Mycophenolic acid (MPA) is one of the uncompetitive and reversible inhibitors of IMPDH. It means that MPA and IMP bind to an enzyme simultaneously thus blocking further biosynthesis of guanine-based nucleotides causing significant effects on the cell’s biochemistry^1–3^. Thereupon, MPA was previously found to inhibit human lymphocytes G and T responses to mitogenic stimulation thus resulting in immunosuppressive activity observation. This feature led to new immunosuppressants establishment, sodium mycophenolate (MMS) and mycophenolate mofetil (MMF) namely^4^. Due to their antiproliferative properties, they have been put to use in acute graft rejection prophylaxis and psoriasis. Despite severe side-effects (e.g. secondary microbial infections, red blood cells activity) arising from MPA usage, it is still in use for its unique risk-reducing feature of graft loss and acute rejection outperforming other immunosuppressants, e.g. Azathioprine^1^^,^^5^^,^^6^. It is worth mentioning that some attempts towards MPA-based bioactive compounds isolation from natural resources are still being made thus showing not only synthetically approachable derivatives. Some of them originate from marine-derived fungus *Penicillium sp.* SCSIO sof101 and *Rhizopsus oryzae* which are a reservoir for penicacids that exhibit from moderate to weak antibacterial properties^7^^,^^8^. Targeting of IMPDH by the MPA molecule may lead to anticancer properties exhibition *via* other molecular mechanisms. Enzyme’s inhibition can lead to a decrease in the expression of several antiapoptotic proteins and the deactivation of caspases which is an excellent starting point in osteosarcoma chemotherapy and the antimetastatic drugs designing in general^9^, anticancer potential of MPA is still under investigation^10^^,^^11^.

Bioactivity of MPA is preserved due to the selected functionalities present within its structure. Lactone and aromatic moiety, free phenolic group,/*E*/-shaped double bond, and carboxylic acid moiety are required to maintain compound’s activity thus making radical structural modifications forbidden^1^^,^^4^^,^^12^. Nevertheless, some amide-type derivatives were synthesised and positively biologically evaluated. Anilide-type derivatives of MPA have shown good bioactivity towards human and protozoal IMPDH making them potential immunosuppressants and antiprotozoal agents^13^. Hydroxamic acid and aminated heterocyclic derivers of MPA are prone to inhibit IMPDH as well as histone deacetylase (HDAC) rendering them for being anticancer agents for influencing some of the expression level of anticancer genes in abnormal cells, beside of its antiproliferative properties^14^^,^^15^. Another example of a unique amide-like derivative of MPA is connected with MPA-quinic acid conjugate. It bears subtly smaller activity towards IMPDH and protective effect against apoptosis at once. It also improved insulin release profile in INS-1E cell lines thus showing how such a bioactive conjugate would shift cytotoxicity at all^16^. Mycophenolic adenine dinucleotide (MAD) analogues were rationalised as another inclined IMPDH inhibitors^17^. MPA and biogenic amino acids showed moderate antibacterial properties, especially towards *S. aureus* and *K. pneumoniae*, most likely due to the inhibition of bacterial IMPDH^18^. Benzyl-derived amides of MPA are thought to be potent anticancer drugs candidates due to similar activity towards IMPDH as MPA and unique inhibitory properties in relation to breast, prostate, and glioblastoma cell lines^19^. Additionally, some amide derivers of MPA, especially one bearing long aliphatic spacer and terminal imidazole structural motif, tend to impede *Toxoplasma gondii* growth better than referential MPA molecule, both in *in-vitro* and *in-vivo* conditions, thus presenting excellent anti-protozoal properties and making it a potential drug candidate^20^.

Noteworthy, IMPDH inhibition concerns wide class of compounds, e.g. structures possessing urea moieties or various heterocyclic units, which have been investigated towards immunosuppressive, anticancer, antibacterial, and antiviral activities^1^^,^^21–23^.

Heterocyclic motifs are one of the most important constitutional patterns among all existing pharmaceuticals’ structures. In 2014, four of five most common prescribed drugs in the US contained heterocyclic fragments^24^. Of 59% of small-molecule remedies sold in the US, in the same year, also carried heterocyclic designs, but this time it belonged to the nitrogen-based heterocyclic compounds family^25^. These facts render heterocycles as being the most privileged and significant molecular architectures in the drug designing area. Heterocyclic structural units are known for their remarkable physicochemical properties. They may serve as versatile bioisosteres as they can be tuned in their lipophilicity, polarity and water-solubility upon currently desirable properties in molecular targets reaching. These features may be manipulated in relation to their aromaticity and polar properties developed by the presence of strong electronegative atoms^24^. Referring to previously mentioned examples, heterocyclic structural motifs such as 1,2,4-triazole, incorporated into MPA molecule, have shown similar activity towards IMPDH as MPA within itself^15^. Inhibitory properties of benzoxazole-based derivatives towards parasitical IMPDH have also been proved, making them potential antiparasitic agents^26^. Heterocyclic compounds are also known for their anti-inflammatory^27^, anticancer^28^^,^^29^, antimalarial^30^, and fungicidal properties^31^ and these parameters may be a source of added value beside antiproliferative nature of MPA.

In this work, we submitted molecular docking assay depicting binding force in substrate-enzyme complex between novel amide derivatives of MPA and IMPDH, then the synthesis of novel MPA derivatives and their structural characterisation and eventually biological evaluation involving T-Jurkat cell lines as well as peripheral mononuclear blood cells (PMBCs). MPA core was chosen upon previous literature premises^1–4^ and the significant role it plays in graft rejection prophylaxis, and more precisely, the significant cytotoxicity it shows to healthy cells^5^. Aforementioned examples of biological utility of amide-like derivatives of MPA^13^^–^^20^ prompted us to explore another class of amide derivers with heterocyclic structural motifs incorporated. The latter pattern utilisation was justified by its unique physicochemical properties, common application in small-molecule drugs^24^^,^^25^ and plenty of bioactivities^15^^,^^26–33^ which may serve as an added value to the referential MPA molecule. All of these elements brought together may represent attractive novel antiproliferative compounds in medicinal chemistry by lowering cytotoxic effects on healthy cells and maintaining approximate antiproliferative properties in relation to MPA-based drugs (MMS, MMF, or MPA within itself) at the same time.

## Results and discussion

### Molecular docking

To verify that compounds **A1–A18** are able to effectively bind to the IMPDH enzyme’s active site, molecular docking studies were performed. The X-ray structure of IMPDH enzyme was retrieved from the Protein Data Bank (Protein Data Bank accession code 1JR1) and properly prepared for docking calculations. The docking procedure of the optimised ligands was performed using AutoDock Vina version 1.1.2 software (Molecular Graphics Laboratory, The Scripps Research Institute, LaJolla, CA). The calculated results for the proposed structures of compounds A1–A18 indicated that they could, at least theoretically, efficiently bind to the active site of IMPDH protein. The free energies of binding of examined compounds were at the satisfactory level in the narrow range of −8.7 to −9.5 kcal·mol^−1^ (Table 1) and were noticeably lower than the free energy of binding of MPA (−7.8 kcal·mol^−1^) used as reference. The most favourable free energies of binding were determined for compounds A4 and A6 (−9.5 kcal·mol^−1^ in both cases).

**Table 1.** Free energies of binding calculated for compounds **A1**–**A18** and MPA.

Entry	Free energy of binding [kcal·mol^−1^]
MPA	−7.8
**A1**	−9.3
**A2**	−9.1
**A3**	−9.4
**A4**	−9.5
**A5**	−8.9
**A6**	−9.5
**A7**	−8.7
**A8**	−8.8
**A9**	−9.2
**A10**	−9.4
**A11**	−9.1
**A12**	−9.1
**A13**	−9.0
**A14**	−9.1
**A15**	−9.2
**A16**	−9.0
**A17**	−9.2
**A18**	−9.1

Aforementioned results show that the influence of heteroaromatic core, loci of amino group as well as other functionalities on the free energy of binding with IMPDH can be distinguished. Within various heteroaromatic compounds, benzimidazole-based derivative (**A3**) turned out to bound most strongly to an enzyme, followed by benzothiazole (**A1**), benzoxazole (**A2**) and finally – pyrimidine counterpart (**A7**). Within the amino group substitution variety, one can see the strongest enzyme-affinity of 6-aminated benzothiazole (**A4**), then 2- and 5-aminated equivalents (respectively: **A1** and **A5**). In the case of benzoxazole motif, 5-aminated derivative (**A6**) came out as more enzyme-binding effective than 2-aminated analogue (**A2**). Considering the structure-activity relationship for a class of novel benzothiazole derivatives (**A8**–**A18**), one can observe no clear-cut differences in the value of binding energy towards IMPDH. However, dimethylated (**A10**) and nitro (**A15**) derivatives have the highest affinity for the enzyme. Moreover, this comparison showed that the halogen derivers (**A11**–**A13** and **A18**) do not differ much from each other, even taking trifluoromethylated amide (**A14**) into this consideration. Additionally, 4-substituted compounds (**A16**–**A18**) did not differ from their 6-substituted counterparts (**A8**, **A9,** and **A12**) in case of the enzyme affinity except for the methoxy derivatives (**A8** and **A16**).

**Figure 1.**
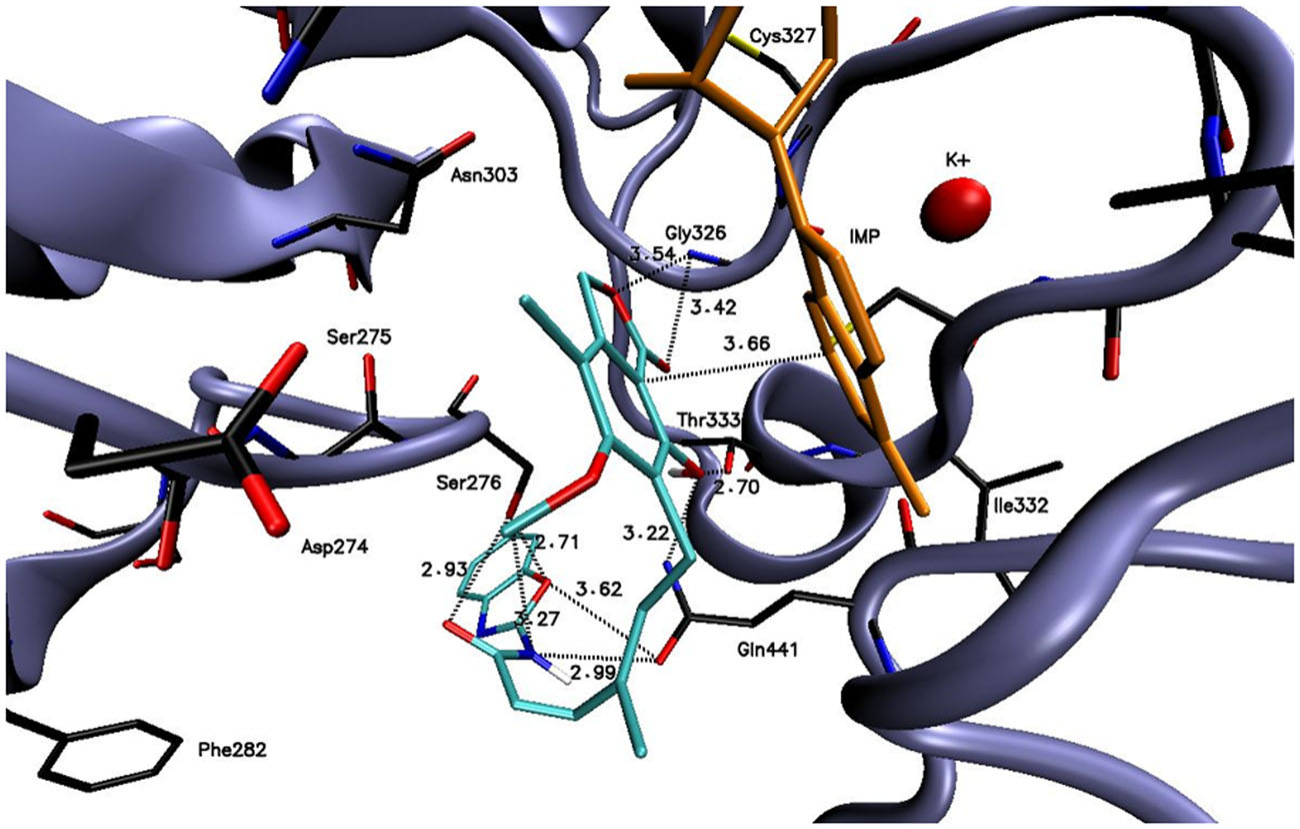
Docked binding mode and distances to the amino acid residues of IMPDH enzyme’s active site for compound **A2**.

The visualisation of the putative binding mode for the representative compound **A2** (−9.1 kcal·mol^−1^), which demonstrated the most promising activity in the biological evaluation, is shown in Figure 1. It can be noticed that compound **A2** is very well accommodated in the IMPDH active site and is surrounded by several important amino acid residues. For example, the mycophenolic part of the compound is in the close distance to the Gly326 (3.42 and 3.54 Å), Thr333 (2.70 Å), and Gln441 (3.22 Å) amino acid residues suggesting the presence of electrostatic interactions. Furthermore, the π–π stacking interaction (3.66 Å) with IMP molecules was detected. In addition, the benzoxazolyl residue of compound **A2** may also electrostatically interact with several amino acid residues, e.g. Ser276 (2.71, 2.93, and 3.27 Å), and Gln441 (2.99 and 3.62 Å). All of these plausible interactions may stabilise the enzyme-ligand complex ensuring appropriate accommodation of compound **A2** and higher inhibitory activity against target protein.

It can be seen that structure–activity relationship for the new amide derivatives of MPA is worth establishing upon biological testing, mostly due to differing free energy binding values and to see if heteroatom affects specific interactions with enzyme subunits thus facilitating further elaborations of novel immunosuppressants class, especially those based on benzothiazole motif, which turned out to be the most accessible for us and promising upon *in-silico* studies.

### Chemistry

**Scheme 1.**
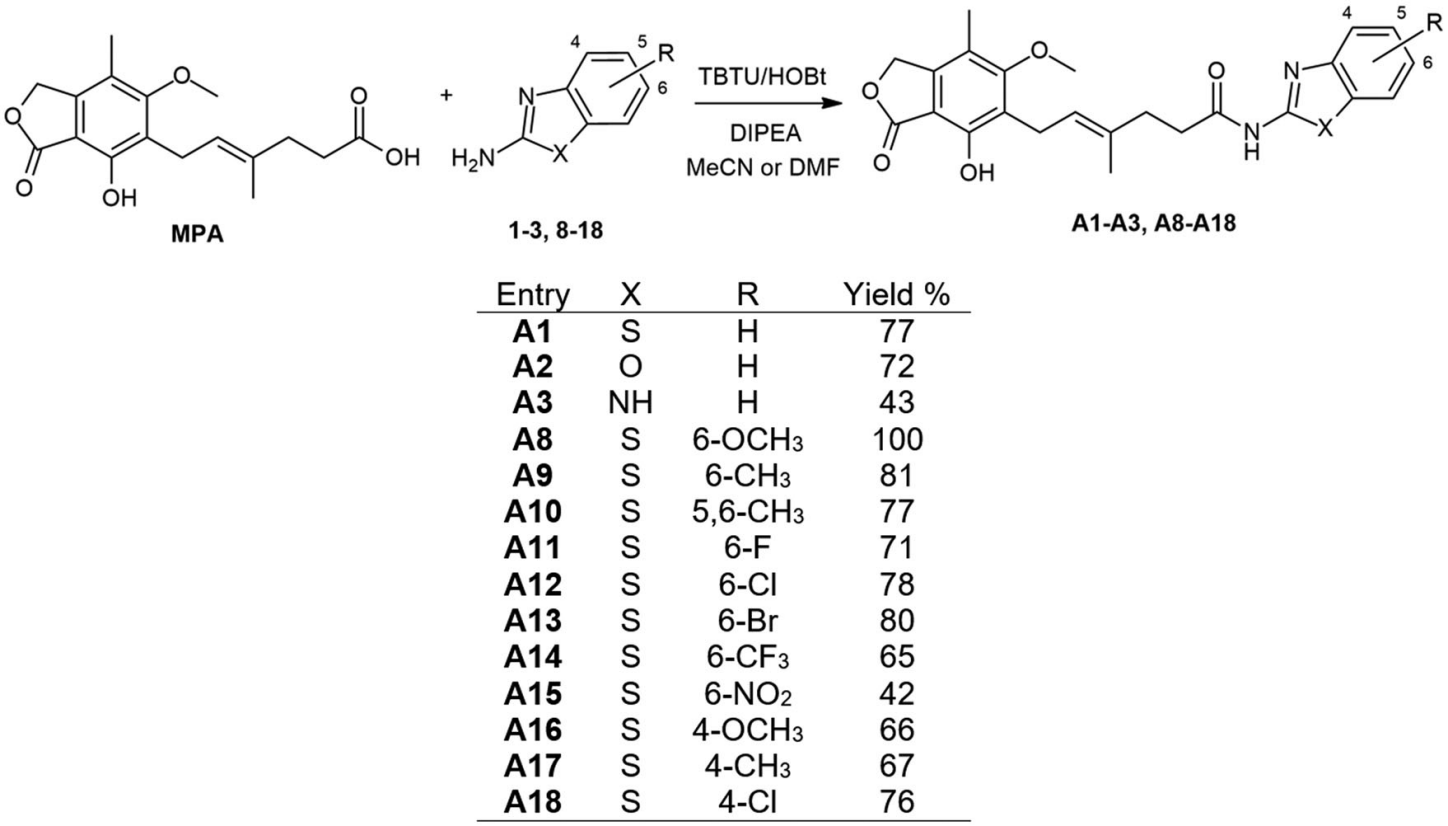
Amide derivatives of MPA synthesised *via* Method A.

**Scheme 2.**
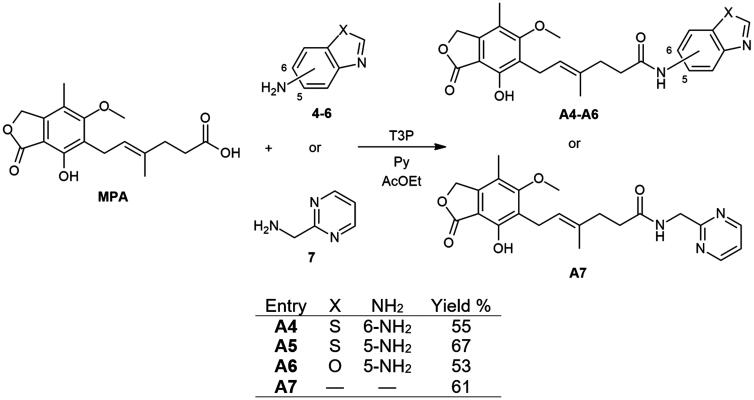
Amide derivatives of MPA synthesised through Method B.

The newly devised compounds **A1**–**A18** were obtained *via* typical reaction between carboxylic acid and amine moiety in the electrophilic activation of carboxylic acid functional group manner. Doubly activated MPA in the TBTU/HOBt system, in the presence of tertiary amine, was suggested to be more amine-accessible than in the standard system since additive presence (HOBt) enhances reactivity and reduces unreactive intermediate formation^34^. T3P-based method was used in the case of amino acid derivatives^18^ and then successfully applied for amines bearing its amine group in non-heteroaromatic region. This selectivity towards activating agent was probably due to the lack of nucleophilicity of heterocyclic amines towards activated carboxylic acid species, potential spontaneous heterocyclic tautomerisation within 2-amino-1,3-azole unit and intermediate state destabilisation resulting from immediate vicinity of heteroatoms. Albeit it needs to be thoroughly investigated since 2-aminomethylpyridines did not give desired products *via* T3P activation thus indicating other factors importance (e.g. solubility of substrates or pH imbalance). Chloroformate/tertiary amine system worked in Method A conditions however resulting in significantly lower yields, what has not been presented in this work. A general presentation of conducted syntheses and their outcomes is given in Scheme 1 for Method A and Scheme 2 for Method B.

Acetonitrile (ACN) seems to be the most promising solvent (for **A1**, **A2**, **A8**–**A18**) for its polar and aprotic properties, volatility, and non-adsorptive character. Acetone is prone to form imine species, DMF has a tendency for lingering at products’ surface while dimethyl sulfoxide (DMSO) causes removal difficulties. Non-polar solvents such as toluene, dichloromethane, or ethyl acetate (AcOEt) did not work at all, whereas polar solvents usage is at odds with electrophilicly-activated intermediates reaction mechanisms. For **A4**–**A7** cases, AcOEt worked, probably as it is well-suited for stabilising intermediate species established between non-heteroaromatic amine and **T3P**. Dichloromethane and ACN did not work during reaction’s optimisation and just like before – polar solvents are not applicable.

Most of compounds under investigation were isolated without chromatography methods. Resulting derivatives of **MPA**, namely **A1**, **A2**, **A8**–**A18**, were generally poorly soluble in ACN and hence were daringly washed with cold solvents to get rid of impurities, unreacted activating agents, and tertiary amine as well. However, some dissimilarities were observed in case of 4-substitued derivatives (**A16**–**A18**) and 2-aminobenzoxazole deriver (**A2**) regarding to 6-substituted ones. That tendency occurred probably due to different influence of substituent/heteroatom towards hydrogen bond formation thus increasing solubility in both polar and apolar solvents. Another exception was observed in **A3** case as DMF was used for enhancing originate amine solubility. This solvent was also used for the washing step which resulted in difficult to remove, solvent residual peak presence in the ^1^H NMR (nuclear magnetic resonance spectroscopy) spectrum. Examples **A4**–**A7** possess completely different solubility trends thereout different methods and solvents were involved for preparing them.

For **A1**, **A8**, **A11,** and **A14** cases, 2.0 equivalents of tertiary amine (**DIPEA**) were applied due to standard procedure present in the literature^35^. However, in most cases only 1.0 equivalent was utilised on account of reaction mechanism. Initially, each reaction was carried out in a manner consistent with the reaction mechanism, but the one proposed in the literature was also studied. Differences in the base amounts may happen due to the high stability of salt formed upon MPA and amine dissolution. Increasing the amount of DIPEA alters pH balance which affects the tendency of amine to react with activated MPA as it may less readily occur in protonated form. Base excess is believed to increase coupling rates and to disrupt hydrogen bonds responsible for conformation stabilisation which affects the availability of amino groups^36^. **A3** was synthesised with individual quantities of base and activating agent used which were developed through the optimisation process. Due to the potential N-acyl 2-aminobenzo[*d*]-1*H*-imidazole isomerisation processes observed between annular and exocyclic nitrogen atoms, two products would occur. However, ^1^H NMR spectrum proved *N*-acylation at the exocyclic nitrogen as an additional peak close to 12 ppm was observed (one annular, one amide, and one phenolic protons all deshielded in the magnetic field). Isomerisation and tautomerisation phenomena may affect reaction outcomes, e.g. decreasing yields as for **A3**.

Correlation between the character of substituent in 2-aminobenzo[d]-1,3-diazole and its condensation yield with MPA is as follows: electron withdrawing group has a potential upon diminishing reaction’s yield as it is clearly observed for **A14** and **A15** and electron donating group improves reaction’s outcomes regarding **A8**–**A10**. 6-halogenated derivatives, namely: **A11**, **A12**, and **A13**, should present quantitively worsened results as inductive withdrawing effect ought to lower nucleophilicity of amino group, however yields in this case were rather noncongruent with this tendency and showed decreased reaction outcomes with halogen electronegativity rise, **A13**, **A12**, and **A11** sequentially. Electronegativity impact was also observed in **A1**–**A3** cases since its growth developed lowered yields, nevertheless heterocyclic tautomerisation within amine’s structure cannot be neglected. Unclear correlation was noticed in 4-substituted cases, namely: **A16**, **A17**, and **A18**, as they present opposite trend. It probably results from different impact of substituent position on hydrogen bonding thus making solubility the limiting factor of reactions outcomes. Pretty convergent yields can be seen for **A4**–**A7** cases. They represent the class of non-heterocyclic amines which are characterised by different reactivity tendencies than previously mentioned examples. Aliphatic amine (**A7**) has been most successfully conjugated with **MPA** in this subgroup of derivatives as indirect impact of aromatic system does not decline nucleophilicity of amino group. In all cases, the solubility of reactants may influence reaction’s outcomes acting as both the driving force and limiting factor of reaction. Considerations on the effect of substituent nature on melting point^37^ are presented in Electronic Supplementary Information (ESI).

MPA as a fully substituted aromatic compound possesses no protons in the aromatic region. This fact simplifies amides’ ^1^H NMR spectrum analyses since newly incorporated moieties contribute aromatic and amide protons solely. All spectra are determined by the deshielded amide proton and phenolic one as well as recently introduced aromatic species. Remaining signals represent MPA structure and are sometimes shifted due to magnetic environment modifications. Some lapping may occur as implemented amines bear functional groups or spacers appearing in the same ^1^H NMR regions as MPA structural units. ^13^C NMR spectra are also characterised by MPA core and added value in the shape of newly introduced carbon atoms. Some dissimilarities in the nucleus characteristics may occur (especially in the case of amide group carbon) as well as for amine-based units. The latter one is represented by deshielded C(2) heteroaromatic carbon and C(3a)/C(7a) positions. Remaining loci differ with heteroatom electronegativity and substituent character, sometimes precluding precise carbon atoms assignment. Some ^1^H and ^13^C NMR spectra show long and short distance spin decoupling brought by fluorine atoms, namely in **A11** and **A14** cases. More detailed information about the character of heteroaromatic interactions within ^13^C NMR spectroscopy may be found in the literature^38^^,^^39^.

Present coupling reaction between MPA molecule and selected heteroaromatic amines provides a convenient method of amide bond synthesis since MPA is a multifunctional, synthetically challenging compound. Beforehand, typical synthesis exploiting the MPA molecule demanded phenolic group protection^15^^,^^40^ as it may hamper nucleophilic potential by impairing possible interference with activated carboxylic acid moiety of other molecules. Our work delivers simple, undemanding and solvently balanced synthetic protocol which in most cases does not require chromatography purification methods and more importantly, does not involve phenolic group protection/deprotection manner being in line with current mode to the synthesis of MPA derivers^41^^,^^42^. Moderate to excellent yields and spectrally traceable products’ make this approach remarkably potent and applicable in the synthesis of heterocyclic-derived amides of MPA.

### Biological investigations

**Figure 2.**
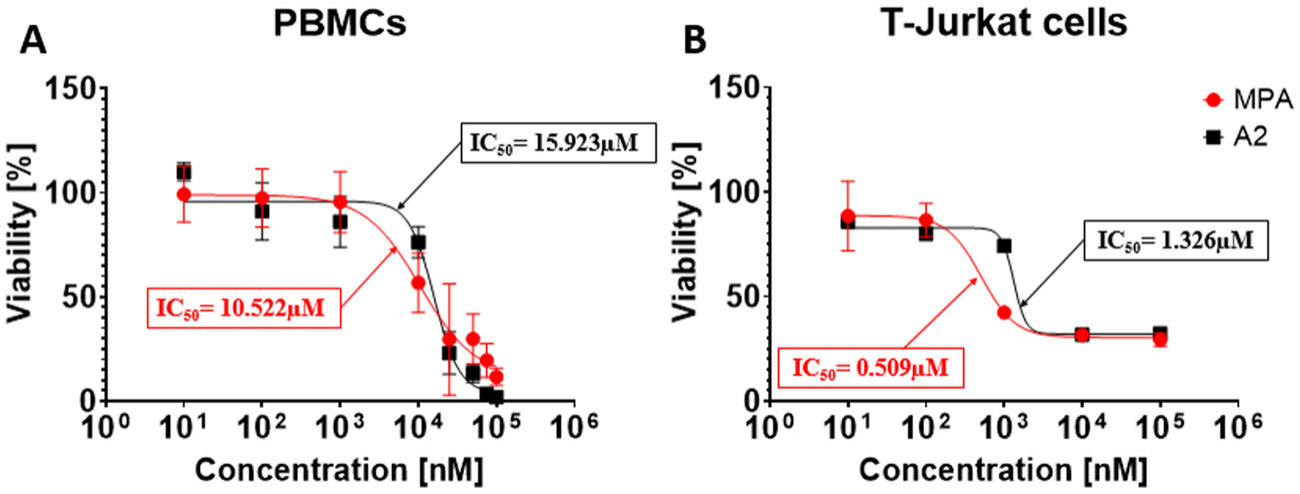
Representative dose-response curve to determine the IC_50_ of MPA and **A2**. (A) Human PBMCs for 96 h and (B) T-Jurkat cell line for 48 h were cultured in the presence of different concentrations (100; 75; 50; 25; 10; 1; 0.1 and 0.01 µM) of MPA (red) and **A2** (black). Next, plates were incubated for 24 h with XTT reagent. The conversion of water-soluble yellow tetrazolium XTT salt into orange formazan was monitored by measuring the optical density at 450 nm on microplate spectrophotometer. For XTT assay at least three experiments were performed and values are presented as mean ± SD.

**Figure 3.**
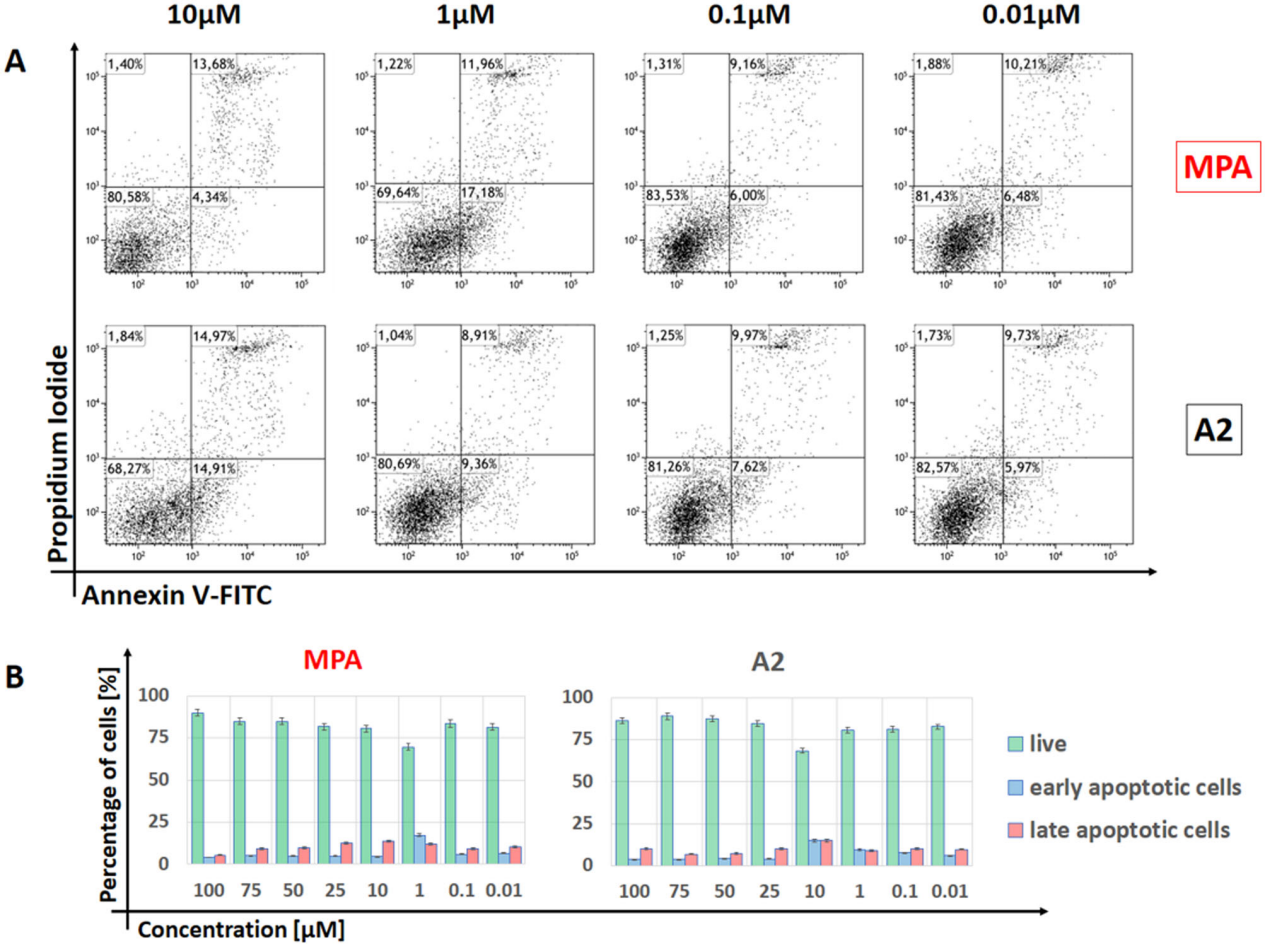
Detection of cell apoptosis in PBMCs by Annexin V-PI staining assay using flow cytometry. (A) Representative scatter plots of cells treated with different concentrations (10; 1; 0.1 and 0.01 µM) of MPA and **A2** for 72 h and stained with Annexin V-FITC and propidium iodide (PI). (B) Cells divided into three groups according to Annexin V/PI results: living cells (Annexin − PI−); early apoptotic cells (Annexin + PI−) and late apoptotic cells (Annexin + PI+).

**Figure 4.**
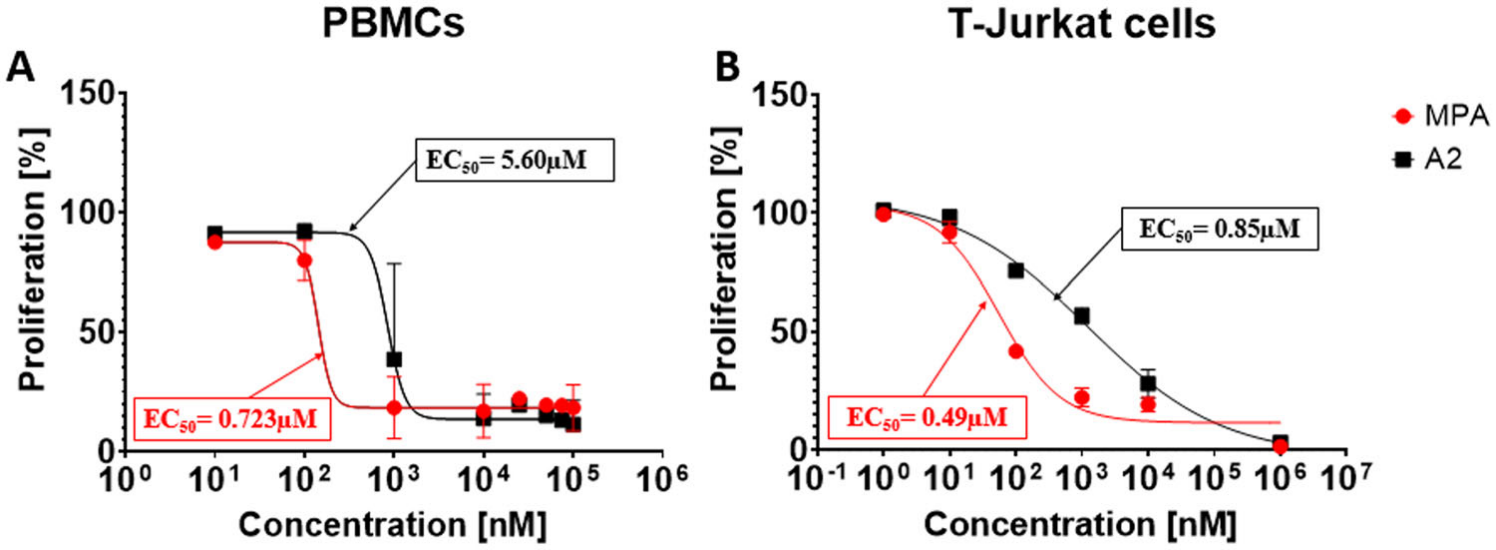
Representative dose-response curve to determine the EC_50_ of MPA and **A2**. (A) VPD 450-labelled human PBMCs were cultured in the presence of different concentrations (100; 75; 50; 25; 10; 1; 0.1 and 0.01 µM) of MPA (red) and **A2** (black) and stimulated with magnetic beads coated with anti-CD3 and anti-CD28 antibodies for 72 h. (B) VPD 450-labelled T-Jurkat cells were cultured in the presence of different concentrations (100; 10; 1; 0.1 and 0.01 µM) of MPA (red) and **A2** (black) for 48 h. Cell proliferation was analysed using flow cytometry. The results are expressed as the mean ± SD.

Drug toxicity that affects the cells can cause a variety of serious side effects, including uncontrolled suppression of the immune system. In order to check the cytotoxicity of the tested compounds, the XTT test was performed, the results of which are presented in Table 2. All tested compounds were characterised by lower cytotoxicity than native MPA. Unfortunately, several compounds (marked with **↓**) were partially insoluble in culture despite surfactant addition. They were investigated too, but one could not use them as an immunosuppressive drug in their current form. Taking into account the EC_50_ values obtained in the proliferation tests (VPD540 staining, Table 3), the compound **A2** was selected as the one with the highest immunosuppressive potential and the possibility of its later use in therapy, and is presented in the graphs (Figures 2–4).

**Table 2.** IC_50_ [µM] values of derivatives of MPA based on XTT test.

Entry	PBMCs		T-Jurkat	
	IC_50_ [μM]	Viability [%]	IC_50_ [μM]	Viability [%]
MPA	10.522 ± 0.015	–	0.509 ± 0.008	–
**A1**	74.570 ± 0.040*	–	–	68
**A2**	15.923 ± 0.009	–	1.326 ± 0.002	–
**A3**	94.538 ± 0.047	–	–	74
**A4**	24.639 ± 0.015	–	37.789 ± 0.002	–
**A5**	45.730 ± 0.023	–	75.040 ± 0.005	–
**A6**	55.487 ± 0.028	–	31.407 ± 0.002	–
**A7**	70.480 ± 0.035	–	10.739 ± 0.003	–
**A8**	93.196 ± 0.047 (↓)	–	51.124 ± 0.008	–
**A9**	94.600 ± 0.047 (↓)	–	85.700 ± 0.004	–
**A10**	129.408 ± 0.064	–	–	51
**A11**	139.858 ± 0.070* (↓)	–	–	56
**A12**	62.975 ± 0.031* (↓)	–	12.833 ± 0.008*	–
**A13**	63.173 ± 0.032*	–	58.069 ± 0.002	–
**A14**	81.482 ± 0.041*	–	34.749 ± 0.003	–
**A15**	137.633 ± 0.069* (↓)	–	12.717 ± 0.005	–
**A16**	54.578 ± 0.027 (↓)	–	–	69
**A17**	– (↓)	65	–	82
**A18**	74.426 ± 0.037*	–	10.261 ± 0.001	–

Significance was calculated using the *T*-test; significant results are marked with *(*p* < 0.05) with MPA as a control; (↓) – precipitating compound.

**Table 3.** EC_50_ [µM] values of derivatives of MPA based on dye-based proliferation assay VPD450.

Entry	PBMCs		T-Jurkat	
	EC_50_ [μM]	Viability [%]	EC_50_ [μM]	Viability [%]
MPA	0.723 ± 0.011	–	0.490 ± 0.002	–
**A1**	–	82	–	–
**A2**	5.601 ± 0.022	–	0.850 ± 0.004	–
**A3**	119.600 ± NAN**	–	–	55
**A4**	62.260 ± 0.010	–	14.730 ± 0.074	–
**A5**	76.980 ± 0.010*	–	36.050 ± 0.180	–
**A6**	58.270 ± 0.021	–	13.840 ± 0.069	–
**A7**	29.450 ± 0.025	–	9.510 ± 0.047	–
**A8**	133.500 ± 0.007*(↓)	–	22.040 ± 0.110	–
**A9**	136.200 ± 0.011(↓)	–	22.720 ± 0.113	–
**A10**	85.240 ± 0.012**	–	26.690 ± 0.133	–
**A11**	83.070 ± 0.012**(↓)	–	63.210 ± 0.316	–
**A12**	38.370 ± 0.017(↓)	–	12.160 ± 0.061	–
**A13**	39.470 ± 0.013	–	12.640 ± 0.063	–
**A14**	30.710 ± 0.008	–	12.580 ± 0.063	–
**A15**	44.590 ± 0.013(↓)	–	13.370 ± 0.067	–
**A16**	46.290 ± 0.011(↓)	–	–	73
**A17**	82.140 ± NAN***(↓)	–	–	69
**A18**	14.700 ± 0.020	–	11.280 ± 0.056	–

Significance was calculated using the T-test; significant results are marked with *(*p* < 0.05), **(*p* < 0.01), or ***(*p* < 0.001) with MPA as a control; NAN – not a number; (↓) – precipitating compound.

In order to test the immunosuppressive effect of MPA derivatives, we decided to use T-Jurkat cell line as a model of human lymphocytes and PBMCs which are widely used in research and toxicology applications as *in vitro* model of the immune response, and provides answers to questions about the toxicity of potential new drug compounds.

#### Determination of cytotoxic activity using XTT method

Table 2 is collected IC_50_ values of tested derivatives on PBMCs and T-Jurkat cells under XTT assay conditions. In the case of the PBMC approach, each derivative exhibits lower cytotoxic properties than reference (MPA). Similar conclusions may be drawn from assay performed on T-Jurkat cell line albeit **A2** possesses approximate IC_50_ value to MPA. Due to the limited solubility of **A8**, **A9**, **A11**, **A12**, **A15**, **A16,** and **A17** one could not receive reliable results for PBMCs. In the T-Jurkat-based method, some IC_50_ values were not obtained and thus cell viability was measured at the highest concentration using trypan blue exclusion (for **A1**, **A3**, **A10**, **A11**, **A16,** and **A17** namely). Even despite the impossibility of accurate results showing in each case, the general tendency shows that the incorporation of benzothiazole, benzoxazole, benzimidazole, and pyrimidine systems decrease cytotoxicity and in terms of precise values it is usually several dozen times lowered comparing to the reference compound. The cytotoxicity was influenced by amino group position in heterocyclic motifs. Entries **A1**, **A4,** and **A5** show that 6-aminated benzothiazole affects cytotoxicity stronger than 5- and 2-aminated ones in succession. Similar effects may be seen in entries **A2** and **A6** for aminated benzoxazole. Correlation between the character of substituent and cytotoxic effect is unclear, however these possessing electron-rich substituents seem to be more effective than the neutral ones (**A13**, **A14,** and **A18** *versus* **A10**). The tendency of IC_50_ values in T-Jurkat cells can be determined similarly, however 6-methoxy (**A8**) as well as 6-nitro (**A15**) and 6-methyl (**A9**) derivatives show: the more electron-rich group is attached, the stronger the cytotoxic effect of the corresponding derivative. Halogen impact may be noticed in this case as in the group **A12**–**A14** the increase of electronegativity builds cytotoxicity up. Little difference between 6-substituted and 4-substituted derivatives (**A12** and **A18**) may be noted.

Eventually, **A2** is the most invasive derivative as it affects both PBMCs and T-Jurkat cells, however still weaker than the reference compound. Remaining amides are clearly less toxic than MPA and some correlations between the type of substituent, loci of amino group and cytotoxicity may be observed. Electron-rich moieties seem to enhance derivers’ toxicity towards exploited cells, albeit this relation is unclear since limited by solubility of tested amides in conditions given.

Figure 2 presents the response curve (concentration of tested compound is assigned to viability of cells) prepared for a few different concentrations of MPA and **A2** in a suitable medium. On the basis of these measurements one can determine optimal IC_50_ value for referential MPA and the most promising compound **A2**. As it was mentioned before, **A2** possesses lowered cytotoxicity towards human and T-Jurkat cells which is represented by this graph. In general, plots are quite convergent in the lowest and highest concentrations and differ evidently in the medium range. Noteworthy, amide **A2** indicated the highest antiproliferative activity within the designed compounds (Table 3) together with lower cytotoxicity in comparison to parent MPA (Table 2).

#### Effect of MPA and A2 on apoptosis

The apoptotic effects of MPA and **A2** were evaluated by flow cytometry after staining with Annexin V and PI. Annexin V can be detected in early and late stages of apoptosis, whereas PI can only be detected in late apoptosis or necrosis. Due to the level of necrotic cells at about 1%, it was not taken into account during the analysis. As shown in Figure 3, treatment with compound **A2** and MPA as a control for 96 h, late apoptosis cells could be observed mainly when the cells were treated with a concentration close to the EC_50_ value (0.723 µM for MPA and 5.601 µM for **A2**). In the case of MPA in a concentration of 10 µM, one can observe the presence of over 13% of late apoptosis cells, but only about 4% of early apoptosis cells. This situation changes significantly in the case of a 10-fold lower concentration, where over 17% of early apoptosis cells and over 11% of late apoptosis can be found. Derivative **A2** showed a similar effect, however, at a concentration of 10 µM, where one can see more than 14% of early and late apoptosis cells. It also indicated that the inhibition of cell proliferation, even at the 10 μM concentrations (Figure 3(B)), was mainly due to the influence of tested compounds.

#### Determination of antiproliferative activity using Violet Proliferation Dye (VPD) 450 in flow cytometry

The antiproliferative effects of MPA and tested compounds were evaluated by flow cytometry after staining with VPD450 which is one of the recommended dyes, characterised by a relatively low toxicity and the possibility of observing cell division through the loss of a fluorescent dye^43^. The representative dose-response curve to determine the EC_50_ of MPA and **A2** are presented at Figure 4 and exemplary histograms with appropriate microscopic pictures are provided in ESI (ESI supplementary Figure 1). As it can be observed in Table 3, all compounds have an EC_50_ value greater than MPA for both PBMCs and T-Jurkat cells. Compound with the most activity is **A2** with an EC_50_ value 5.601 µM. However, taking into account that this compound is characterised by lower cytotoxicity than MPA, it may constitute a suitable candidate for future research. Additionally, there was no change in cell proliferation when they were treated with DMSO and the solubilisation additive, suggesting that the observed reduction of proliferation was exclusively due to the presence of MPA or tested compounds in the cultures.

Results presented in Table 3 show similar tendencies as in Table 2. Unambiguous outcomes may be observed by comparing PBMCs and T-Jurkat cells’ behaviour towards tested compounds as they exhibit convergent trends. For the first conditions, one can notice loci differentiation in case of 6- and 5-aminated benzothiazole-derived amides (namely **A4** and **A5**) for the first instance presenting higher antiproliferative properties than the second one. Under these circumstances, 2-aminated deriver’s properties (**A1**) could have not been determined under PBMC culture medium. **A5** shows worsened effectivity in comparison to **A2** (that strongly affects human cells) thus exposing again amino group loci importance at bioactivity matter. In terms of the electronic nature of substituents, one can discern a clear-cut difference between 6-halogenated derivatives of benzothiazole and remaining ones. 6-chloro (**A12**), 6-bromo (**A13**), and 6-trifluoromethyl (**A14**) cases manifest significantly increased inhibitory properties towards PBMCs than 6-methoxy (**A8**) and 6-methyl (**A9**) examples, whereas 6-fluoro (**A11**) does not fit into this trend revealing poor antiproliferative features. Loci of chlorine atom or methoxy group may also affect bioactivity as 4-chloro (**A18**) and 4-methoxy (**A16**) derivatives present good affinity towards cell division rupture (see 6-chlorinated and 6-methoxylated counterparts, **A12** and **A8,** respectively). T-Jurkat cell line assay reveals the same leaning, nevertheless displaying lowered EC_50_ values for 6-methoxy (**A8**), 6-methyl (**A9**), 5,6-dimethyl (**A10**), and 6-nitro (**A15**) derivers.

Ultimately, these biological studies have shown that none of the synthesised amides exhibit antiproliferative properties greater than reference compound, however one of them – **A2**, reveals close enough value of EC_50_ to MPA in both tests. Further 2-aminobenzoxazole-derived compounds’ functionalisation need to be proceeded and bioactivity of resulting derivers should be performed. Structure–activity relationship has shown unclear correlation, though more electron-rich substituents tend to affect EC_50_ value stronger than neutral ones, there again fluorinated amide does not incorporate into this trend. Rapid change in EC_50_ values ratio for **A8**:**A12** pair for PBMCs and for T-Jurkat cells approach, from 3.48 to 1.81, reveals obvious differences in tests’ complexity as compounds may be shielded by medium components.

Comparison of antiproliferative activity measurements of MPA and **A2** was presented in Figure 4. Plots convergence is slightly inferior than this formed for IC_50_ value figure, especially in the smallest and highest concentrations range. In case of T-Jurkat cells, half maximal effective concentration is more concurrent than for PBMCs. PBMC proliferation measurements done for **A2** and MPA reflect on highly similar antiproliferative properties as staining intensity of PBMCs and cell division’s histograms are almost identical (see ESI supplementary Figure 1). It may have positive repercussions for further 2-aminobenzoxazole and MPA derivatives biological evaluations.

The reported compounds have shown eminently lowered cytotoxic effects on both PBMCs and T-Jurkat cells while at the same time exhibiting weaker antiproliferative properties than referential MPA. Despite limited solubility, measurements were generally possible with surfactant addition. Importantly, Tween 20 does not influence the bioactivity at all. Structure–activity relationship for these compounds is unclear, however electron-rich substituents tend to both increase cytotoxicity and antiproliferative properties as well. Fluorine atom does not fit into this correlation, probably due to specific substrate-enzyme interactions occurrence. Loci differentiation may be observed especially in varying amino group position, albeit 4-substituted 2-aminobenzothiazoles seem to affect cell lines stronger than its 6-substituted counterpart, extending the scope of the effect of substituent on bioactivity.

**Figure 5.**
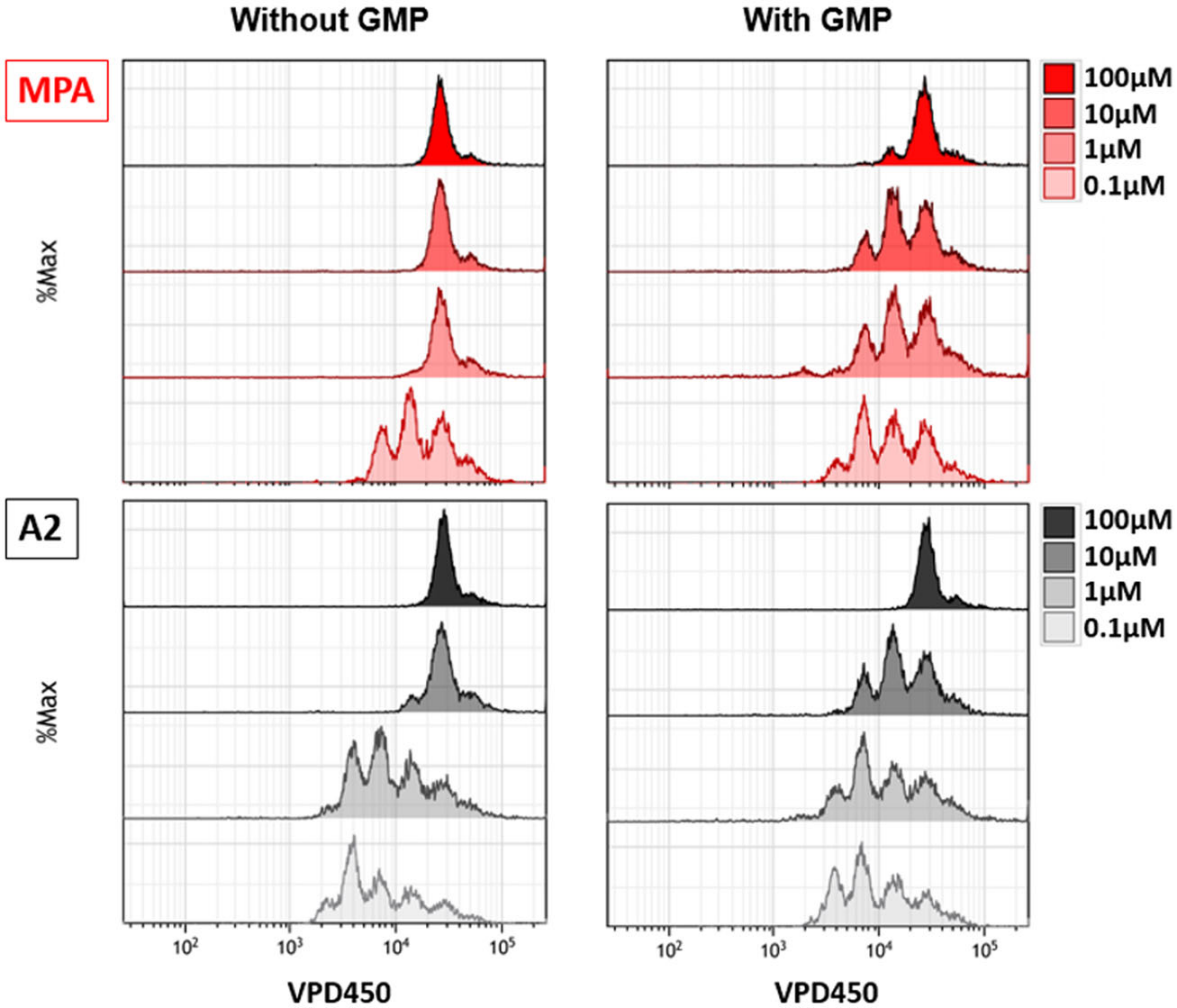
Representative antiproliferative activity of MPA and **A2**. VPD 450-labelled human PBMCs, in the presence of different concentrations (100; 10; 1 and 0.1 µM) of MPA (red) and **A2** (black) and stimulated (magnetic beads coated with anti-CD3 and anti-CD28 antibodies) were cultured with or without the addition of 50 µM GMP for 72 h. Cell proliferation was analysed using flow cytometry.

Subsequently, we verified if designed MPA derivatives act as IMPDH inhibitors. According to reported experiment^44^, suppression of cell proliferation caused by IMPDH inhibitors in the presence of guanosine source, e.g. gunosine monophosphate (GMP) is reversible. To this test were selected MPA (reference), **A2** as the most active target amide (Figure 5), and **A18** (active among benzothiazoles), and **A7** as possessing another heterocyclic ring (see ESI supplementary Figure 2). The both Figures show the proliferative activity of the tested compounds against PBMCs at the appropriate concentrations. The mechanism of action of the derivatives was consistent with that of MPA. In the concentration range where inhibition of proliferation occurs without the addition of GMP (for MPA: 100; 10 and 1 µM, and for **A2**: 100 and 10 µM), it can be seen that proliferation increases after the addition of GMP.

In the end, **A2** has shown the strongest antiproliferative properties for exhibiting similar properties as MPA. Its lowered cytotoxicity and better solubility can be an added value for determining new 2-aminobenzoxazole and MPA derivatives properties. Its similarity in PBMCs proliferative response to the referential compound renders it into being a potent immunosuppressant, albeit further investigations must be proceeded. The presence of nitrogen and oxygen atoms in newly incorporated amine fragment in **A2** may indicate the influence of these elements on the bioactivity of new MPA derivatives since it is present in MMF – the most common and wide-spread drug used in graft rejection prophylaxis. In the sulphur atom case (low cytotoxic 2-aminobenzothiazole derivatives), its presence significantly decreases antiproliferative properties, however, it may affect further biological assays in the context of zinc-based enzymes, similarly to reported in literature HDAC inhibitors^45–47^.

## Summary

The series of 18 novel amide derivatives of MPA were designed for their affinity towards IMPDH in the enzyme’s active site, employing *in-silico* assessment. The synthesis was optimised with uronium (TBTU/HOBt) or phosphonic acid anhydride (T3P) electrophilic activation systems, for carboxylic acid moiety, which are widely available and easy accessible, and were well adjusted for aliphatic, aromatic, and heteroaromatic amines to give target amides in moderate to excellent yields mostly without the need of chromatographic purification. Subsequently, biological evaluation revealed lowered cytotoxicity of all prepared amides (**A1**–**A18**) in relation to the MPA molecule, both for T-Jurkat and PBMC cell lines while **A2** presented the most promising antiproliferative activity. Structure–activity relationship for differently substituted benzothiazoles and benzoxazoles was devised and indicated that amino group or substituent loci, electron-character of functionalities, and heteroatoms electronegativity might affected IC_50_ and EC_50_ values of newly synthesised derivers. These results confirm that the incorporation of aforementioned heterocyclic structural motifs into MPA core relevantly changed cytotoxicity and antiproliferative properties. Benzoxazole moiety in **A2** turned out to be the most efficient in bioactivity alteration thus inclining further biological investigations in terms of different antiproliferative activities and structure–activity relationship determining. Benzothiazole MPA derivatives gave lower cytotoxicity. On the other hand, diminished toxicity against healthy cells may provide active substance with improved therapeutic properties, including less side effects similarly to conjugate of MPA with quinic acid^16^. Moreover, investigations on both benzoxazole and benzothiazole MPA derivatives can lead to chemotherapeutics with anticancer potential^32^^,^^33^ causing them interesting structures for further exploring.

## Experimental section

### Computational *studies*

#### Ligands preparation

Prior to docking procedures, the potential inhibitors were prepared using Portable HyperChem 8.0.7 Release (Hypercube, Inc., Gainesville, FL^48^). Each ligand was optimised using a MM + force field^49^ and the Polak–Ribiere conjugate gradient algorithm (terminating at a gradient of 0.05 kcal mol^−1 ^Å ^−1^).

#### Protein preparation

The X-ray structures of the IMPDH II enzyme used for molecular modelling studies were taken from the Protein Databank (Protein Data Bank accession codes**: 1JR1).** The docking analysis was carried out after standard preparation procedures including removal of chain B of initial receptor structure, deletion of co-crystalised MPA structure and water molecules, and addition of hydrogen atoms and Gasteiger charges to each atom.

#### Molecular docking

Docking calculations of the optimised ligands to the prepared structure of human IMPDH II enzyme was carried out with Autodock Vina version 1.1.2 software (The Molecular Graphic Laboratory, The Scripps Research Institute, La Jolla, CA)^50^. For all of docking studies, a grid box size was centred on the Cβ atom of Ser276, and the size of the grid box was 25 Å × 25 Å × 25 Å. Then, the best poses for a particular ligand were visually inspected. Illustrations of the 3D model were generated using VMD version 1.9 (University of Illinois at Urbana – Champaign, Urbana, IL)^51^.

### Chemistry

#### General methods and materials

MPA, amines (**1–18**) used for **A1**–**A18** synthesis, *N*,*N*-diisopropylethylamine (DIPEA), 1-hydroxybenzotriazole monohydrate (HOBt·H_2_O), 2-(1*H*-benzotriazole-1-yl)-1,1,3,3-tetramethylaminium tetrafluoroborate (TBTU), 50% propylphosphonic acid anhydride in *N*,*N*-dimethylformamide (DMF; T3P) solution and pyridine (Py) were purchased from Sigma Aldrich, Apollo Scientific, AmBeed, Maybridge, Acros, Angene, Chemat, and Alfa Aesar and used as is. Organic solvents are commercially available on Sigma Aldrich, Fisher Scientific, Avantor, and Chempur stores. ACN, AcOEt, *N*,*N*-DMF, 1,4-dioxane (1,4-D), and Py were purified and dried upon standard procedures. Melting points (uncorrected) were measured using the Stuart SMP30 cryometer. ^1^H and ^13^C NMR spectra were recorded on the Varian Unity Inova 500 spectrometer on the operating frequencies 500 MHz for ^1^H NMR and 126 MHz for ^13^C NMR assays. Chemical shifts (δ) are given in parts per million (ppm) in relation to tetramethylsilane (TMS) with the residual solvent peak as internal standard (DMSO–d_6_: 2.50 ppm for ^1^H NMR and 39.52 ppm for ^13^C NMR). Coupling constants (**J**) are shown in hertz (**Hz**). Residual solvent peaks, especially from ACN, dichloromethane, *N*,*N*-DMF, AcOEt, toluene, and water, may be observed and appear in typical values of chemical shifts. The description of individual peaks within ^1^H and ^13^C NMR spectra was developed on the basis of the characteristic structural motifs present in MPA structure and the standard numbering brought under benzo[*d*]-1,3-azoles. Thin layer chromatography (TLC) analyses were carried out on Supelco silica gel TLC aluminium foils with fluorescence indicator 254 nm, whereas column chromatography was performed on silica gel 60 (230–450 Mesh, Alfa Aesar, Haverhill, MA). Visualisation of the chromatogram was achieved by UV light (254 nm) exposition or iodine staining.

HPLC analyses were performed using Agilent liquid chromatograph series 1290 (Agilent Technology, Waldbronn, Germany) consisting of binary pump G4220A, autosampler G4226A, thermostated column compartment G1316C, diode-array detector G1315C. The chromatographic system was controlled with Agilent MassHunter soft-ware B 06.01. The samples (2 μL) were injected onto a Poroshell EC-C18 2.7 µm (3.0 mm × 150 mm) column thermostated at 40 °C. The mobile phase flow rate was 0.4 mL·min^−1^, and elution was performed using 0.1% (v/v) formic acid in water (solvent A) and MeCN/MeOH (1:1; v/v) (solvent B) in gradient mode: from 50% B to 100% B in 25 min. The UV signal was registered at 254 nm.

The accurate mass of the tested amides (**A1**–**A18**) and MPA was confirmed by electrospray ionisation quadrupole time-of-flight mass spectrometry ESI(-)-QTOF analysis using an Agilent 1290 LC system coupled to the Agilent QTOF mass spectrometer G6540 (Santa Clara, CA) operated in negative ionisation scan mode.

MPA characteristics present as follows: mp. 143–145 °C; ^1^H NMR (DMSO–d_6_) δ: 12.01 (s, 1H, –COOH), 9.40 (s, 1H, Ar–OH), 5.24 (s, 2H, Ar–CH_2_O–), 5.13 (t, *J* = 6.9 Hz, 1H, >C = CH–), 3.69 (s, 3H, Ar–OCH_3_), 3.29 (d, *J* = 6.8 Hz, 3H, Ar–CH_2_–), 2.26 (t, *J* = 7.4 Hz, 2H, –CH_2_–), 2.16 (t, *J* = 7.5 Hz, 2H, –CH_2_), 2.08 (s, 3H, Ar–CH_3_), 1.74 (s, 3H, –CH_3_); ^13^C NMR (DMSO–d_6_) δ 174.52 (–COOH), 170.58 (–COO–), 163.01 (C_Ar_–OMe), 153.16 (C_Ar_–OH), 146.27 (C_Ar_–CH_2_O–), 134.04 (>C = C(–CH_3_)–), 123.13 (C_Ar_–CH_2_–CH=), 122.85 (>C = C(–CH_3_)–), 116.40 (C_Ar_–CH_3_), 107.41 (C_Ar_–COO–), 69.06 (Ar–CH_2_O–), 61.08 (Ar–OCH_3_), 34.57 (–CH_2_–COOH), 32.91 (–CH_2_–CH_2_–), 22.87 (Ar–CH_2_–), 16.46 (=C(–CH_3_)–), 11.51 (Ar–CH_3_); R_T,HPLC_ = 7.259 min, purity 100.00%; HRMS (*m/z*) calculated for C_17_H_20_O_6_ [M — H]^−^: 319.1182; found: 319.1346.

#### General methods for A1–A18 synthesis

We used two methods for syntheses of amide-like derivatives of MPA. Method A is intended for benzo-1,3-azoles aminated in the heteroaromatic region while Method B is dedicated for homoaromatic analogues. There are some differences in solvents used for carrying out these syntheses as well as some dissimilarities in applied reagents quantities. They are specified for particular cases.

##### Method A

In oven-dried, round bottom flask were placed 50 mg of MPA (0.156 mmol, 1.0 eq) and 27 mg of 97% 2-aminobenzo[*d*]thiazole (0.172 mmol, 1.1 eq). Afterwards, solids were dissolved in 1.5 mL of dry ACN and 0.05 mL of dry *N,N*-DMF. After being placed in an inert gas atmosphere (preferable argon) and cooled to 0 °C with an ice bath, 55 μL of *N*,*N*-DIPEA (*d* = 0.742 g/mL, 40.3 mg, 0.312 mmol, 2.0 eq) was added via syringe to the reaction system. Then, 24 mg of HOBt·H_2_O (0.156 mmol, 1.0 eq) and 51 mg of 2-(1*H*-benzotriazole-1-yl)-1,1,3,3-TBTU (0.156 mmol, 1.0 eq) were added in one portion. Reaction mixture was allowed to heat to ambient temperature and was stirred for at least 48 h (preferably 72 h). Then, it was quenched with 0.4 mL of methanol, well mixed and filtered on the Hirsch funnel. White precipitate was thoroughly washed with cold solvents: ACN (ca. 2.0 mL), methanol (ca. 1.0 mL), and dichloromethane (ca. 1.0 mL) sequentially and well dried. White precipitate was then used without further purification while filtrate was discarded. **A1** was obtained as off-white solid with 77% yield (54 mg).

##### Method B

In oven-dried, round bottom flask was placed 125 mg of MPA (0.390 mmol, 1.0 eq) and 65 mg of 97% 6-aminobenzo[d]thiazole (0.429 mmol, 1.1 eq). Then, solids were dissolved in 2.0–3.0 mL of dry AcOEt and the reaction system was cooled down to 0 °C with an ice bath. Under an inert gas atmosphere, 63 μL of dry Py (*d* = 0.982 g/mL, 61.7 mg, 0.780 mmol, 2.0 eq) was added *via* syringe to the reaction mixture. Afterwards, 342 μL of 50% solution of propylphosphonic acid anhydride in N,N-DMF (T3P) (*d* = 1.09 g/mL, 186.2 mg, 0.585 mmol, 1.5 eq) was added dropwise in 30 min. Then it was allowed to stir for at least 48 h (preferably 72 h) at room temperature. This mixture was then diluted with 5 mL of fresh AcOEt, extracted with 1 M hydrochloric acid solution, saturated sodium carbonate solution and then washed with brine. Organic phase was dried over anhydrous magnesium sulphate, filtered off, concentrated under reduced pressure and then purified over column chromatography in toluene/AcOEt system (2/1, v/v, *R_F_* = 0.13). **A4** was obtained as a fluffy, ecru solid with 55% yield (97 mg).

##### N-(benzo[d]thiazol-2-yl) mycophenolate (A1)


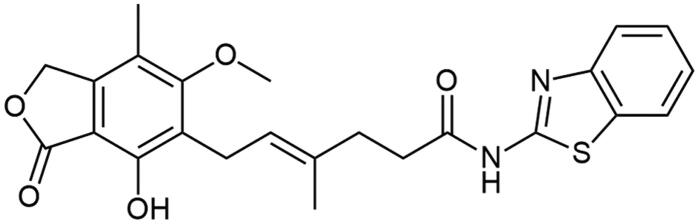
Method A – 2.0 eq DIPEA, afforded the product as white solid with 77% yield; mp. 205–210 °C; ^1^H NMR (DMSO–d_6_) δ 12.23 (s, 1H, –NH–), 9.35 (s, 1H, Ar–OH), 7.94 (d, *J* = 7.8 Hz, 1H, CH_HetAr_), 7.71 (d, *J* = 8.0 Hz, 1H, CH_HetAr_), 7.43 (t, *J* = 7.6 Hz, 1H, CH_HetAr_), 7.30 (t, *J* = 7.5 Hz, 1H, CH_HetAr_), 5.18 (t, *J* = 6.4 Hz, 1H, >C = CH–), 5.04 (s, 2H, Ar–CH_2_O–), 3.65 (s, 3H, Ar–OCH_3_), 3.28 (d, *J* = 6.7 Hz, 2H, Ar–CH_2_–), 2.56 (t, *J* = 7.4 Hz, 2H, –CH_2_–), 2.29 (t, *J* = 7.2 Hz, 2H, –CH_2_–), 1.96 (s, 3H, Ar–CH_3_), 1.78 (s, 3H, –CH_3_); ^13^C NMR (DMSO–d_6_) δ 172.19 (–CONH–), 170.56 (–COO–), 162.83 (C_Ar_–OMe), 158.25 (C(2)_HetAr_), 153.06 (C_Ar_–OH), 148.91 (C(3a)_HetAr_), 146.08 (C_Ar_–CH_2_O–), 133.62 (>C = C(–CH_3_)–), 131.87 (C(7a)_HetAr_), 126.45 (C(5)_HetAr_), 123.83 (C(6)_HetAr_), 123.64 (C_Ar_–CH_2_–CH=), 122.65 (>C = C(–CH_3_)–), 122.02 (C(7)_HetAr_), 120.85 (C(4)_HetAr_), 116.23 (C_Ar_–CH_3_), 107.26 (C_Ar_–COO–), 68.88 (Ar–CH_2_O–), 60.97 (Ar–OCH_3_), 34.64 (–CH_2_–CONH–), 34.33 (–CH_2_–CH_2_–), 22.88 (Ar–CH_2_–), 16.50 (=C(–CH_3_)–), 11.40 (Ar–CH_3_); R_T,HPLC_ = 12.253 min, purity 98.95%; HRMS (*m/z*) calculated for C_24_H_24_N_2_O_5_S [M — H]^−^: 451.1328; found: 451.1572.

##### N-(benzo[d]oxazol-2-yl) mycophenolate (A2)


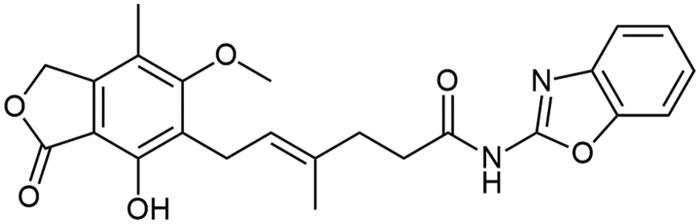
Method A – 1.0 eq DIPEA, washing step limited to cold ACN usage, afforded the product as white solid with 72% yield; mp. 175–177 °C; ^1^H NMR (DMSO–*d_6_*) δ 11.57 (bs, 1H, –NH–), 9.40 (bs, 1H, Ar–OH), 7.64 − 7.49 (m, 2H, CH_HetAr_), 7.28 (m, 2H, CH_HetAr_), 5.22 − 5.13 (m, 3H, >C = CH– and Ar–CH_2_O–), 3.67 (s, 3H, Ar–OCH_3_), 3.30 (d, *J* = 6.8 Hz, 2H, Ar–CH_2_–), 2.58 (m, 2H, –CH_2_–), 2.27 (t, *J* = 7.5 Hz, 2H, –CH_2_–), 2.02 (s, 3H, Ar–CH_3_), 1.78 (s, 3H, –CH_3_); ^13^C NMR (DMSO–d_6_) δ 170.56 (–COO–), 162.95 (C_Ar_–OMe), 155.49 (C(2)_HetAr_), 153.12 (C_Ar_–OH), 147.96 (C(7a)_HetAr_), 146.20 (C_Ar_–CH_2_O–), 141.10 (C(3a)_HetAr_), 133.84 (>C = C(–CH_3_)–), 124.92 (C(6)_HetAr_), 123.86 (C(5)_HetAr_), 123.48 (C_Ar_–CH_2_–CH=), 122.77 (>C = C(–CH_3_)–), 118.55 (C(4)_HetAr_), 116.33 (C_Ar_–CH_3_), 110.38 (C(7)_HetAr_), 107.36 (C_Ar_–COO–), 68.99 (Ar–CH_2_O–), 61.03 (Ar–OCH_3_), 35.08 (–CH_2_–CONH–), 34.50 (–CH_2_–CH_2_–), 22.89 (Ar–CH_2_–), 16.57 (=C(–CH_3_)–), 11.45 (Ar–CH_3_); R_T,HPLC_ = 9.474 min, purity 98.96%; HRMS (*m/z*) calculated for C_24_H_24_N_2_O_6_ [M — H]^−^: 435.1556; found: 435.1793.

##### N-(1H-benzo[d]imidazol-2-yl) mycophenolate (A3)


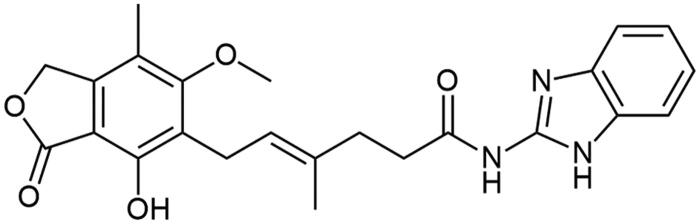
 Method A – 1.5 eq DIPEA, 1.5 eq TBTU, without HOBt·H_2_O, with additional washing step with cold DMF, afforded the product as fluffy, white solid with 43% yield; mp. 253–255 °C (with decomposition); ^1^H NMR (DMSO–d_6_) δ 11.90 (bs, 1H, –NH–), 11.39 (bs, 1H, NH_HetAr_), 9.44 (bs, 1H, Ar–OH), 7.45 − 7.36 (m, 2H, CH_HetAr_), 7.06 (dd, *J* = 5.8, 3.1 Hz, 2H, CH_HetAr_), 5.19 (t, *J* = 6.6 Hz, 1H, >C = CH–), 5.12 (s, 2H, Ar–CH_2_–), 3.63 (s, 3H, Ar–OCH_3_), 3.29 (d, *J* = 6.8 Hz, 2H, Ar–CH_2_–), 2.52 (d, *J* = 6.5 Hz, 2H, –CH_2_–), 2.30 (t, *J* = 7.4 Hz, 2H, –CH_2_–), 1.97 (s, 3H, Ar–CH_3_), 1.79 (s, 3H, –CH_3_); ^13^C NMR (DMSO–d_6_) δ 183.06 (C(2)_HetAr_), 172.40 (–CONH–), 170.60 (–COO–), 162.87 (C_Ar_–OMe), 153.06 (C_Ar_–OH), 146.98 (C_HetAr_), 146.16 (C_Ar_–CH_2_O–), 133.79 (>C = C(–CH_3_)–), 123.62 (C_Ar_–CH_2_–CH=), 122.71 (>C = C(–CH_3_)–), 121.29 (C_HetAr_), 116.29 (C_Ar_–CH_3_), 107.33 (C_Ar_–COO–), 68.96 (Ar–CH_2_O–), 60.97 (Ar–OCH_3_), 34.86 (–CH_2_–CONH–), 34.56 (–CH_2_–CH_2_–), 22.88 (Ar–CH_2_–), 16.48 (=C(–CH_3_)–), 11.40 (Ar–CH_3_); R_T,HPLC_ = 5.617 min, purity 99.50%; HRMS (*m/z*) calculated for C_24_H_25_N_3_O_5_ [M — H]^−^: 434.1716; found: 434.1953.

##### N-(benzo[d]thiazol-6-yl) mycophenolate (A4)


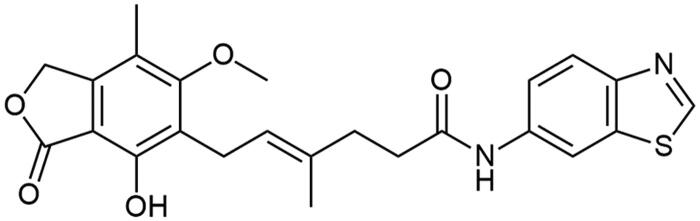
Method B afforded the product as fluffy, creamy solid with 55% yield; mp. 175–177 °C; ^1^H NMR (DMSO–d_6_) δ 10.11 (s, 1H, –NH–), 9.42 (bs, 1H, C(2)H_HetAr_), 9.25 (s, 1H, Ar–OH), 8.47 (d, *J* = 1.7 Hz, 1H, C(7)H_HetAr_), 7.95 (d, *J* = 8.8 Hz, 1H, CH_HetAr_), 7.48 (dd, *J* = 8.8, 1.9 Hz, 1H, CH_HetAr_), 5.23 − 5.15 (m, 3H, >C = CH– and Ar–CH_2_O–), 3.64 (s, 3H, Ar–OCH_3_), 3.30 (d, *J* = 6.8 Hz, 2H, Ar–CH_2_–), 2.41 (t, *J* = 7.6 Hz, 2H, –CH_2_–), 2.28 (t, *J* = 7.5 Hz, 2H, –CH_2_–), 2.00 (s, 3H, Ar–CH_3_), 1.79 (s, 3H, –CH_3_); ^13^C NMR (DMSO–d_6_) δ 171.46 (–CONH–), 170.58 (–COO–), 162.89 (C_Ar_–OMe), 154.92 (C(2)_HetAr_), 153.14 (C_Ar_–OH), 149.31 (C(3a)_HetAr_), 146.13 (C_Ar_–CH_2_O–), 137.42 (C(6)_HetAr_), 134.58 (>C = C(–CH_3_)–), 134.11 (C(7a)_HetAr_), 123.27 (C_Ar_–CH_2_–CH=), 123.24 (C(4)_HetAr_), 122.81 (>C = C(–CH_3_)–), 118.97 (C(5)_HetAr_), 116.30 (C_Ar_–CH_3_), 111.70 (C(7)_HetAr_), 107.34 (C_Ar_–COO–), 69.00 (Ar–CH_2_O–), 60.99 (Ar–OCH_3_), 35.66 (–CH_2_–CONH–), 35.21 (–CH_2_–CH_2_–), 22.91 (Ar–CH_2_–), 16.64 (=C(–CH_3_)–), 11.43 (Ar–CH_3_); R_T,HPLC_ = 8.638 min, purity 100.00%; HRMS (*m/z*) calculated for C_24_H_24_N_2_O_5_S [M — H]^−^: 451.1328; found: 451.1572.

##### N-(benzo[d]thiazol-5-yl) mycophenolate (A5)


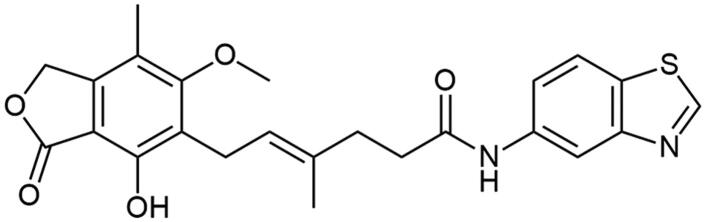
Method B – column chromatography carried out in toluene/AcOEt mixture (2/1, v/v, *R_F_* = 0.18), afforded the product as fluffy, white solid with 67% yield; mp. 114–117 °C; ^1^H NMR (DMSO–d_6_) δ 10.08 (s, 1H, –NH–), 9.26 (m, 2H, Ar–OH and C(2)H_HetAr_), 8.38 (d, *J* = 1.8 Hz, 1H, CH_HetAr_), 8.01 (d, *J* = 8.7 Hz, 1H, CH_HetAr_), 7.50 (dd, *J* = 8.7, 1.9 Hz, 1H, CH_HetAr_), 5.22 − 5.16 (m, 3H, >C = CH– and Ar–CH_2_O–), 3.64 (s, 3H, Ar–OCH_3_), 3.30 (d, *J* = 6.9 Hz, 2H, Ar–CH_2_–), 2.41 (dd, *J* = 8.9, 6.4 Hz, 2H, –CH_2_–), 2.28 (t, *J* = 7.6 Hz, 2H, –CH_2_–), 2.00 (s, 3H, Ar–CH_3_), 1.79 (s, 3H, –CH_3_); ^13^C NMR (DMSO–d_6_) δ 171.44 (–CONH–), 170.60 (–COO–), 162.89 (C_Ar_–OMe), 157.24 (C(2)_HetAr_), 153.98 (C(3a)_HetAr_), 153.12 (C_Ar_–OH), 146.15 (C_Ar_–CH_2_O–), 138.29 (C(5)_HetAr_), 134.14 (>C = C(–CH_3_)–), 127.98 (C(7a)_HetAr_), 123.26 (C_Ar_–CH_2_–CH=), 122.82 (C(6)_HetAr_), 122.57 (>C = C(–CH_3_)–), 118.33 (C(7)_HetAr_), 116.32 (C_Ar_–CH_3_), 112.98 (C(4)_HetAr_), 107.34 (C_Ar_–COO–), 69.02 (Ar–CH_2_O–), 60.98 (Ar–OCH_3_), 35.70 (–CH_2_–CONH–), 35.17 (–CH_2_–CH_2_–), 22.90 (Ar–CH_2_–), 16.66 (=C(–CH_3_)–), 11.41 (Ar–CH_3_); R_T,HPLC_ = 8.651 min, purity 100.00%; HRMS (*m/z*) calculated for C_24_H_24_N_2_O_5_S [M — H]^−^: 451.1328; found: 451.1573.

##### N-(benzo[d]oxazol-5-yl) mycophenolate (A6)


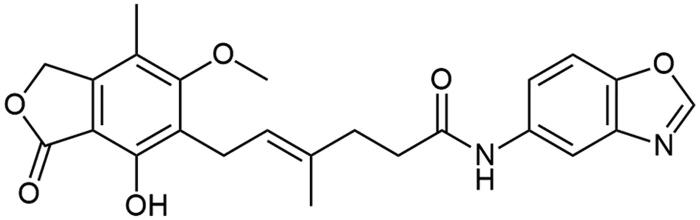
Method B – column chromatography carried out in toluene/acetone mixture (3/1, v/v, *R_F_* = 0.34), afforded the product as beige solid with 53% yield; mp. 84–87 °C; ^1^H NMR (DMSO–d_6_) δ 10.01 (s, 1H, –NH–), 9.37 (s, 1H, Ar–OH), 8.69 (s, 1H, C(2)H_HetAr_), 8.05 (d, *J* = 1.9 Hz, 1H, C(4)H_HetAr_), 7.64 (d, *J* = 8.8 Hz, 1H, CH_HetAr_), 7.43 (dd, *J* = 8.8, 2.0 Hz, 1H, CH_HetAr_), 5.19 (m, 3H, >C = CH– and Ar–CH_2_O–), 3.64 (s, 3H, Ar–OCH_3_), 3.30 (d, *J* = 6.8 Hz, 2H, Ar–CH_2_–), 2.45 − 2.35 (m, 2H, –CH_2_–), 2.28 (dd, *J* = 13.4, 5.3 Hz, 2H, –CH_2_–), 2.01 (s, 3H, Ar–CH_3_), 1.79 (s, 3H, –CH_3_); ^13^C NMR (DMSO–d_6_) δ 171.21 (–CONH–), 170.59 (–COO–), 162.91 (C_Ar_–OMe), 155.22 (C(2)_HetAr_), 153.13 (C_Ar_–OH), 146.16 (C_Ar_–CH_2_O–), 145.68 (C(3a)_HetAr_), 140.19 (C(7a)_HerAr_), 136.73 (C(5)_HetAr_), 134.16 (>C = C(–CH_3_)–), 123.23 (C_Ar_–CH_2_–CH=), 122.83 (>C = C(–CH_3_)–), 118.00 (C(6)_HetAr_), 116.33 (C_Ar_–CH_3_), 111.18 (C_HetAr_), 110.54 (C_HetAr_), 107.35 (C_Ar_–COO–), 69.03 (Ar–CH_2_O–), 60.98 (Ar–OCH_3_), 35.62 (–CH_2_–CONH–), 35.20 (–CH_2_–CH_2_–), 22.90 (Ar–CH_2_–), 16.64 (=C(–CH_3_)–), 11.43 (Ar–CH_3_); R_T,HPLC_ = 7.866 min, purity 95.79%; HRMS (*m/z*) calculated for C_24_H_24_N_2_O_6_ [M — H]^−^: 435.1556; found: 435.1795.

##### N-[1-(pirymidin-2-yl)methyl] mycophenolate (A7)


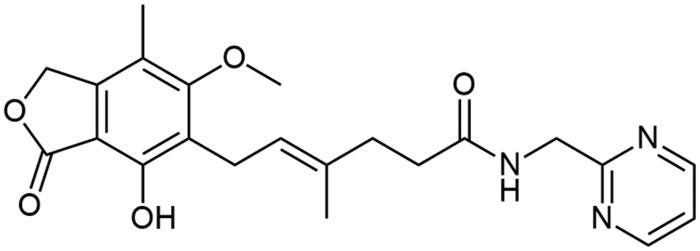
Method B – column chromatography carried out in toluene/acetone mixture (2/1, v/v, *R_F_* = 0.13), afforded the product as white solid with 61% yield; mp. 107–110 °C; ^1^H NMR (DMSO–d_6_) δ 9.41 (s, 1H, Ar–OH), 8.73 (d, *J* = 4.9 Hz, 2H, CH_HetAr_), 8.33 (t, *J* = 5.8 Hz, 1H, –NH–), 7.37 (t, *J* = 4.9 Hz, 1H, CH_HetAr_), 5.24 (s, 2H, Ar–CH_2_O–), 5.14 (t, *J* = 6.9 Hz, 1H, >C = CH–), 4.40 (d, *J* = 5.8 Hz, 2H, –CH_2_–HetAr), 3.69 (s, 3H, Ar–OCH_3_), 3.30 (d, *J* = 8.3 Hz, 2H, Ar–CH_2_–), 2.28 − 2.21 (m, 2H, –CH_2_–), 2.17 (dd, *J* = 8.8, 5.6 Hz, 2H, –CH_2_–), 2.08 (s, 3H, Ar–CH_3_), 1.75 (s, 3H, –CH_3_); ^13^C NMR (DMSO–d_6_) δ 172.34 (–CONH–), 170.59 (–COO–), 167.48 (C(2)_HetAr_), 163.00 (C_Ar_–OMe), 157.72 (C(4)_HetAr_ and C(6)_HetAr_), 153.17 (C_Ar_–OH), 146.23 (C_Ar_–CH_2_O–), 134.52 (>C = C(–CH_3_)–), 123.01 (C_Ar_–CH_2_–CH=), 122.94 (>C = C(–CH_3_)–), 120.17 (C(5)_HetAr_), 116.39 (C_Ar_–CH_3_), 107.41 (C_Ar_–COO–), 69.06 (Ar–CH_2_O–), 61.07 (Ar–OCH_3_), 45.22 (–CONH–CH_2_–HetAr), 35.45 (–CH_2_–CONH–), 34.56 (–CH_2_–CH_2_–), 22.88 (Ar–CH_2_–), 16.49 (=C(–CH_3_)–), 11.51 (Ar–CH_3_); R_T,HPLC_ = 5.293 min, purity 99.19%; HRMS (*m/z*) calculated for C_22_H_25_N_3_O_5_ [M — H]^−^: 410.1716; found: 410.1936.

##### N-(6-methoxybenzo[d]thiazol-2-yl) mycophenolate (A8)


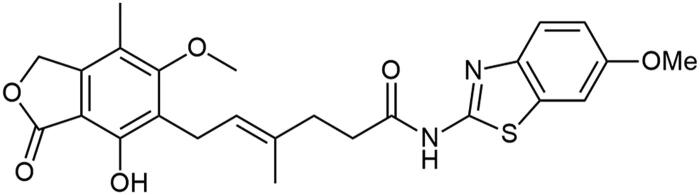
Method A – 2.0 eq DIPEA, afforded the product as white solid with 100% yield; mp. 246–248 °C (with decomposition); ^1^H NMR (DMSO–d_6_) δ 12.10 (s, 1H, –NH–), 9.35 (s, 1H, Ar–OH), 7.60 (d, *J* = 8.8 Hz, 1H, CH_HetAr_), 7.53 (d, *J* = 2.4 Hz, 1H, CH_HetAr_), 7.02 (dd, *J* = 8.8, 2.5 Hz, 1H, CH_HetAr_), 5.17 (t, *J* = 6.7 Hz, 1H, >C = CH–), 5.08 (s, 2H, Ar–CH_2_O–), 3.81 (s, 3H, HetAr–OCH_3_), 3.65 (s, 3H, Ar–OCH_3_), 3.28 (d, *J* = 6.8 Hz, 2H, Ar–CH_2_–), 2.58 − 2.52 (m, 2H, –CH_2_–), 2.28 (t, *J* = 7.4 Hz, 2H, –CH_2_–), 1.98 (s, 3H, Ar–CH_3_), 1.78 (s, 3H, –CH_3_); ^13^C NMR (DMSO–d_6_) δ 171.89 (–CONH–), 170.61 (–COO–), 162.94 (C_Ar_–OMe), 156.51 (C(2)_HetAr_), 156.28 (C(6)_HetAr_), 153.11 (C_Ar_–OH), 146.06 (C_Ar_–CH_2_O–), 143.07 (C(3a)_HetAr_), 133.72 (>C = C(–CH_3_)–), 133.21 (C(7a)_HetAr_), 123.61 (C_Ar_–CH_2_–CH=), 122.65 (>C = C(–CH_3_)–), 121.44 (C(4)_HetAr_), 116.30 (C_Ar_–CH_3_), 115.19 (C(5)_HetAr_), 107.29 (C_Ar_–COO–), 105.13 (C(7)_HetAr_), 68.95 (Ar–CH_2_O–), 61.01 (Ar–OCH_3_), 56.12 (HetAr–OCH_3_), 34.69 (–CH_2_–CONH–), 34.35 (–CH_2_–CH_2_–), 22.89 (Ar–CH_2_–), 16.50 (=C(–CH_3_)–), 11.42 (Ar–CH_3_); R_T,HPLC_ = 12.073 min, purity 99.43%; HRMS (*m/z*) calculated for C_25_H_26_N_2_O_6_S [M — H]^−^: 481.1433; found: 481.1692.

##### N-(6-methylbenzo[d]thiazol-2-yl) mycophenolate (A9)


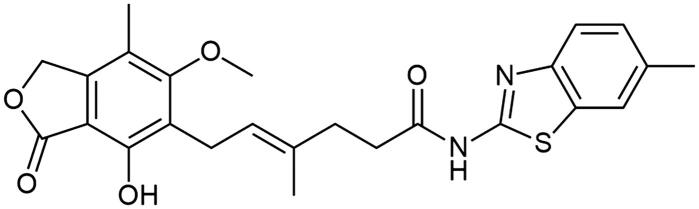
Method A – 1.0 eq DIPEA, afforded the product as white solid with 81% yield; mp. 257–260 °C (with decomposition); ^1^H NMR (DMSO–d_6_) δ 12.15 (s, 1H, –NH–), 9.38 (s, 1H, Ar–OH), 7.72 (s, 1H, C(7)H_HetAr_), 7.59 (d, *J* = 8.2 Hz, 1H, CH_HetAr_), 7.24 (d, *J* = 8.2 Hz, 1H, CH_HetAr_), 5.17 (t, *J* = 6.7 Hz, 1H, >C = CH–), 5.06 (s, 2H, Ar–CH_2_O–), 3.67 (s, 3H, Ar–OCH_3_), 3.28 (d, *J* = 6.9 Hz, 2H, Ar–CH_2_–), 2.53 (m, 2H, –CH_2_–), 2.41 (s, 3H, HetAr–CH_3_), 2.27 (t, *J* = 7.4 Hz, 2H), 1.97 (s, 3H, Ar–CH_3_), 1.76 (s, 3H, –CH_3_); ^13^C NMR (DMSO–d_6_) δ 172.03 (–CONH–), 170.67 (–COO–), 163.03 (C_Ar_–OMe), 157.42 (C(2)_HetAr_), 153.14 (C_Ar_–OH), 146.97 (C(3a)_HetAr_), 146.02 (C_Ar_–CH_2_O–), 133.73 (>C = C(–CH_3_)–), 133.25 (C(7a)_HetAr_), 132.12 (C(6)_HetAr_), 127.71 (C(5)_HetAr_), 123.64 (C_Ar_–CH_2_–CH=), 122.63 (>C = C(–CH_3_)–), 121.55 (C_HetAr_), 120.50 (C_HetAr_), 116.33 (C_Ar_–CH_3_), 107.30 (C_Ar_–COO–), 68.99 (Ar–CH_2_O–), 61.03 (Ar–OCH_3_), 34.69 (–CH_2_–CONH–), 34.46 (–CH_2_–CH_2_–), 22.89 (Ar–CH_2_–), 21.39 (HetAr–CH_3_), 16.48 (=C(–CH_3_)–), 11.41 (Ar–CH_3_); R_T,HPLC_ = 13.755 min, purity 98.99%; HRMS (*m/z*) calculated for C_25_H_26_N_2_O_5_S [M — H]^−^: 465.1484; found: 465.1721.

##### N-(5,6-dimethylbenzo[d]thiazol-2-yl) mycophenolate (A10)


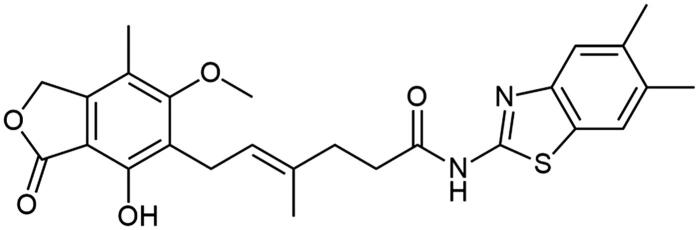
Method A – 1.0 eq DIPEA, afforded the product as white solid with 77% yield; mp. 246–247 °C (with decomposition); ^1^H NMR (DMSO–d_6_) δ 12.12 (s, 1H, –NH–), 9.36 (s, 1H, Ar–OH), 7.66 (s, 1H, CH_HetAr_), 7.50 (s, 1H, CH_HetAr_), 5.17 (t, *J* = 6.6 Hz, 1H, >C = CH–), 5.08 (s, 2H, Ar–CH_2_O–), 3.64 (s, 3H, Ar–OCH_3_), 3.28 (d, *J* = 6.7 Hz, 2H, Ar–CH_2_–), 2.57 − 2.51 (m, 2H, –CH_2_–), 2.33 (m, 8H, –CH_2_–, HetAr(5)–CH_3_, and HetAr(6)–CH_3_), 1.96 (s, 3H, Ar–CH_3_), 1.78 (s, 3H, –CH_3_); ^13^C NMR (DMSO–d_6_) δ 171.93 (–CONH–), 170.57 (–COO–), 162.86 (C_Ar_–OMe), 157.33 (C(2)_HetAr_), 153.08 (C_Ar_–OH), 147.48 (C(3a)_HetAr_), 146.13 (C_Ar_–CH_2_O–), 135.06 (C(7a)_HetAr_), 133.70 (>C = C(–CH_3_)–), 132.64 (C(6)_HetAr_), 129.22 (C(5)_HetAr_), 123.59 (C_Ar_–CH_2_–CH=), 122.67 (>C = C(–CH_3_)–), 121.77 (C(7)_HetAr_), 121.24 (C(4)_HetAr_), 116.26 (C_Ar_–CH_3_), 107.30 (C_Ar_–COO–), 68.90 (Ar–CH_2_O–), 60.99 (Ar–OCH_3_), 34.68 (–CH_2_–CONH–), 34.32 (–CH_2_–CH_2_–), 22.88 (Ar–CH_2_–), 20.17 (HetAr–CH_3_), 20.01 (HetAr–CH_3_), 16.50 (=C(–CH_3_)–), 11.42 (Ar–CH_3_); R_T,HPLC_ = 14.909 min, purity 97.49%; HRMS (*m/z*) calculated for C_26_H_28_N_2_O_5_S [M — H]^−^: 479.1641; found: 479.1893.

##### N-(6-fluorobenzo[d]thiazol-2-yl) mycophenolate (A11)


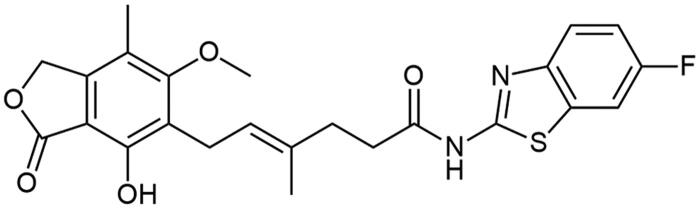
Method A – 2.0 eq DIPEA, afforded the product as white solid with 71% yield; mp. 232–236 °C (with decomposition); ^1^H NMR (DMSO–d_6_) δ 12.26 (s, 1H, –NH–), 9.35 (s, 1H, Ar–OH), 7.86 (dd, *J* = 8.7, 2.5 Hz, 1H, CH_HetAr_), 7.71 (dd, *J* = 8.8, 4.8 Hz, 1H, CH_HetAr_), 7.28 (td, *J* = 9.1, 2.6 Hz, 1H, CH_HetAr_), 5.17 (t, *J* = 6.7 Hz, 1H, >C = CH–), 5.08 (s, 2H, Ar–CH_2_O–), 3.65 (s, 3H, Ar–OCH_3_), 3.28 (d, *J* = 6.8 Hz, 2H, Ar–CH_2_–,), 2.55 (t, *J* = 7.5 Hz, 2H, –CH_2_–), 2.29 (t, *J* = 7.4 Hz, 2H, –CH_2_–), 1.98 (s, 3H, Ar–CH_3_), 1.78 (s, 3H, –CH_3_); ^13^C NMR (DMSO–d_6_) δ 172.26 (–CONH–), 170.66 (–COO–), 163.04 (C_Ar_–OMe), 160.02 (C(2)_HetAr_), 158.20 (d, J = 21.3 Hz, C(6)_HetAr_), 153.14 (C_Ar_–OH), 145.98 (C_Ar_–CH_2_O–), 145.71 (C(3a)_HetAr_), 133.70 (>C = C(–CH_3_)–), 133.20 (d, J = 11.1 Hz, C(7a)_HetAr_), 123.65 (C_Ar_–CH_2_–CH=), 122.63 (>C = C(–CH_3_)–), 121.90 (d, *J* = 9.2 Hz, C(4)_HetAr_), 116.34 (C_Ar_–CH_3_), 114.45 (d, *J* = 24.5 Hz, C(5)_HetAr_)), 108.37 (d, *J* = 26.8 Hz, C(7)_HetAr_), 107.28 (C_Ar_–COO–), 69.02 (Ar–CH_2_O–), 61.02 (Ar–OCH_3_), 34.63 (–CH_2_–CONH–), 34.43 (–CH_2_–CH_2_–), 22.89 (Ar–CH_2_–), 16.47 (=C(–CH_3_)–), 11.40 (Ar–CH_3_); R_T,HPLC_ = 12.970 min, purity 99.58%; HRMS (*m/z*) calculated for C_24_H_23_FN_2_O_5_S [M — H]^−^: 469.1233; found: 469.1488.

##### N-(6-chlorobenzo[d]thiazol-2-yl) mycophenolate (A12)


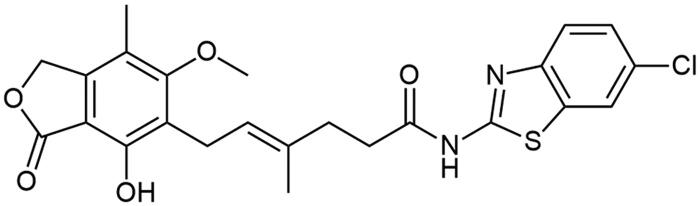
Method A – 1.0 eq DIPEA, afforded the product as white solid with 78% yield; mp. 261–264 °C (with decomposition); ^1^H NMR (DMSO–d_6_) δ 12.33 (s, 1H, –NH–), 9.38 (s, 1H, Ar–OH), 8.09 (s, 1H, C(7)H_HetAr_), 7.70 (d, *J* = 8.6 Hz, 1H, CH_HetAr_), 7.45 (d, *J* = 8.5 Hz, 1H, CH_HetAr_), 5.17 (t, *J* = 6.2 Hz, 1H, >C = CH–), 5.08 (s, 2H, Ar–CH_2_O–), 3.67 (s, 3H, Ar–OCH_3_), 3.28 (d, *J* = 6.6 Hz, 2H, Ar–CH_2_–), 2.56 (t, *J* = 7.4 Hz, 2H, –CH_2_–), 2.29 (t, *J* = 7.1 Hz, 2H, –CH_2_–), 1.97 (s, 3H, Ar–CH_3_), 1.78 (s, 3H,–CH_3_); ^13^C NMR (DMSO–d_6_) δ 172.40 (–CONH–), 170.61 (–COO–), 162.96 (C_Ar_–OMe), 159.12 (C(2)_HetAr_), 153.12 (C_Ar_–OH), 147.86 (C(3a)_HetAr_), 146.02 (C_Ar_–CH_2_O–), 133.64 (>C = C(–CH_3_)–), 127.90 (C_HetAr_), 126.77 (C_HetAr_), 123.67 (C_Ar_–CH_2_–CH=), 122.64 (>C = C(–CH_3_)–), 122.04 (C_HetAr_), 121.69 (C_HetA_r), 116.30 (C_Ar_–CH_3_), 107.28 (C_Ar_–COO–), 68.96 (Ar–CH_2_O–), 61.01 (Ar–OCH_3_), 34.62 (–CH_2_–CONH–), 34.41 (–CH_2_–CH_2_–), 22.89 (Ar–CH_2_–), 16.48 (=C(–CH_3_)–), 11.41 (Ar–CH_3_); R_T,HPLC_ = 14.845 min, purity 97.55%; HRMS (*m/z*) calculated for C_24_H_23_ClN_2_O_5_S [M — H]^−^: 485.0938; found: 485.1186.

##### N-(6-bromobenzo[d]thiazol-2-yl) mycophenolate (A13)


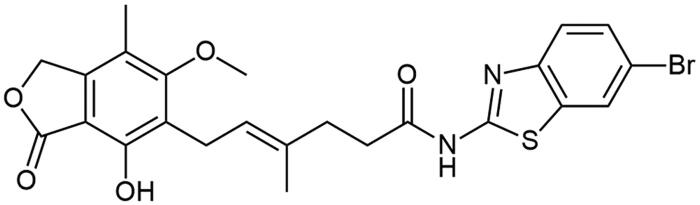
Method A – 1.0 eq DIPEA, additional 0.15 mL of dry 1,4-D was added to enhance solubility, afforded the product as creamy solid with 80% yield; mp. 265–267 °C (with decomposition); ^1^H NMR (DMSO–d_6_) δ 12.33 (s, 1H, –NH–), 9.37 (s, 1H, Ar–OH), 8.22 (d, *J* = 1.2 Hz, 1H, CH_HetAr_), 7.67 (d, *J* = 8.6 Hz, 1H, CH_HetAr_), 7.57 (dd, *J* = 8.6, 1.4 Hz, 1H, CH_HetAr_), 5.17 (t, *J* = 6.6 Hz, 1H, >C = CH–), 5.08 (s, 2H, Ar–CH_2_O–), 3.64 (s, 3H, Ar–OCH_3_), 3.28 (d, *J* = 6.8 Hz, 2H, Ar–CH_2_–), 2.56 (t, *J* = 7.4 Hz, 2H, –CH_2_–), 2.29 (t, *J* = 7.4 Hz, 2H, –CH_2_–), 1.97 (s, 3H, Ar–CH_3_), 1.76 (s, 3H, –CH_3_); ^13^C NMR (DMSO–d_6_) δ 172.41 (–CONH–), 170.65 (–COO–), 163.02 (C_Ar_–OMe), 159.10 (C(2)_HetAr_), 153.14 (C_Ar_–OH), 148.18 (C(3a)_HetAr_), 145.99 (C_Ar_–CH_2_O–), 134.18 (C(7a)_HetAr_), 133.66 (>C = C(–CH_3_)–), 129.45 (C(7a)_HetAr_), 124.50 (C_HetAr_), 123.68 (C_Ar_–CH_2_–CH=), 122.62 (>C = C(–CH_3_)–), 122.45 (C_HetAr_), 116.33 (C_Ar_–CH_3_), 115.73 (C(6)_HetAr_), 107.29 (C_Ar_–COO–), 68.99 (Ar–CH_2_O–), 61.02 (Ar–OCH_3_), 34.63 (–CH_2_–CONH–), 34.46 (–CH_2_–CH_2_–), 22.89 (Ar–CH_2_–), 16.47 (=C(–CH_3_)–), 11.40 (Ar–CH_3_); R_T,HPLC_ = 15.319 min, purity 97.88%; HRMS (*m/z*) calculated for C_24_H_23_BrN_2_O_5_S [M — H]^−^: 529.0433, 531.0415; found: 529.0703, 531.0687.

##### N-[6-(trifluoromethyl)benzo[d]thiazol-2-yl] mycophenolate (A14)


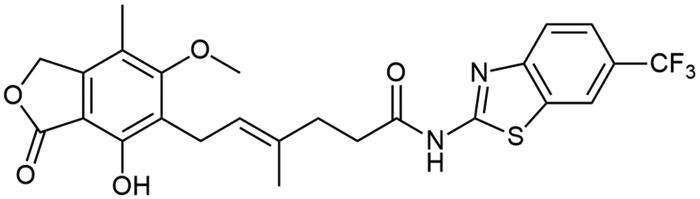
Method A – 2.0 eq DIPEA, afforded the product as white solid with 65% yield; mp. 261–264 °C (with decomposition); ^1^H NMR (DMSO–d_6_) δ 12.49 (s, 1H, –NH), 9.34 (s, 1H, Ar–OH), 8.47 (s, 1H, C(7)H_HetAr_), 7.87 (d, *J* = 8.5 Hz, 1H, CH_HetAr_), 7.74 (d, *J* = 8.5 Hz, 1H, CH_HetAr_), 5.18 (t, *J* = 6.8 Hz, 1H, >C = CH–), 5.05 (s, 2H, Ar–CH_2_O–), 3.64 (s, 3H, Ar–OCH_3_), 3.28 (d, *J* = 6.9 Hz, 2H, Ar–CH_2_–), 2.59 (t, *J* = 7.5 Hz, 2H, –CH_2_–), 2.37 − 2.24 (m, 2H, –CH_2_–), 1.96 (s, 3H, Ar–CH_3_), 1.79 (s, 3H, –CH_3_); ^13^C NMR (DMSO–d_6_) δ 172.66 (–CONH–), 170.59 (–COO–), 162.96 (C_Ar_–OMe), 161.47 (C(2)_HetAr_), 153.11 (C_Ar_–OH), 151.74 (C(3a)_HetAr_), 145.99 (C_Ar_–CH_2_O–), 133.60 (>C = C(–CH_3_)–), 132.50 (C(7a)_HetAr_), 126.14 (C_HetAr_), 124.09 (d, *J* = 31.9 Hz, HetAr–CF_3_) 123.70 (C_Ar_–CH_2_–CH=), 123.24 (d, *J* = 3.4 Hz, C_HetAr_), 122.63 (>C = C(–CH_3_)–), 121.24 (C_HetAr_), 120.14 (d, J = 4.1 Hz, C(4)_HetAr_), 116.28 (C_Ar_–CH_3_), 107.27 (C_Ar_–COO–), 68.91 (Ar–CH_2_O–), 61.00 (Ar–OCH_3_), 34.57 (–CH_2_–CONH–), 34.44 (–CH_2_–CH_2_–), 22.89 (Ar–CH_2_–), 16.48 (=C(–CH_3_)–), 11.37 (Ar–CH_3_); R_T,HPLC_ = 15.435 min, purity 97.28%; HRMS (*m/z*) calculated for C_25_H_23_F_3_N_2_O_5_S [M — H]^−^: 519.1202; found: 519.1484.

##### N-(6-nitrobenzo[d]thiazol-2-yl) mycophenolate (A15)


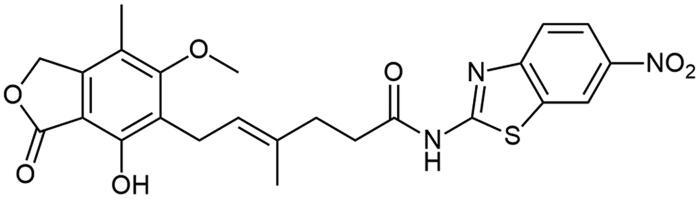
Method A – 1.0 eq DIPEA, reaction was carried out in 0.6 mL of dry DMF and 1.0 mL of dry ACN, afforded the product as beige solid with 42% yield; mp. 278–280 °C (with decomposition); ^1^H NMR (DMSO–d_6_) δ 12.66 (s, 1H, –NH–), 9.37 (s, 1H, Ar–OH), 9.03 (d, *J* = 2.0 Hz, 1H, CH_HetAr_), 8.28 (dd, *J* = 8.0, 4.0 Hz, 1H, CH_HetAr_), 7.86 (d, *J* = 8.9 Hz, 1H, CH_HetAr_), 5.18 (t, *J* = 6.6 Hz, 1H, >C = CH–), 5.08 (s, 2H, Ar–CH_2_O–), 3.65 (s, 3H, Ar–OCH_3_), 3.28 (d, *J* = 6.7 Hz, 2H, Ar–CH_2_–), 2.61 (t, *J* = 7.4 Hz, 2H, –CH_2_–), 2.31 (t, *J* = 7.3 Hz, 2H, –CH_2_–), 1.97 (s, 3H, Ar–CH_3_), 1.80 (s, 3H, –CH_3_); ^13^C NMR (DMSO–d_6_) δ 172.96 (–CONH–), 170.57 (–COO–), 163.82 (C(2)_HetAr_), 162.98 (C_Ar_–OMe), 153.92 (C(6)_HetAr_), 153.12 (C_Ar_–OH), 146.02 (C_Ar_–CH_2_O–), 143.41 (C(3a)_HetAr_), 133.58 (>C = C(–CH_3_)–), 132.66 (C(7a)_HetAr_), 123.73 (C_Ar_–CH_2_–CH=), 122.63 (>C = C(–CH_3_)–), 122.12 (C(5)_HetAr_), 120.90 (C(4)_HetAr_), 119.28 (C(7)_HetAr_), 116.31 (C_Ar_–CH_3_), 107.28 (C_Ar_–COO–), 68.99 (Ar–CH_2_O–), 61.02 (Ar–OCH_3_), 34.52 (–CH_2_–CONH–), 34.50 (–CH_2_–CH_2_–), 22.89 (Ar–CH_2_–), 16.49 (=C(–CH_3_)–), 11.41 (Ar–CH_3_); R_T,HPLC_ = 13.238 min, purity 97.60%; HRMS (*m/z*) calculated for C_24_H_23_N_3_O_7_S [M — H]^−^: 496.1178; found: 496.1442.

##### N-(4-methoxybenzo[d]thiazol-2-yl) mycophenolate (A16)


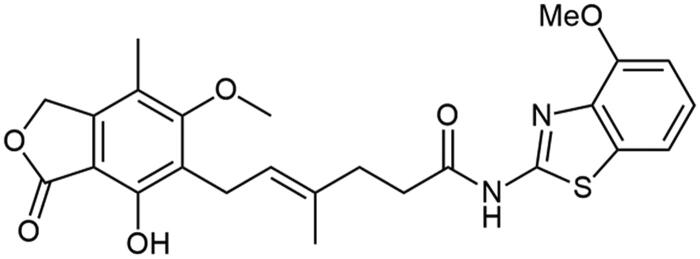
Method A – 1.0 eq DIPEA, washing step limited to cold ACN usage, afforded the product as white solid with 66% yield; mp. 165–168 °C; ^1^H NMR (DMSO–d_6_) δ 12.35 (s, 1H, –NH–), 9.34 (s, 1H, Ar–OH), 7.47 (d, *J* = 7.9 Hz, 1H, CH_HetAr_), 7.24 (t, *J* = 8.0 Hz, 1H, C(6)H washing step limited to cold ACN usage _HetAr_), 6.99 (d, *J* = 8.0 Hz, 1H, CH_HetAr_), 5.17 (t, *J* = 6.7 Hz, 1H, >C = CH–), 5.03 (s, 2H, Ar–CH_2_O–), 3.91 (s, 3H, HetAr–OCH_3_), 3.64 (s, 3H, Ar–OCH_3_), 3.28 (d, *J* = 6.9 Hz, 2H, Ar–CH_2_–), 2.59 − 2.51 (m, 2H, –CH_2_–), 2.29 (t, *J* = 7.4 Hz, 2H, –CH_2_–), 1.95 (s, 3H, Ar–CH_3_), 1.78 (s, 3H, –CH_3_); ^13^C NMR (DMSO–d_6_) δ 171.92 (–CONH–), 170.67 (–COO–), 163.01 (C_Ar_–OMe), 156.70 (C(2)_HetAr_), 153.11 (C_Ar_–OH), 152.35 (C(4)_HetAr_), 145.98 (C_Ar_–CH_2_O–), 138.94 (C(3a)_HetAr_), 133.66 (>C = C(–CH_3_)–), 133.38 (C(7a)_HetAr_), 124.73 (C(6)_HetAr_), 123.71 (C_Ar_–CH_2_–CH=), 122.59 (>C = C(–CH_3_)–), 116.31 (C_Ar_–CH_3_), 113.85 (C(7)_HetAr_), 108.31 (C(5)_HetAr_), 107.27 (C_Ar_–COO–), 69.00 (Ar–CH_2_O–), 61.02 (Ar–OCH_3_), 56.38 (HetAr–OCH_3_), 34.70 (–CH_2_–CONH–), 34.46 (–CH_2_–CH_2_–), 22.88 (Ar–CH_2_–), 16.44 (=C(–CH_3_)–), 11.35 (Ar–CH_3_); R_T,HPLC_ = 12.274 min, purity 98.12%; HRMS (*m/z*) calculated for C_25_H_26_N_2_O_6_S [M — H]^−^: 481.1433; found: 481.1695.

##### N-(4-methylbenzo[d]thiazol-2-yl) mycophenolate (A17)


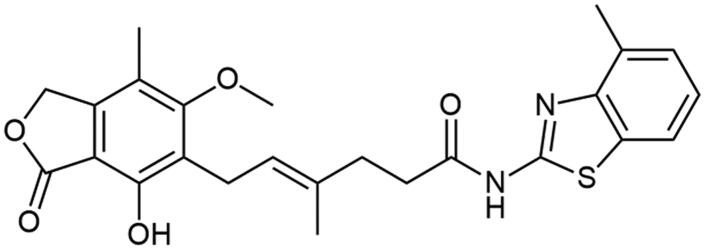
Method A – 1.0 eq DIPEA, washing step limited to cold ACN usage, afforded the product as white solid with 67% yield; mp. 173–176 °C; ^1^H NMR (DMSO–d_6_) δ 12.32 (s, 1H, –NH–), 9.31 (s, 1H, Ar–OH), 7.74 (d, *J* = 7.7 Hz, 1H, CH_HetAr_), 7.25 (d, *J* = 7.2 Hz, 1H, CH_HetAr_), 7.20 (t, *J* = 7.5 Hz, 1H, C(6)H_HetAr_), 5.18 (t, *J* = 6.8 Hz, 1H, >C = CH–), 4.95 (s, 2H, Ar–CH_2_O–), 3.65 (s, 3H, Ar–OCH_3_), 3.27 (d, *J* = 6.9 Hz, 2H, Ar–CH_2_–), 2.60 − 2.52 (m, 5H, –CH_2_– and HetAr–CH_3_), 2.29 (t, *J* = 7.3 Hz, 2H, –CH_2_–), 1.93 (s, 3H, Ar–CH_3_), 1.78 (s, 3H, –CH_3_); ^13^C NMR (DMSO–d_6_) δ 172.11 (–CONH–), 170.56 (–COO–), 168.41, 162.78 (C_Ar_–OMe), 157.39 (C(2)_HetAr_), 153.01 (C_Ar_–OH), 147.95 (C(3a)_HetAr_), 145.99 (C_Ar_–CH_2_O–), 133.55 (>C = C(–CH_3_)–), 131.52 (C(7a)_HetAr_), 130.17 (C_HetAr_), 126.93 (C_HetAr_), 123.75 (C_HetAr_), 123.67 (C_Ar_–CH_2_–CH=), 122.61 (>C = C(–CH_3_)–), 119.38 (C(7)_HetAr_), 116.16 (C_Ar_–CH_3_), 107.19 (C_Ar_–COO–), 68.83 (Ar–CH_2_O–), 60.96 (Ar–OCH_3_), 34.72 (–CH_2_–CONH–), 34.24 (–CH_2_–CH_2_–), 22.86 (Ar–CH_2_–), 18.44 (HetAr–CH_3_), 16.49 (=C(–CH_3_)–), 11.35 (Ar–CH_3_); R_T,HPLC_ = 14.096 min, purity 100.00%; HRMS (*m/z*) calculated for C_25_H_26_N_2_O_5_S [M — H]^−^: 465.1484; found: 465.1731.

##### N-(4-chlorobenzo[d]thiazol-2-yl) mycophenolate (A18)


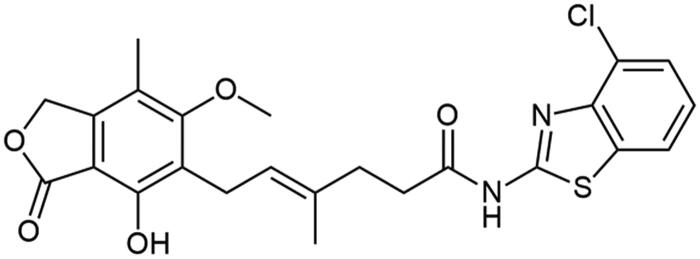
Method A – 1.0 eq DIPEA, afforded the product as chalky solid with 76% yield; mp. 223–227 °C; ^1^H NMR (DMSO–d_6_) δ 12.64 (s, 1H, –NH–), 9.33 (s, 1H, Ar–OH), 7.93 (d, *J* = 7.9 Hz, 1H, CH_HetAr_), 7.52 (d, *J* = 7.7 Hz, 1H, CH_HetAr_), 7.30 (t, *J* = 7.9 Hz, 1H, C(6)H_HetAr_), 5.18 (t, *J* = 6.8 Hz, 1H, >C = CH–), 5.01 (s, 2H, Ar–CH_2_O–), 3.64 (s, 3H, Ar–OCH_3_), 3.28 (d, *J* = 6.9 Hz, 2H, Ar–CH_2_–), 2.62 − 2.53 (m, 2H, –CH_2_–), 2.29 (t, *J* = 7.3 Hz, 2H, –CH_2_–), 1.94 (s, 3H, Ar–CH_3_), 1.78 (s, 3H, –CH_3_); ^13^C NMR (DMSO–d_6_) δ 172.48 (–CONH–), 170.53 (–COO–), 162.79 (C_Ar_–OMe), 159.34 (C(2)_HetAr_), 153.05 (C_Ar_–OH), 145.99 (C_Ar_–CH_2_O–), 145.77 (C(3a)_HetAr_), 133.48 (>C = C(–CH_3_)–), 126.55 (C_HetAr_), 124.70 (C_HetAr_), 123.76 (C_Ar_–CH_2_–CH=), 122.60 (>C = C(–CH_3_)–), 121.17 (C_HetAr_), 116.19 (C_Ar_–CH_3_), 107.21 (C_Ar_–COO–), 68.85 (Ar–CH_2_O–), 60.95 (Ar–OCH_3_), 34.68 (–CH_2_–CONH–), 34.27 (–CH_2_–CH_2_–), 22.87 (Ar–CH_2_–), 16.44 (=C(–CH_3_)–), 11.33 (Ar–CH_3_); R_T,HPLC_ = 13.969 min, purity 97.67%; HRMS (*m/z*) calculated for C_24_H_23_ClN_2_O_5_S [M — H]^−^: 485.0938; found: 485.1194.

### Biological evaluation

#### Research materials

T-Jurkat (Clone E6-1, ATCC TIB-152), a human T lymphoblastoid cell line from an acute T-cell leukaemia was received from a cell bank of Department of Medical Immunology, Medical University of Gdansk. Prior to testing, cells were thawed and passaged for approximately 7 days in order to enter the logarithmic growth phase.

Human peripheral blood mononuclear cells (PBMCs) were obtained from buffy coats of anonymous, healthy volunteers, with the average age 33 ± 6 years, from the Regional Centre for Blood Donation and Treatment in Gdansk.

#### Growing medium

T-Jurkat cell line was suspended in a complete RPMI medium containing RPMI-1640 media (Sigma Aldrich, Poznań, Poland), 10% FBS (Sigma Aldrich, Poznań, Poland), penicillin (p, 100 U/mL) and streptomycin (s, 100 mg/mL, Sigma Aldrich, Poznań, Poland). PBMCs were suspended in a complete X-VIVO medium containing X-VIVO20 media (X-VIVO 20, Lonza, Verviers, Belgium), 10% heat-inactivated human AB serum (National Blood Bank, Raciborz, Poland), penicillin (p, 100 U/mL) and streptomycin (s, 100 mg/mL).

#### MPA derivatives preparation

To prepare appropriate concentrations of tested compounds, the substances were dissolved in DMSO (Sigma Aldrich, Poland) with 1% addition of Tween20 to obtain final concentration of 0.01 M. The solution was diluted 10-fold and then 5-fold in appropriate medium without additives (RPMI-1640 for T-Jurkat cell line; X-VIVO20 for PBMCs) to obtain the following concentrations: 200; 20; 2; 0.2; 0.02 µM.

#### PBMCS isolation

PBMCs were isolated by gradient centrifugation, as described previously^52^. Buffy coats were diluted with phosphate-buffered saline (PBS, pH 7.4, ThermoFisher Scientific, Waltham, MA) in a 1:1 ratio, layered at Ficoll-Paque^™^ PLUS (VWR International, Gdańsk, Poland) and centrifuged. PBMCs layer was collected and washed twice with PBS. The cells were counted and suspended in the appropriate medium depending on their purpose.

#### *In vitro* cytotoxicity assay with XTT dye

The cytotoxic effect of derivatives of MPA was measured using *in vitro* 2,3-bis(2-methoxy-4-nitro-5-sulfophenyl)-2*H*-tetrazolium-5-carboxanilide (XTT)-based assay (Roche, Basel, Swiss). PBMCs suspended in complete X–VIVO20 medium and T-Jurkat cells suspended in complete RPMI-1640 medium were added on a 96-well plate at a concentration of 5 × 10^5^ cells/50 µL/well. 50 µL of examined compounds, prepared as described in MPA derivatives preparation section) were added to experimental wells to obtain final concentrations: 100; 10; 1; 0.1; 0.01 µM. As a background control we used medium without cells, as a positive control we used cells untreated with compounds. After 48 h for T-Jurkat cells and 72 h for PBMCs of incubation in a humidified atmosphere with 5% CO_2_ at 37 °C, 50 µL of XTT reagent (freshly prepared according to the manufacturer’s procedure) was added to all samples. Plates were incubated for 24 h (5% CO_2_ at 37 °C). The conversion of water-soluble yellow tetrazolium XTT salt into orange formazan was monitored by measuring the optical density (OD) at 450 nm on Agilent BioTek Epoch Microplate Spectrophotometer (Agilent Technologies, Santa Clara, CA), with a background wavelength at 690 nm. Data were expressed as IC_50_ to compounds that inhibited more than 50% of the cell viability. Each sample was performed in triplicates. The final concentration of DMSO in the culture did not influence cellular viability. Cell viability was determined at the highest concentration of the tested compound. For this purpose, TC20 cell counter (Biorad, Hercules, CA) was used with trypan blue exclusion.

#### *In vitro* dye-based proliferation assay

PBMCs and T-Jurkat cells were washed twice with warm PBS (37 °C), suspended in fresh PBS at a final concentration of 10–30 × 10^6^ cells/mL and labelled with 1 µL of VPD450 (BD Biosciences, Franklin Lakes, NJ) at 37 °C for 15 min. Each 5 min cells were vortexed to provide uniform staining^42^^,^^43^. Subsequently, cells were washed with PBS and then with complete medium (X-VIVO20 for PBMCs and RPMI-1640 for T-Jurkat cells). After this step, cells were suspended in fresh complete medium, seeded on 96-well plates (1 × 10^6^ cells/well). PBMCs were additionally stimulated with magnetic beads coated with anti-CD3 and anti-CD28 antibodies (Invitrogen, Carlsbad, CA) in 1:0.5 cell:bead ratio. Of 100 µL of examined derivatives of MPA were added to experimental wells to receive final concentrations: 100; 10; 1; 0.1; 0.01 µM. In parallel, stained and not stimulated cells, as well as stained and stimulated cells, were seeded in the same concentration as controls. After 48 h for T-Jurkat and 72 h for PBMCs, cells were analysed with flow cytometer LSRFortessa (BD Biosciences, Franklin Lakes, NJ) and Kaluza C Flow Cytometry Analysis Software (Beckman Coulter, Brea, CA). Data were expressed as EC_50_ to compounds that inhibited more than 50% of the cell proliferation. Cell viability was determined at the highest concentration of the tested compound. For this purpose, TC20 cell counter (Biorad) was used with trypan blue exclusion.

#### Flow cytometry Annexin V/PI assay

The percentage of apoptotic and necrotic cells after treatments were detected using an FITC Annexin V Apoptosis Detection Kit I (BD Biosciences). According to the manufacturer’s protocol, cells were washed with cold PBS and then resuspended with 1XBinding Buffer at a final concentration of 1 × 10^6^ cells/mL. To 1 × 10^5^ of cells 5 µL of Annexin V-FITC and 5 µL of propidium iodide (PI) were added. After 15 min at room temperature in the dark, flow cytometry analysis was performed. We divided cells into four groups according to Annexin V/PI results: living cells (Annexin V-negative, PI-negative); early apoptotic cells (Annexin V-positive, PI-negative), late apoptotic cells (Annexin V-positive, PI-positive) and necrotic cells (Annexin V-negative, PI-positive). Cells were analysed with flow cytometer LSRFortessa and Kaluza C Flow Cytometry Analysis Software.

#### Statistical analysis

Statistical analysis was performed using GraphPad Prism version 9 (GraphPad Software Inc., La Jolla, CA). Two‐tailed unpaired Student’s *t*‐test was used to evaluate differences between MPA and new derivatives of MPA; differences were considered as statistical significant when *p* < 0.05. EC_50_ and IC_50_ values were calculated by 4‐parametric non‐linear regression using GraphPad Prism version 9. Significance was calculated using the *t*‐test, significant results are marked with *(*p* < 0.05), **(*p* < 0.01), or ***(*p* < 0.001).

## Supplementary Material

Supplemental Material

## References

[1] Hedstrom L. IMP dehydrogenase: structure, mechanism, and inhibition. Chem Rev. 2009;109(7):2903–28. 19480389 10.1021/cr900021w PMC2737513

[2] Sintchak MD, Fleming MA, Futer O, et al. Structure and mechanism of inosine monophosphate dehydrogenase in complex with the immunosuppressant mycophenolic acid. Cell. 1996;85(6):921–30. 8681386 10.1016/s0092-8674(00)81275-1

[3] Yuan S, Gopal JV, Ren S, Chen L, Liu L, Gao Z. Anticancer fungal natural products: mechanisms of action and biosynthesis. Eur J Med Chem. 2020;202:112502. 32652407 10.1016/j.ejmech.2020.112502

[4] Bentley R. Mycophenolic Acid: a one hundred year odyssey from antibiotic to immunosuppressant. Chem Rev. 2000;100(10):3801–26. 11749328 10.1021/cr990097b

[5] Wagner M, Earley AK, Webster AC, et al. Mycophenolic acid versus azathioprine as primary immunosuppression for kidney transplant recipients. Cochrane Database Syst Rev. 2015;3(12):CD007746. 26633102 10.1002/14651858.CD007746.pub2 PMC10986644

[6] Sahman M, Mugosa S, Rancic N. Utilization of mycophenolic acid, azathioprine, tacrolimus, cyclosporin, sirolimus, and everolimus: multinational study. Front Public Health. 2021;9:671316. 33869136 10.3389/fpubh.2021.671316 PMC8044364

[7] Song X, Tu R, Mei X, Wu S, Lan B, Zhang L, Luo X, Liu J, Luo M. A mycophenolic acid derivative from the fungus penicillium sp. SCSIO sof101. Nat Prod Res. 2020;34(9):1206–12. 30760051 10.1080/14786419.2018.1553881

[8] Pilevneli AD, Ebada SS, Kaşkatepe B, Konuklugil B. Penicacids H–J, three new mycophenolic acid derivatives from the marine-derived fungus *Rhizopus oryzae*. RSC Adv. 2021;11(55):34938–44. 35494752 10.1039/d1ra07196c PMC9043025

[9] Klangjorhor J, Chaiyawat P, Teeyakasem P, Sirikaew N, Phanphaisarn A, Settakorn J, Lirdprapamongkol K, Yama S, Svasti J, Pruksakorn D. Mycophenolic acid is a drug with the potential to be repurposed for suppressing tumor growth and metastasis in osteosarcoma treatment. Int J Cancer. 2020;146(12):3397–409. 31609477 10.1002/ijc.32735

[10] Hirunsatitpron P, Hanprasertpong N, Noppakun K, Pruksakorn D, Teekachunhatean S, Koonrungsesomboon N. Mycophenolic acid and cancer risk in solid organ transplant recipients: Systematic review and meta-analysis. Br J Clin Pharmacol. 2022;88(2):476–89. 34240462 10.1111/bcp.14979

[11] Benjanuwattra J, Chaiyawat P, Pruksakorn D, Koonrungsesomboon N. Therapeutic potential and molecular mechanism of mycophenolic acid as an anticancer agents. Eur J Pharmacol. 2020;887:173580. 32949604 10.1016/j.ejphar.2020.173580

[12] Cholewinski G, Malachowska-Ugarte M, Dzierzbicka K. The chemistry of mycophenolic acid - synthesis and modifications towards desired biological activity. Curr Med Chem. 2010;17(18):1926–41. 20377512 10.2174/092986710791163920

[13] Lee S, Ku AF, Vippila MR, Wang Y, Zhang M, Wang X, Hedstrom L, Cuny GD. Mycophenolic anilides as broad specificity inosine-5’-monophosphate dehydrogenase (IMPDH) inhibitors. Bioorg Med Chem Lett. 2020;30(24):127543. 32931912 10.1016/j.bmcl.2020.127543 PMC9511823

[14] Batovska DI, Kim DH, Mitsuhashi S, Cho YS, Kwon HJ, Ubukata M. Hydroxamic acid derivatives of mycophenolic acid inhibit histone deacetylase at the cellular level. Biosci Biotechnol Biochem. 2008;72(10):2623–31. 18838793 10.1271/bbb.80303

[15] Sunohara K, Mitsuhashi S, Shigetomi K, Ubukata M. Discovery of N-(2,3,5-triazoyl)mycophenolic amide and mycophenolic epoxyketone as novel inhibitors of human IMPDH. Bioorg Med Chem Lett. 2013;23(18):5140–44. 23937979 10.1016/j.bmcl.2013.07.016

[16] Peng Y, Dong Y, Mahato RI. Synthesis and characterization of a novel mycophenolic acid–quinic acid conjugate serving as immunosuppressant with decreased toxicity. Mol Pharm. 2015;12(12):4445–53. 26529468 10.1021/acs.molpharmaceut.5b00639

[17] Felczak K, Vince R, Pankiewicz KW. NAD-based inhibitors with anticancer potential. Bioorg Med Chem Lett. 2014;24(1):332–6. 24269162 10.1016/j.bmcl.2013.11.005

[18] Siebert A, Wysocka M, Krawczyk B, Cholewiński G, Rachoń J. Synthesis and antimicrobial activity of amino acid and peptide derivatives of mycophenolic acid. Eur J Med Chem. 2018;143:646–55. 29216563 10.1016/j.ejmech.2017.11.094 PMC7173178

[19] Shah CP, Kharkar PS. Newer human inosine 5′-monophosphate dehydrogenase 2 (hIMPDH2) inhibitors as potential anticancer agents. J Enzyme Inhib Med Chem. 2018;33(1):972–7. 29792360 10.1080/14756366.2018.1474211 PMC6009919

[20] Shang FF, Wang MY, Ai JP, Shen QK, Guo HY, Jin CM, Chen FE, Quan ZS, Jin L, Zhang C. Synthesis and evaluation of mycophenolic acid derivatives as potential antiToxoplasma gondii agents. Med Chem Res. 2021;30(12):2228–39.

[21] Juvale K, Shaik A, Kirubakaran S. Inhibitors of inosine 5′-monophosphate dehydrogenase as emerging new generation antimicrobial agents. Medchemcomm. 2019;10(8):1290–301. 31534651 10.1039/c9md00179d PMC6727467

[22] Shah CP, Kharkar PS. Discovery of novel human inosine 5,-monophosphate dehydrogenase 2 (*h*IMPDH2) inhibitors as potential anticancer agents. Eur J Med Chem. 2018;158:286–301. 30223117 10.1016/j.ejmech.2018.09.016

[23] Cholewiński G, Iwaszkiewicz-Grześ D, Prejs M, Głowacka A, Dzierzbicka K. Synthesis of the inosine 5'-monophosphate dehydrogenase (IMPDH) inhibitors. J Enzyme Inhib Med Chem. 2015;30(4):550–63. 25198892 10.3109/14756366.2014.951349

[24] Martins P, Jesus J, Santos S, Raposo LR, Roma-Rodrigues C, Baptista PV, Fernandes AR. Heterocyclic anticancer compounds: recent advances and the paradigm shift towards the use of nanomedicine’s tool box. Molecules. 2015;20(9):16852–91. 26389876 10.3390/molecules200916852 PMC6331900

[25] Vitaku E, Smith DT, Njardarson JT. Analysis of the structural diversity, substitution patterns, and frequency of nitrogen heterocycles among U.S. FDA approved pharmaceuticals. J Med Chem. 2014;57(24):10257–74. 25255204 10.1021/jm501100b

[26] Gorla SK, Kavitha M, Zhang M, Chin JEW, Liu X, Striepen B, Makowska-Grzyska M, Kim Y, Joachimiak A, Hedstrom L, et al. Optimization of benzoxazole-based inhibitors of cryptosporidium parvum Inosine 5′-monophosphate dehydrogenase. J Med Chem. 2013;56(10):4028–43. 23668331 10.1021/jm400241j PMC3756936

[27] Paramashivappa R, Phani Kumar P, Subba Rao PV, Srinivasa Rao A. Design, synthesis and biological evaluation of benzimidazole/benzothiazole and benzoxazole derivatives as cyclooxygenase inhibitors. Bioorg Med Chem Lett. 2003;13(4):657–60. 12639552 10.1016/s0960-894x(02)01006-5

[28] Ghorab M, Bashandy M, Alsaid M. Novel thiophene derivatives with sulfonamide, isoxazole, benzothiazole, quinoline and anthracene moieties as potential anticancer agents. Acta Pharm. 2014;64(4):419–31. 25531783 10.2478/acph-2014-0035

[29] Husain A, Rashid M, Shaharyar M, Siddiqui AA, Mishra R. Benzimidazole clubbed with triazolo-thiadiazoles and triazolo-thiadiazines: new anticancer agents. Eur J Med Chem. 2013;62:785–98. 23333063 10.1016/j.ejmech.2012.07.011

[30] Golden EB, Cho H-Y, Hofman FM, Louie SG, Schönthal AH, Chen TC. Quinoline-based antimalarial drugs: a novel class of autophagy inhibitors. Neurosurg Focus. 2015;38(3):E12. 10.3171/2014.12.FOCUS14748 25727221

[31] Volochnyuk D, Grygorenko O, Gorlova A. Fluorine-containing diazines in medicinal chemistry and agrochemistry. J Fluor Chem. 2014;2:577–672.

[32] Irfan A, Batool F, Zahra Naqvi SA, Islam A, Osman SM, Nocentini A, Alissa SA, Supuran CT. Benzothiazole derivatives as anticancer agents. J Enzyme Inhib Med Chem. 2020;35(1):265–79. 31790602 10.1080/14756366.2019.1698036 PMC6896476

[33] Abrol S, Bodla RB, Goswani C. A comprehensive review on benzothiazole derivatives for their biological activities. Int J Pharm Sci Res. 2019;10:3196–209.

[34] El-Faham A, Albericio F. Peptide coupling reagents, more than a letter soup. Chem Rev. 2011;111(11):6557–602. 21866984 10.1021/cr100048w

[35] Carpino LA, Ionescu D, El-Faham A. Peptide coupling in the presence of highly hindered tertiary amines. J Org Chem. 1996;61(7):2460–65.

[36] Beyermann M, Henklein P, Klose A, Sohr R, Bienert M. Effect of tertiary amine on the carbodiimide-mediated peptide synthesis. Int J Pept Protein Res. 1991;37(4):252–6. 1894440 10.1111/j.1399-3011.1991.tb00737.x

[37] Jain A, Yang G, Yalkowsky SH. Estimation of melting points of organic compounds. Ind Eng Chem Res. 2004;43(23):7618–21.

[38] Larina LI. Tautomerism and structure of azoles: nuclear magnetic resonance spectroscopy. Adv Heterocycl Chem. 2018;124:233–321.

[39] Sawhney SN, Boykin DW. Transmission of substituent effects in heterocyclic systems by carbon-13 nuclear magnetic resonance Benzothiazoles. J Org Chem. 1979;44(7):1136–42.

[40] Cholewinski G, Iwaszkiewicz-Grzes D, Trzonkowski P, Dzierzbicka K. Synthesis and biological activity of ester derivatives of mycophenolic acid and acridines/acridones as potential immunosuppressive agents. J Enzyme Inhib Med Chem. 2016;31(6):974–82. 26308114 10.3109/14756366.2015.1077821

[41] Prejs M, Cholewiński G, Trzonkowski P, Kot-Wasik A, Dzierzbicka K. Synthesis and antiproliferative activity of new mycophenolic acid conjugates with adenosine derivatives. J Asian Nat Prod Res. 2019;21(2):178–85. 29607657 10.1080/10286020.2018.1451521

[42] Siebert A, Cholewiński G, Trzonkowski P, Rachon J. Immunosuppressive properties of amino acid and peptide derivatives of mycophenolic acid. Eur J Med Chem. 2020;189:112091. 32007665 10.1016/j.ejmech.2020.112091

[43] Ten Brinke A, Marek-Trzonkowska N, Mansilla MJ, et al. Monitoring T-cell responses in translational studies: optimization of dye-based proliferation assay for evaluation of antigen-specific responses. Front Immunol. 2017;8:1870. 29312346 10.3389/fimmu.2017.01870 PMC5742609

[44] Mitsuhashi S, Takenaka J, Iwamori K, Nakajima N, Ubukata M. Structure-activity relationships for inhibition of inosine monophosphate dehydrogenase and differentiation induction of K562 cells among the mycophenolic acid derivatives. Bioorg Med Chem. 2010;18(22):8106–11. 20934342 10.1016/j.bmc.2010.09.004

[45] Suzuki T, Hisakawa S, Itoh Y, Suzuki N, Takahashi K, Kawahata M, Yamaguchi K, Nakagawa H, Miyata N. Design, synthesis, and biological activity of folate receptor-targeted prodrugs of thiolate histone deacetylase inhibitors. Bioorg Med Chem Lett. 2007;17(15):4208–12. 17532630 10.1016/j.bmcl.2007.05.040

[46] Park H, Kim S, Kim YE, Lim SJ. A structure-based virtual screening approach toward the discovery of histone deacetylase inhibitors: identification of promising zinc-chelating groups. ChemMedChem. 2010;5(4):591–7. 20157916 10.1002/cmdc.200900500

[47] Tung TT, Oanh DTK, Dung PTP, Hue VTM, Park SH, Han BW, Kim Y, Hong J-T, Han S-B, Nam NH, et al. New benzothiazole/thiazole-containing hydroxamic acids as potent histone deacetylase inhibitors and antitumor agents. Med Chem. 2013;9(8):1051–7. 23521008 10.2174/15734064113099990027

[48] HyperChem(TM) Professional 7.51, 1115 NW 4th Street. Gainesville (FL): Hypercube, Inc. https://hyperchem.software.informer.com/. [accessed 1 May 2022].

[49] Hocquet A, Langgård M. An evaluation of the MM + force field. J Mol Model. 1998;4(3):94–112.

[50] Trott O, Olson AJ. AutoDock Vina: improving the speed and accuracy of docking with a new scoring function, efficient optimization, and multithreading. J Comput Chem. 2010;31(2):455–61. 19499576 10.1002/jcc.21334 PMC3041641

[51] Humphrey W, Dalke A, Schulten K. VMD: visual molecular dynamics. J Mol Graph. 1996;14(1):33–8, 27–8. 8744570 10.1016/0263-7855(96)00018-5

[52] Iwaszkiewicz-Grzes D, Cholewinski G, Kot-Wasik A, Trzonkowski P, Dzierzbicka K. Investigations on the immunosuppressive activity of derivatives of mycophenolic acid in immature dendritic cells. Int Immunopharmacol. 2017;44:137–42. 28092865 10.1016/j.intimp.2017.01.011

