# Supplementary material for: Novel amides of mycophenolic acid and some heterocyclic derivatives as immunosuppressive agents
Source: J Enzyme Inhib Med Chem. 2022 Oct 3;37(1):2725–41. doi: 10.1080/14756366.2022.2127701 (PMC9542285; doi:10.1080/14756366.2022.2127701)
Supplement: Supplemental Material [file IENZ_A_2127701_SM2619.pdf]

Electronic Supplementary Information (ESI)

Novel amides of mycophenolic acid and some heterocyclic derivatives as immunosuppressive agents

Juliusz Maksymilian Walczak[a], Dorota Iwaszkiewicz-Grześ[b,*], Michalina Ziomkowska[c], Magdalena Śliwka-Kaszyńska[a], Mateusz Daśko[d], Piotr Trzonkowski[b], Grzegorz Cholewiński[a,*]

[a] *Department of Organic Chemistry, Faculty of Chemistry, Gdańsk University of Technology, G. Narutowicza 11/12, 80-233 Gdańsk, Poland*
[b] *Department of Medical Immunology, Faculty of Medicine, Medical University of Gdańsk, Dębinki 7, 80-211 Gdańsk, Poland*
[c] *Perlan Technologies sp. z o.o., Puławska 303, 02-785 Warszawa, Poland*
[d] *Department of Inorganic Chemistry, Faculty of Chemistry, Gdańsk University of Technology, G. Narutowicza 11/12, 80-233 Gdańsk, Poland*

Supplementary materials .... **Page**

^{1}H and ^{13}C NMR spectra of **MPA** 2–3

^{1}H and ^{13}C NMR spectra of **A1-A18** 4–39

HPLC chromatograms for **MPA** 40

HPLC chromatograms for **A1-A18** 41-58

MS spectra of **MPA** 59

MS spectra of **A1-A18** 60-77

Brief study on melting points 78-80

PBMCs proliferation measurements 81

A7 and **A18** as IMPDH inhibitors — mechanistic approach 82

* — Corresponding authors; e-mail: dorota.iwaszkiewicz-grzes@gumed.edu.pl; Dębinki 7, 80-211 Gdańsk, Poland; e-mail: grzegorz.cholewinski@pg.edu.pl; G. Narutowicza 11/12, 80-233 Gdańsk, Poland;

Mycophenolic acid (**MPA**):

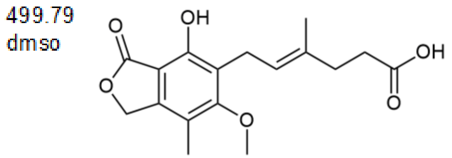

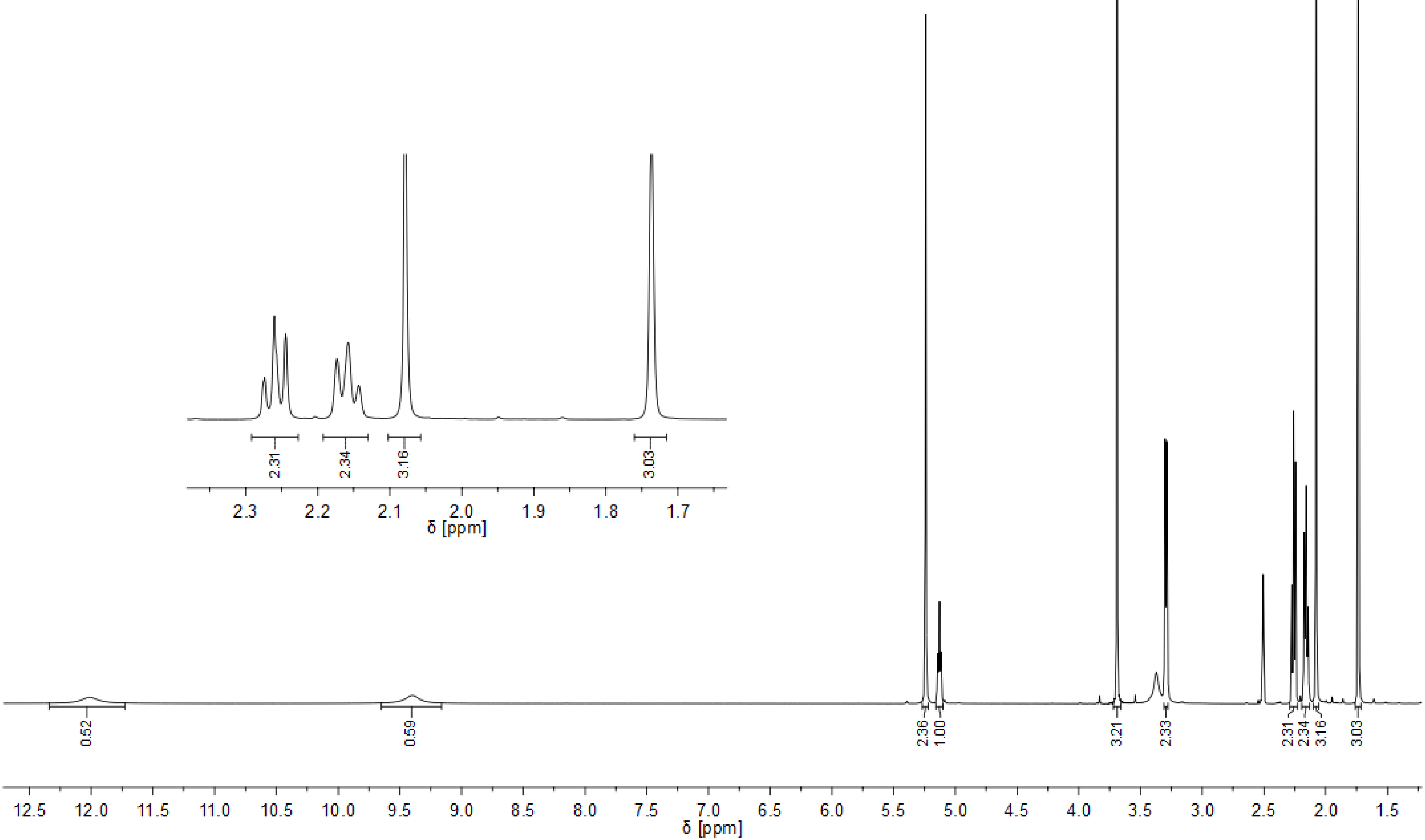

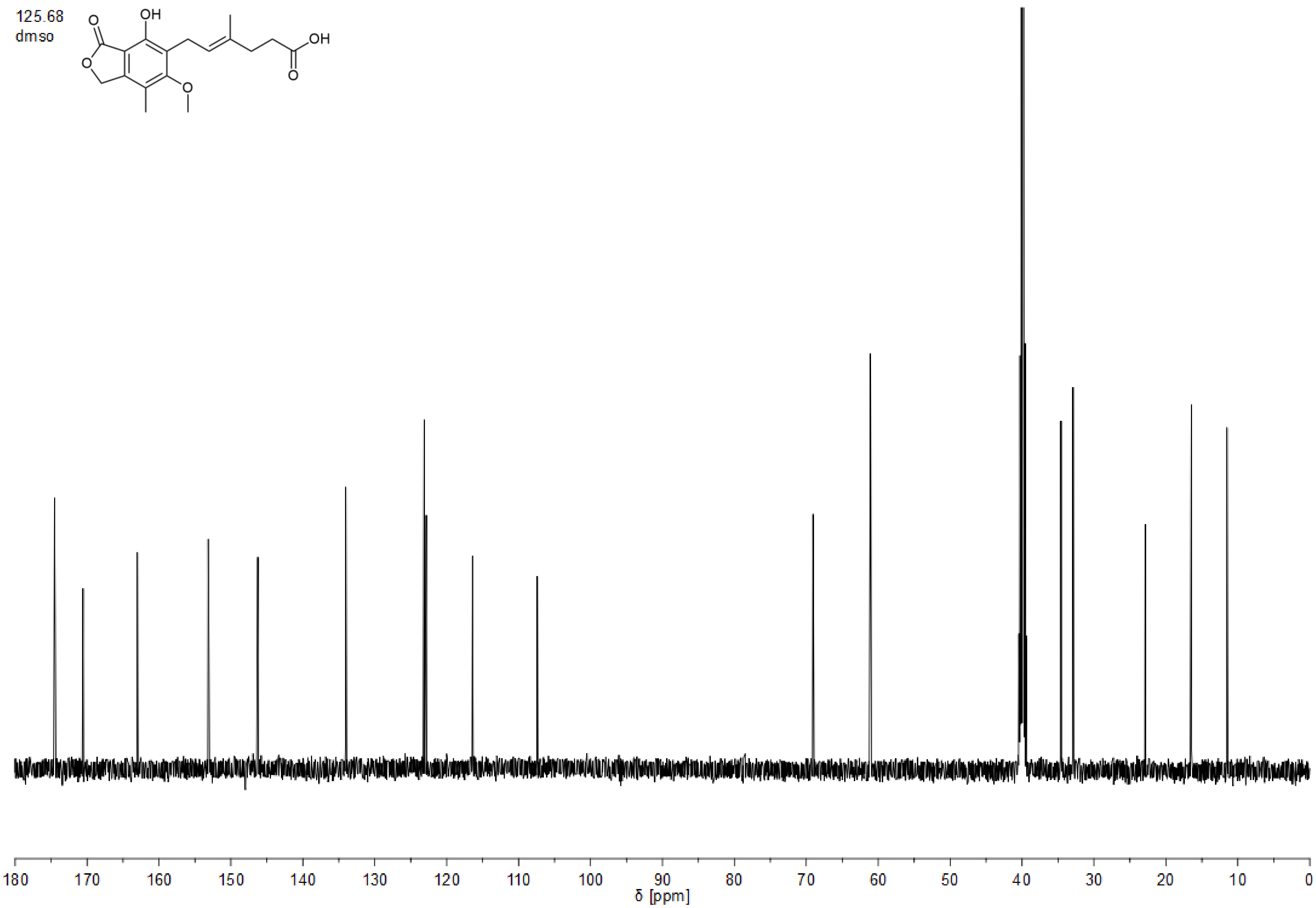
125.68
dmso
OH
O
O
OH
O
O
180
170
160
150
140
130
120
110
100
90
80
70
60
50
40
30
20
10
0
δ [ppm]

N-(benzo[*d*]thiazol-2-yl) mycophenolate (**A1**):

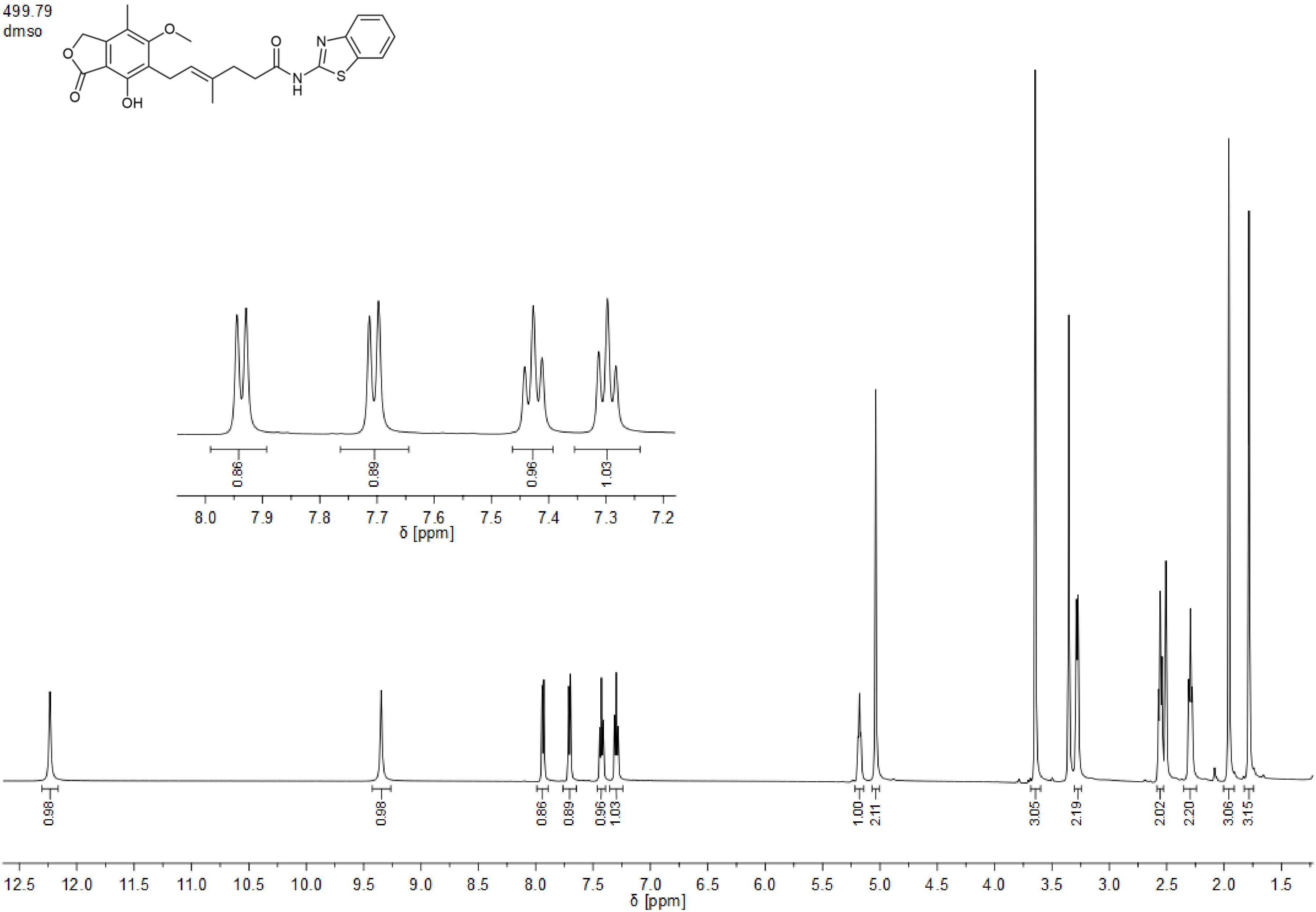

125.68
dmso

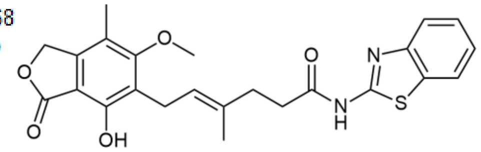

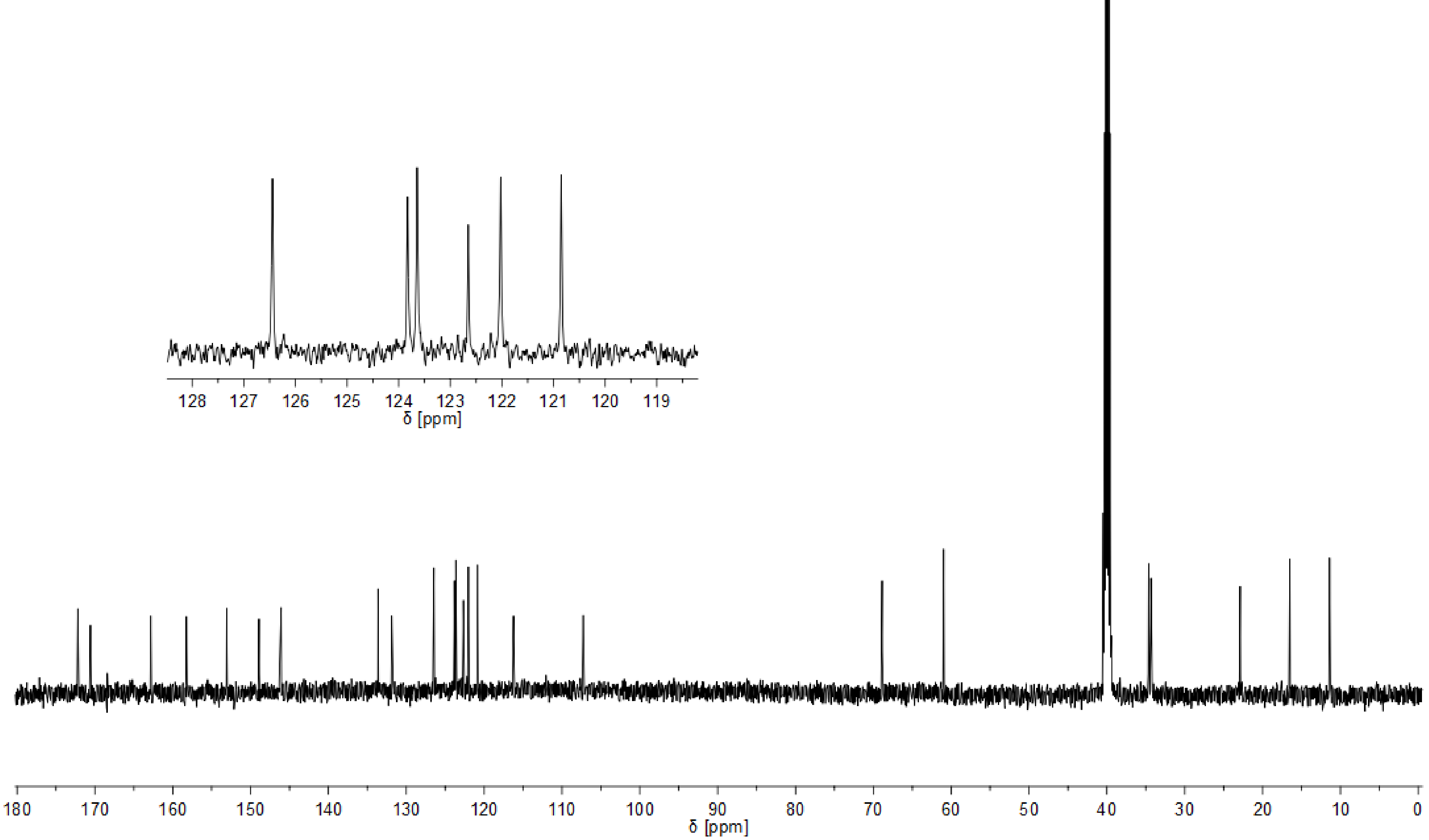

128 127 126 125 124 123 122 121 120 119
δ [ppm]
180 170 160 150 140 130 120 110 100 90 80 70 60 50 40 30 20 10 0
δ [ppm]

N-(benzo[*d*]oxazol-2-yl) mycophenolate (**A2**):

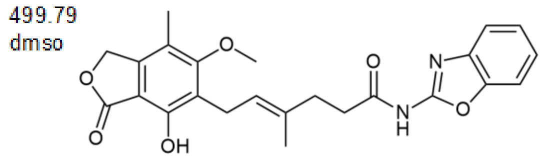

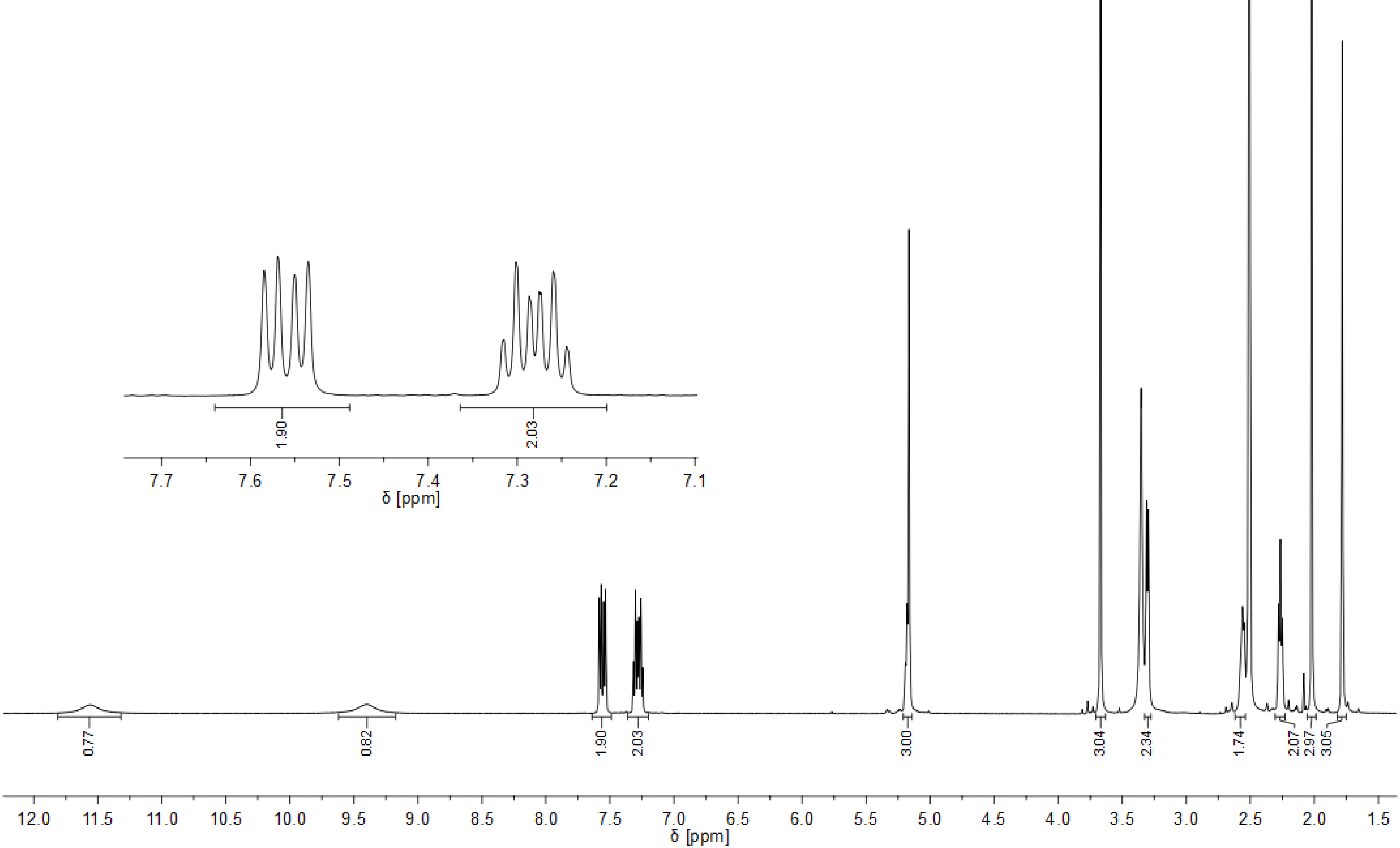

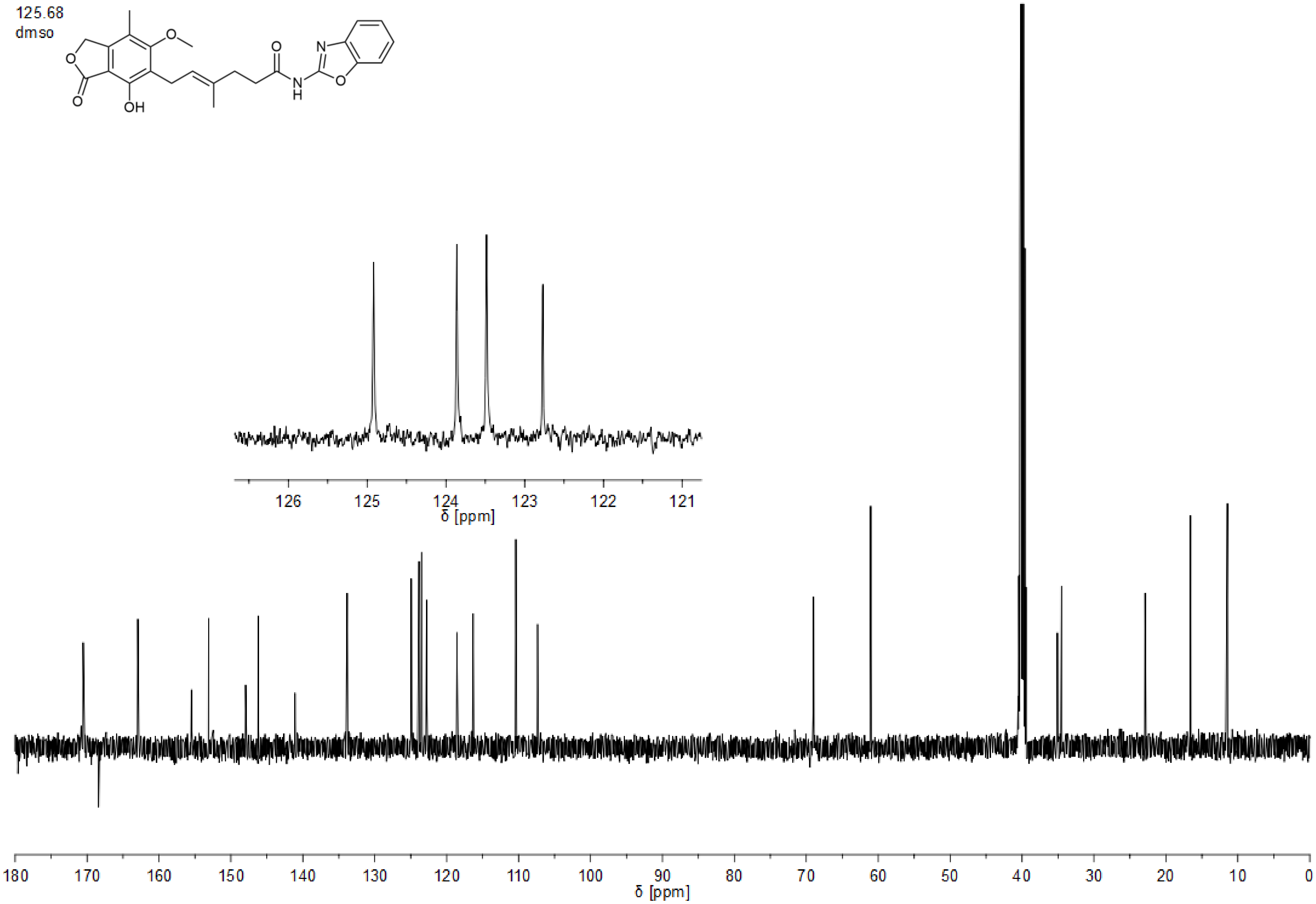
125.68
dmso
O
O
O
OH
O
N
H
N
O
δ [ppm]
δ [ppm]

N-(1*H*-benzo[*d*]imidazol-2-yl) mycophenolate (**A3**):

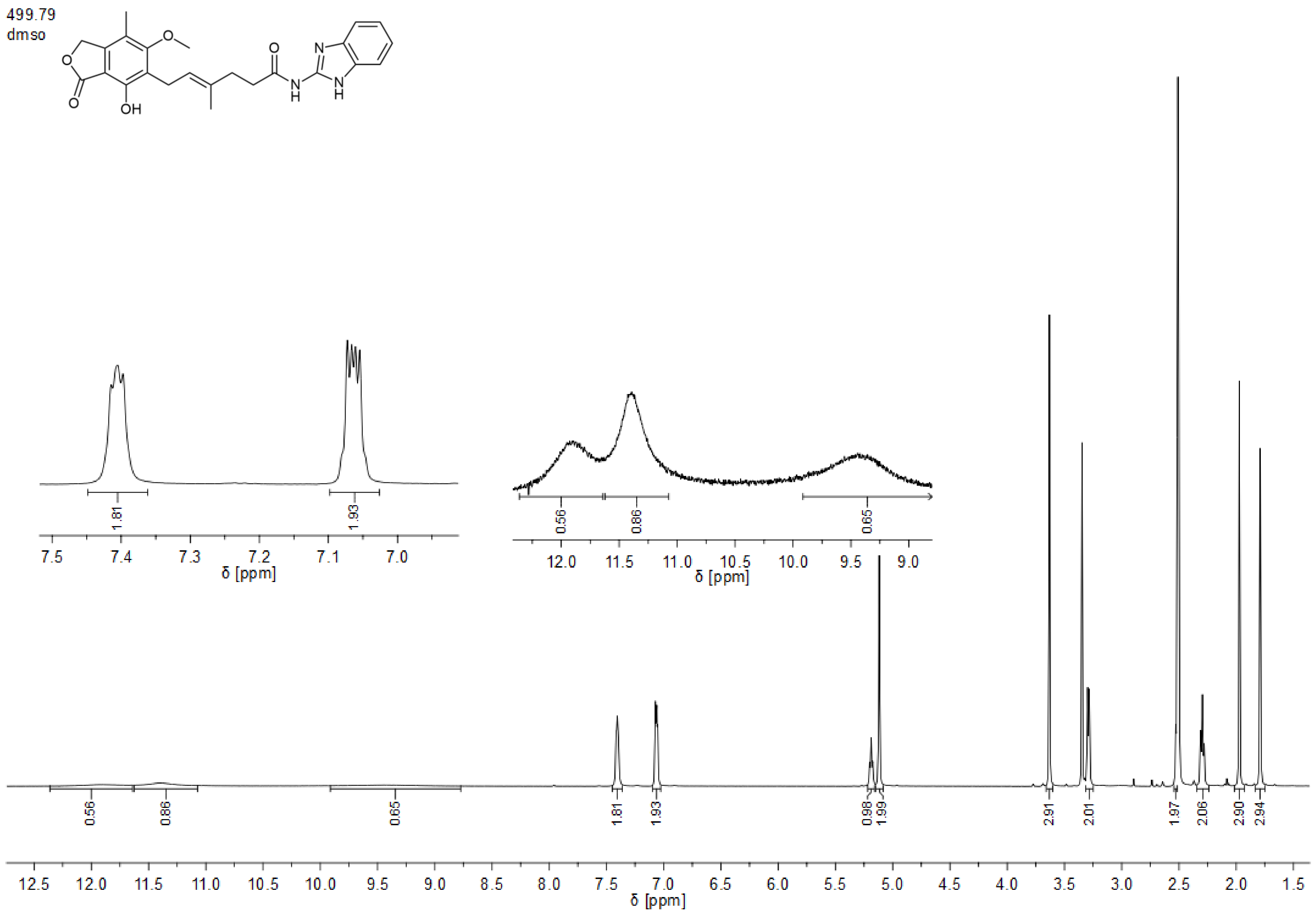

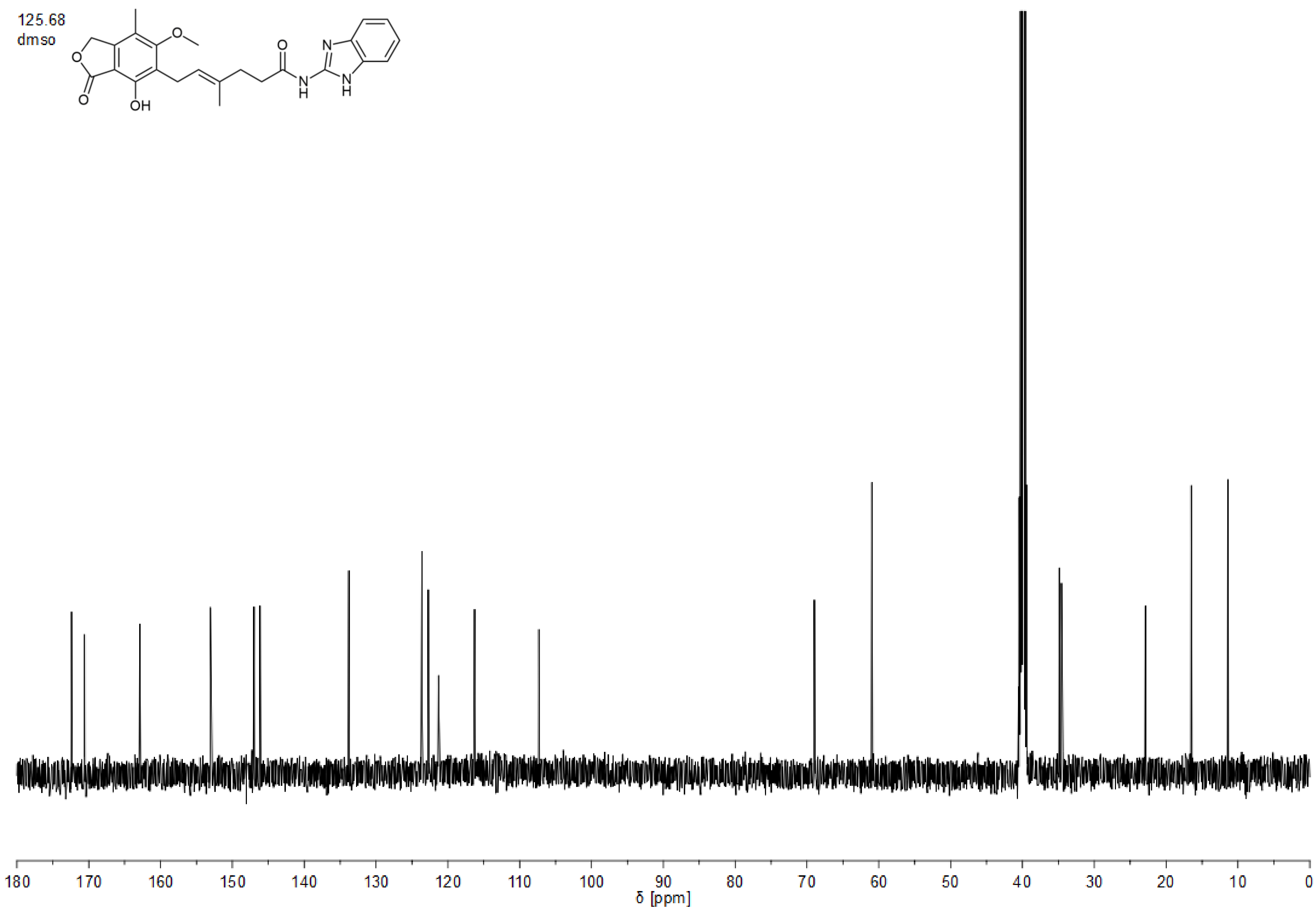

125.68
dmso
O
O
OH
O
N
N
H
N
H
O
180
170
160
150
140
130
120
110
100
90
80
70
60
50
40
30
20
10
0
δ [ppm]

N-(benzo[*d*]thiazol-6-yl) mycophenolate (**A4**):

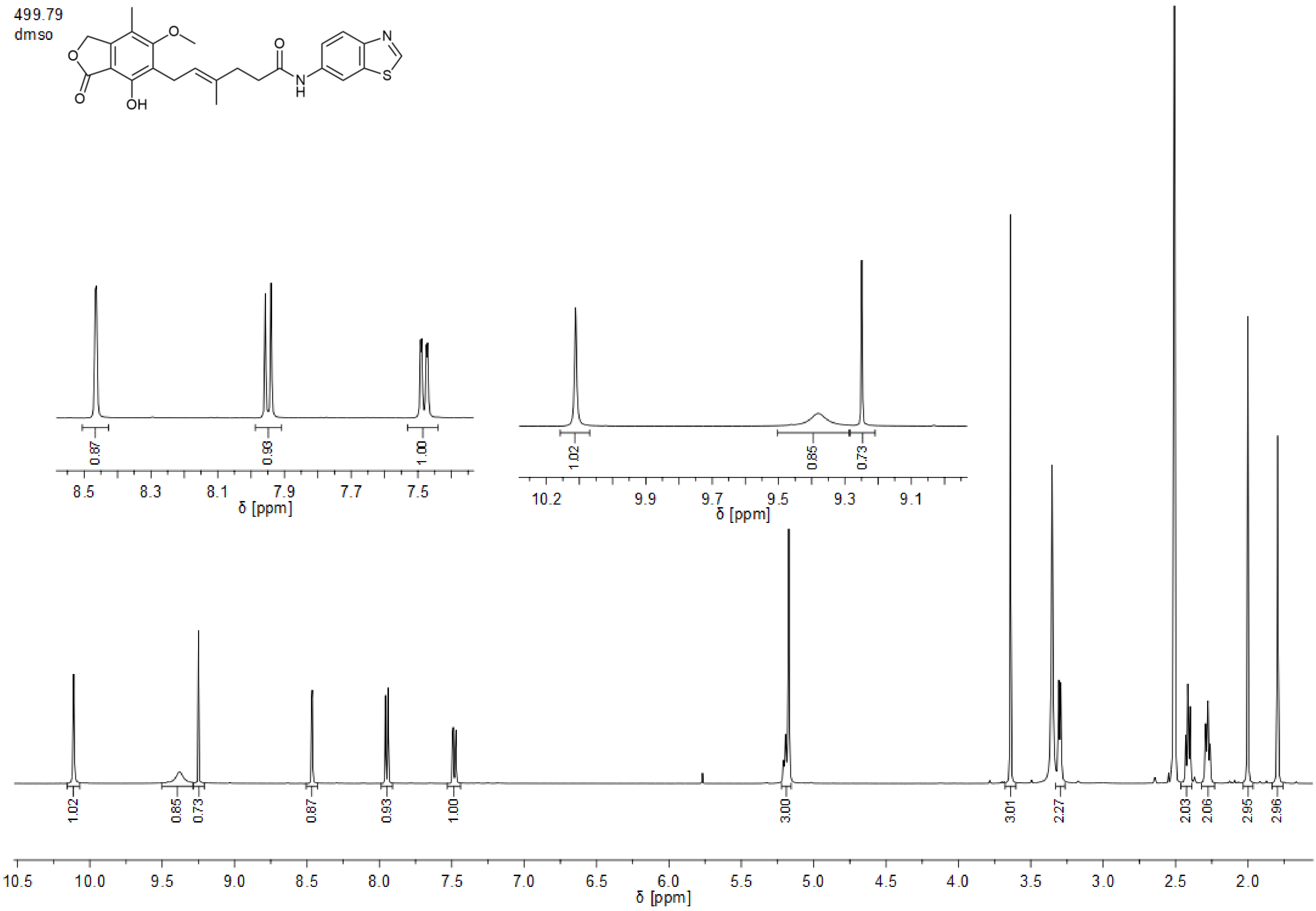

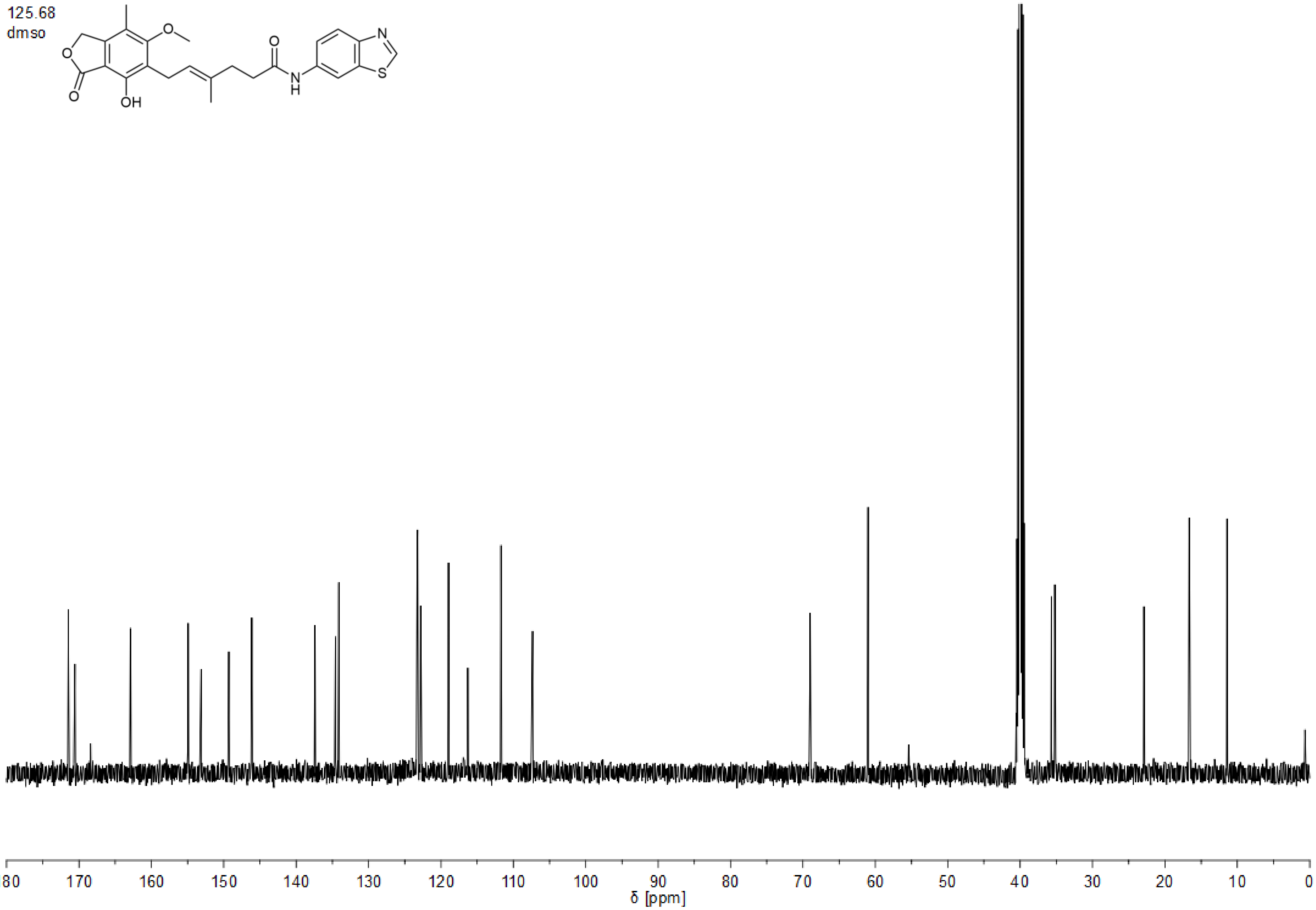
125.68
dmso
O
O
O
OH
O
N
H
N
S
180 170 160 150 140 130 120 110 100 90 80 70 60 50 40 30 20 10 0
δ [ppm]

N-(benzo[*d*]thiazol-5-yl) mycophenolate (**A5**):

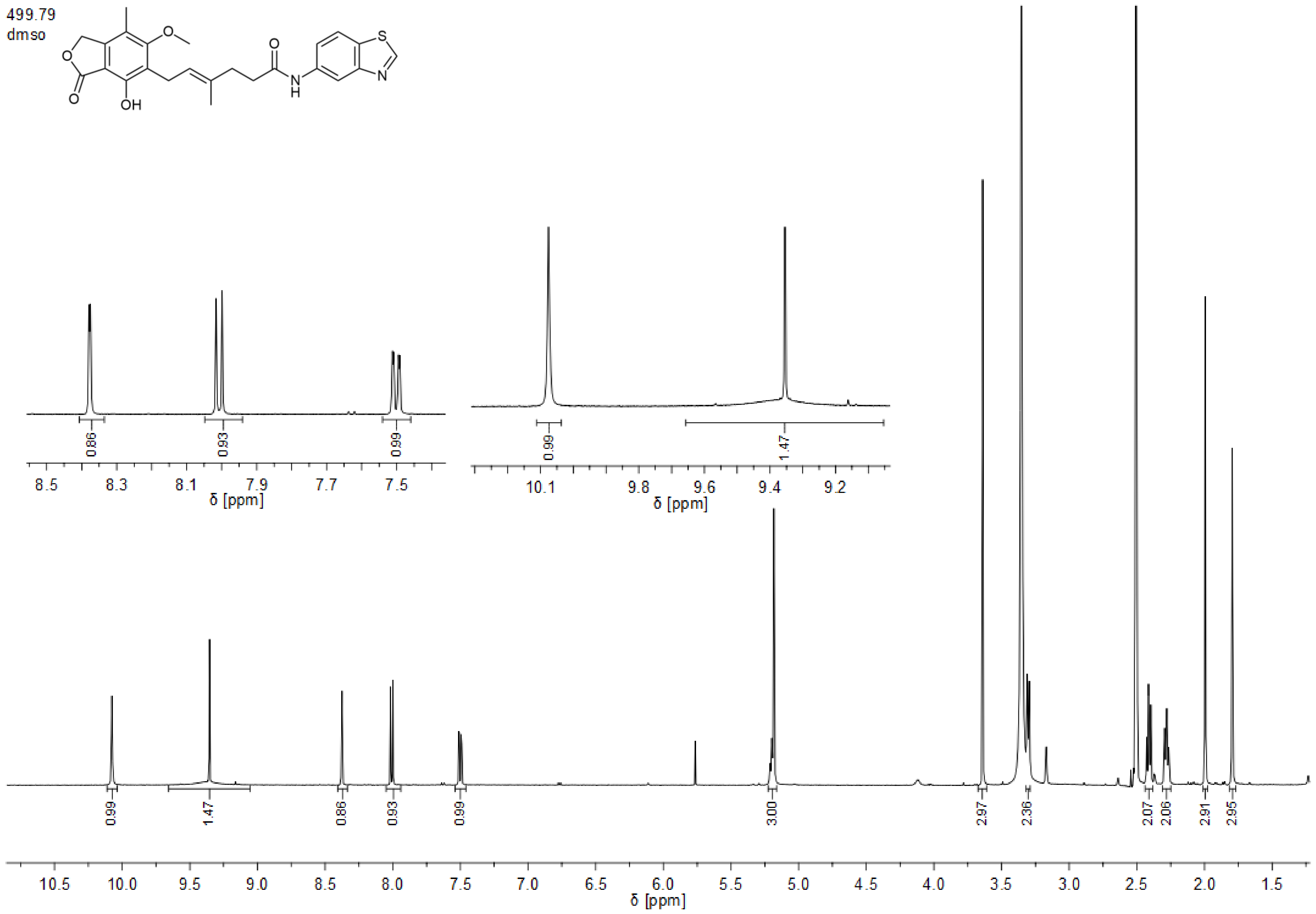

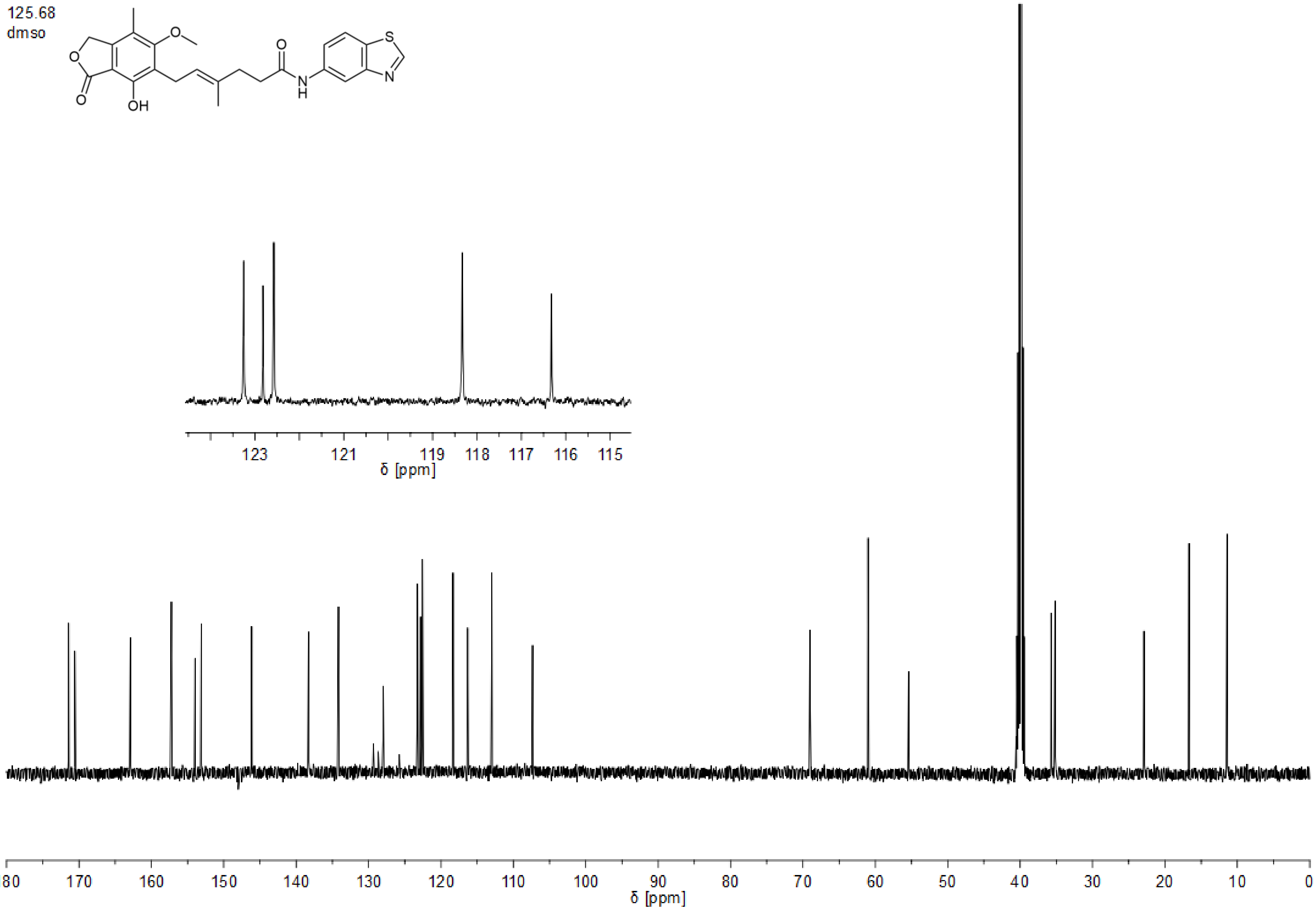
125.68
dmso
O
OH
N
H
S
N
123
121
119
118
117
116
115
δ [ppm]
180
170
160
150
140
130
120
110
100
90
80
70
60
50
40
30
20
10
0
δ [ppm]

N-(benzo[*d*]oxazol-5-yl) mycophenolate (**A6**):

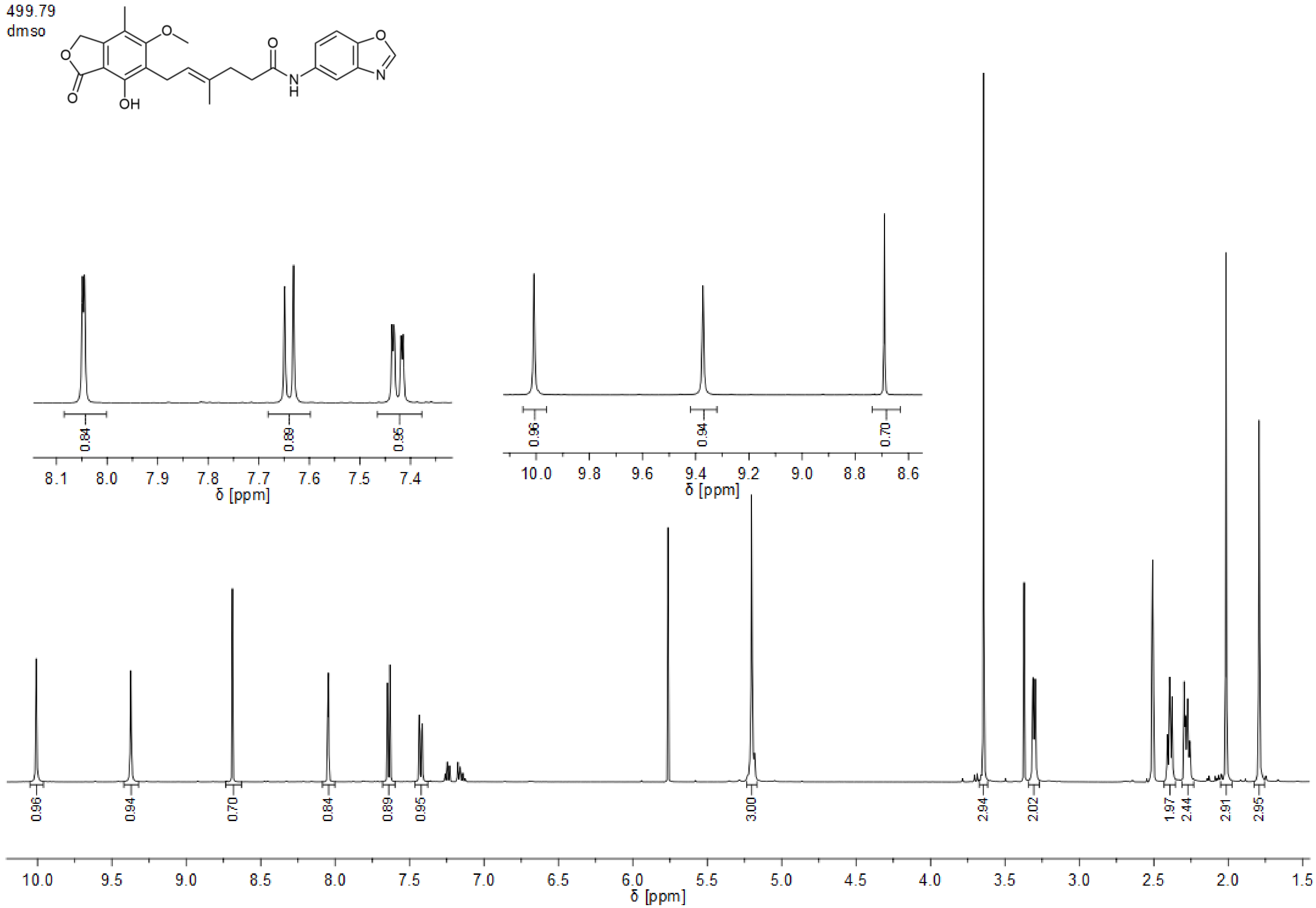

125.68
dmso

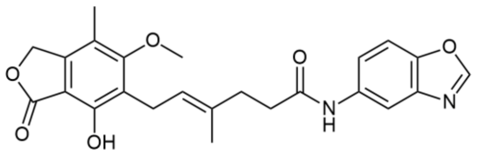
O
O
O
OH
O
N
H
O
N

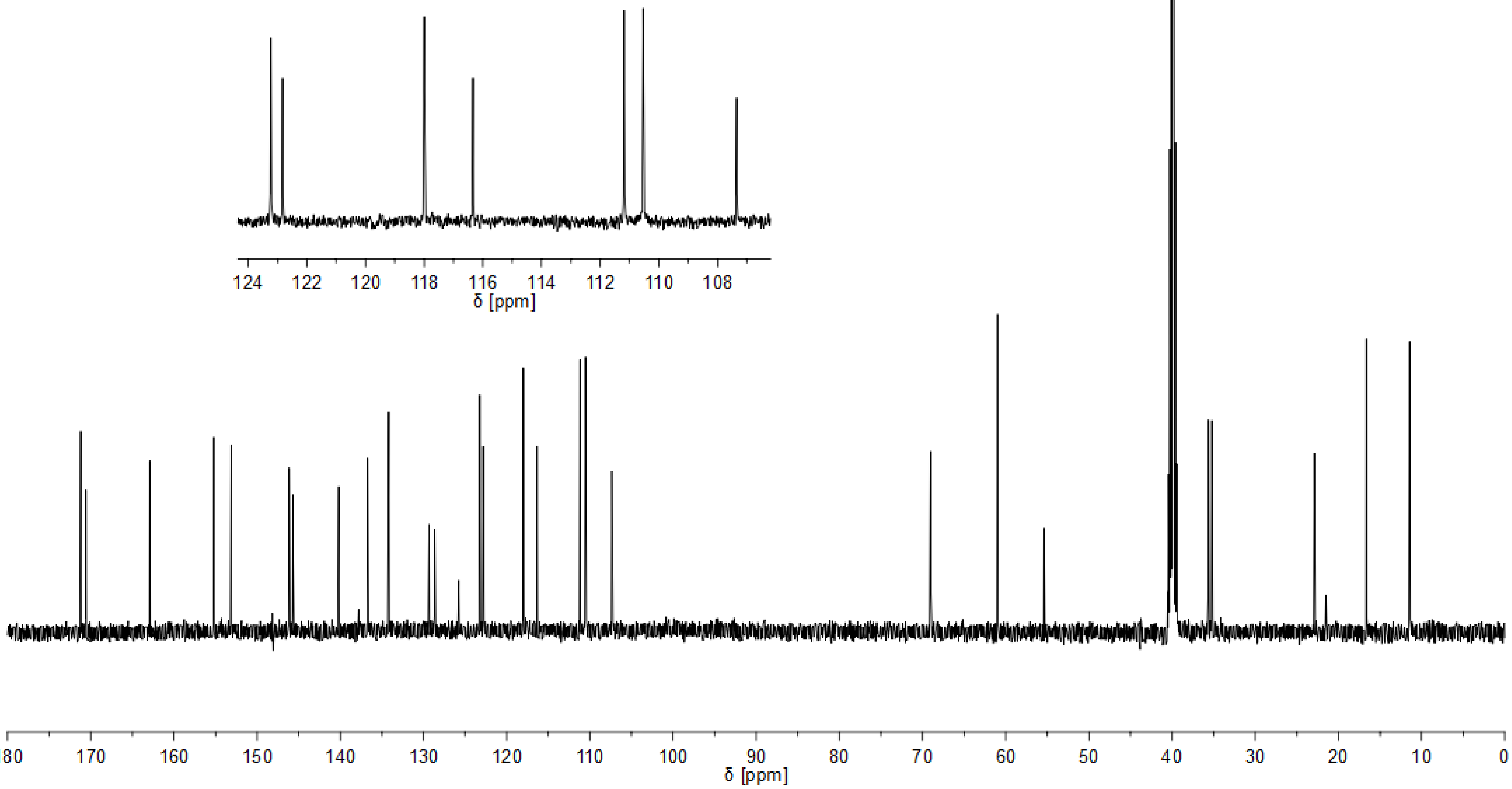
124 122 120 118 116 114 112 110 108
δ [ppm]
180 170 160 150 140 130 120 110 100 90 80 70 60 50 40 30 20 10 0
δ [ppm]

N-[1-(pirymidin-2-yl)methyl] mycophenolate (**A7**):

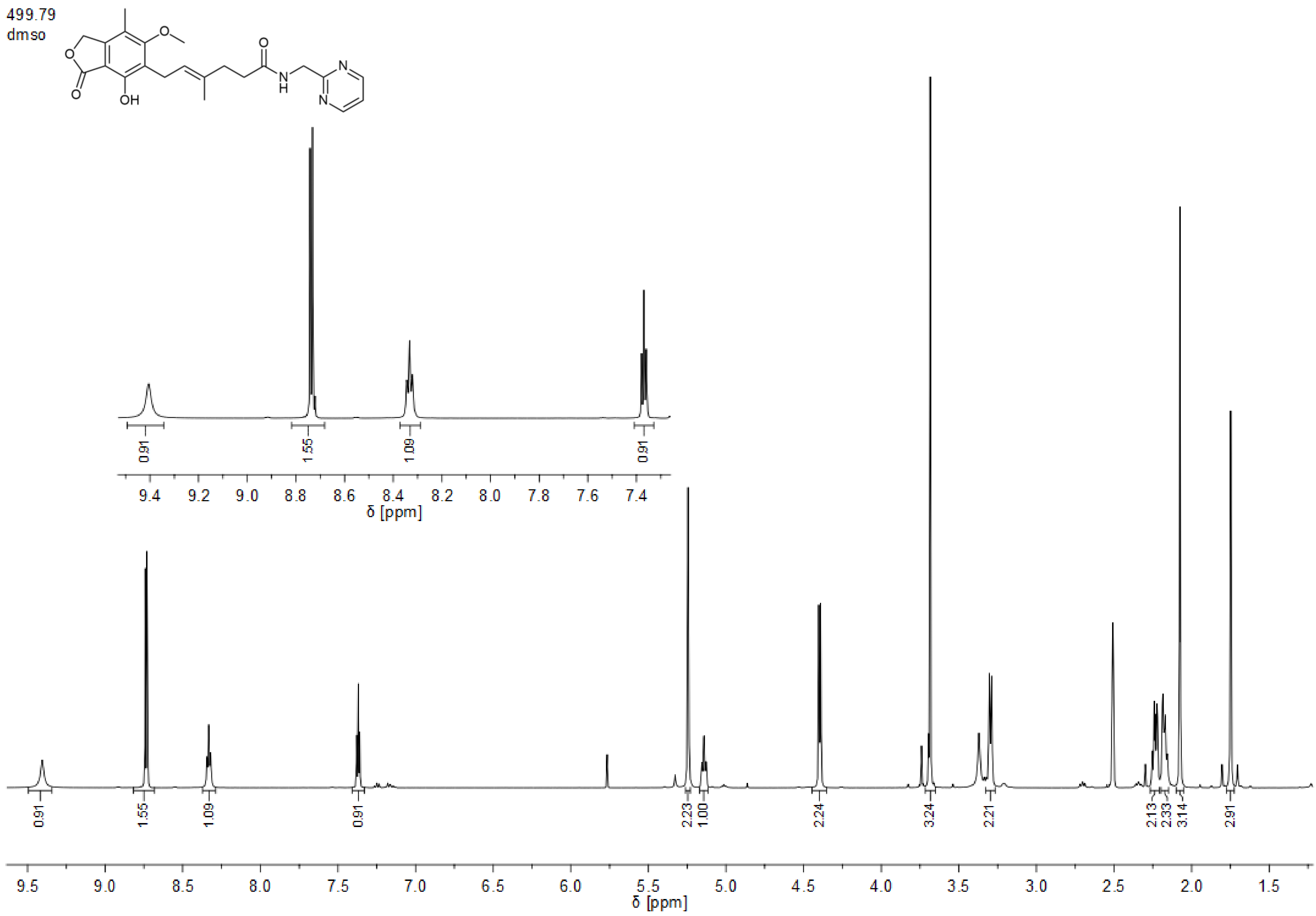

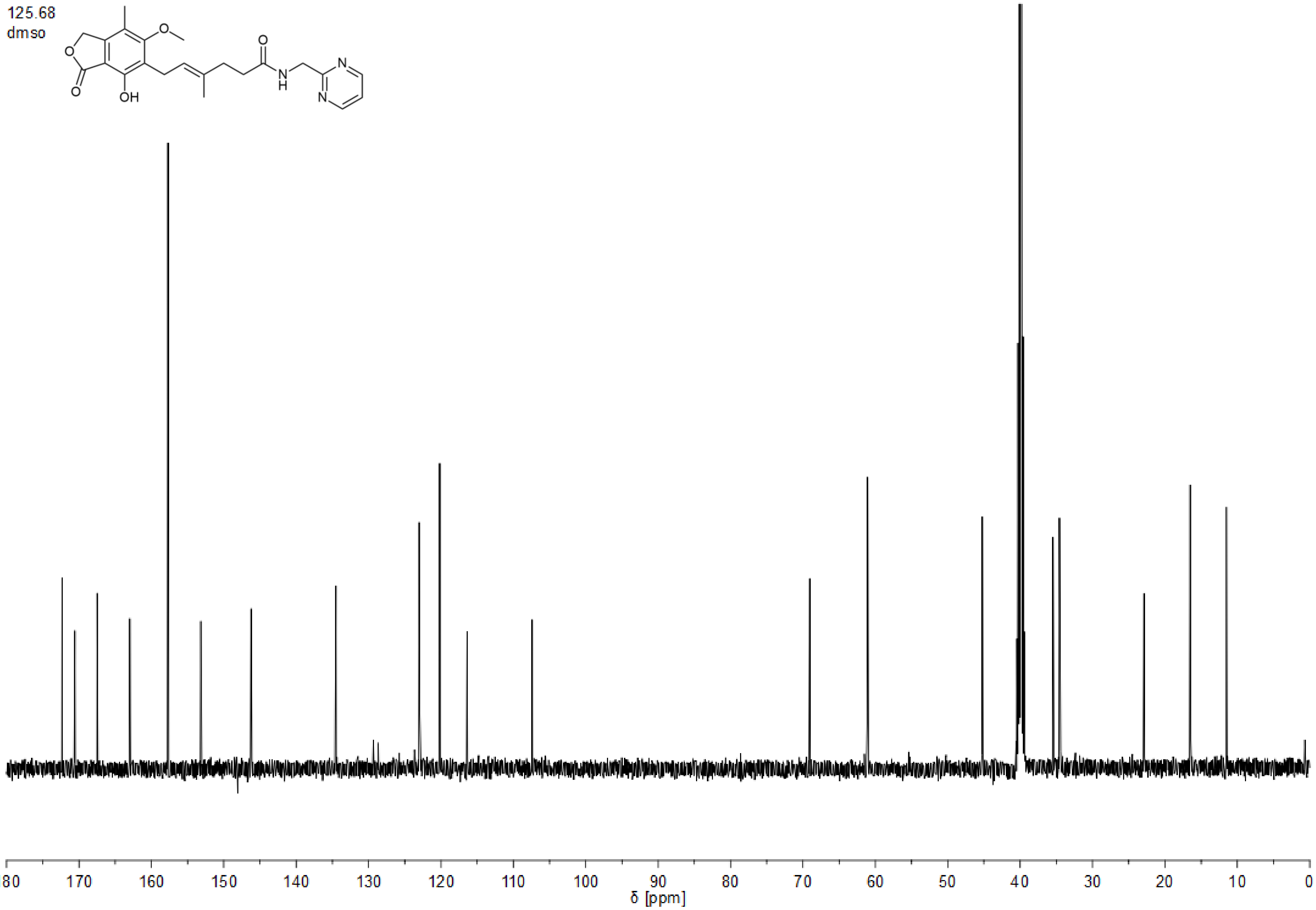
125.68
dmso
O
OH
O
O
O
N
H
N
N
δ [ppm]
180
170
160
150
140
130
120
110
100
90
80
70
60
50
40
30
20
10
0

N-(6-methoxybenzo[*d*]thiazol-2-yl) mycophenolate (**A8**):

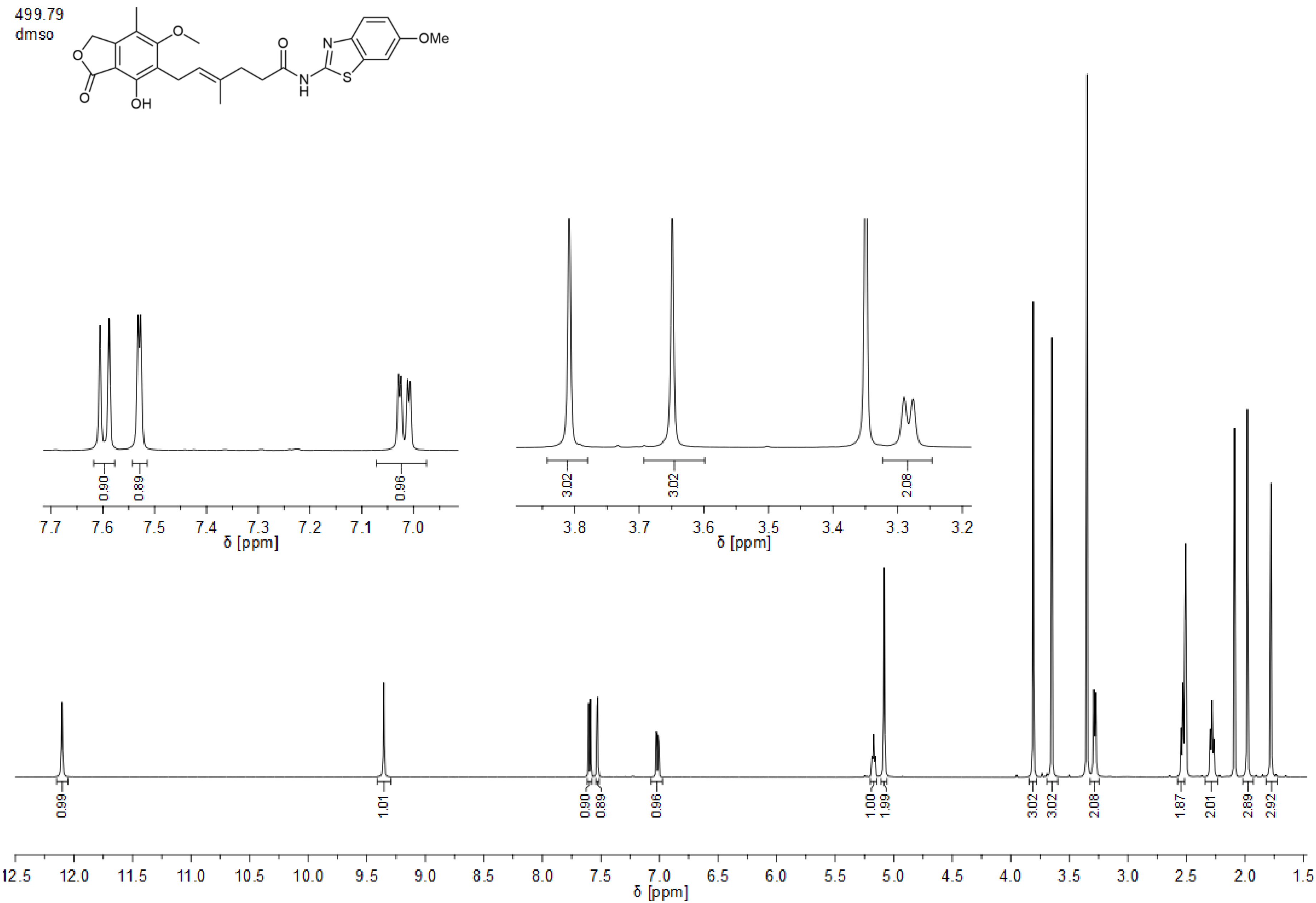

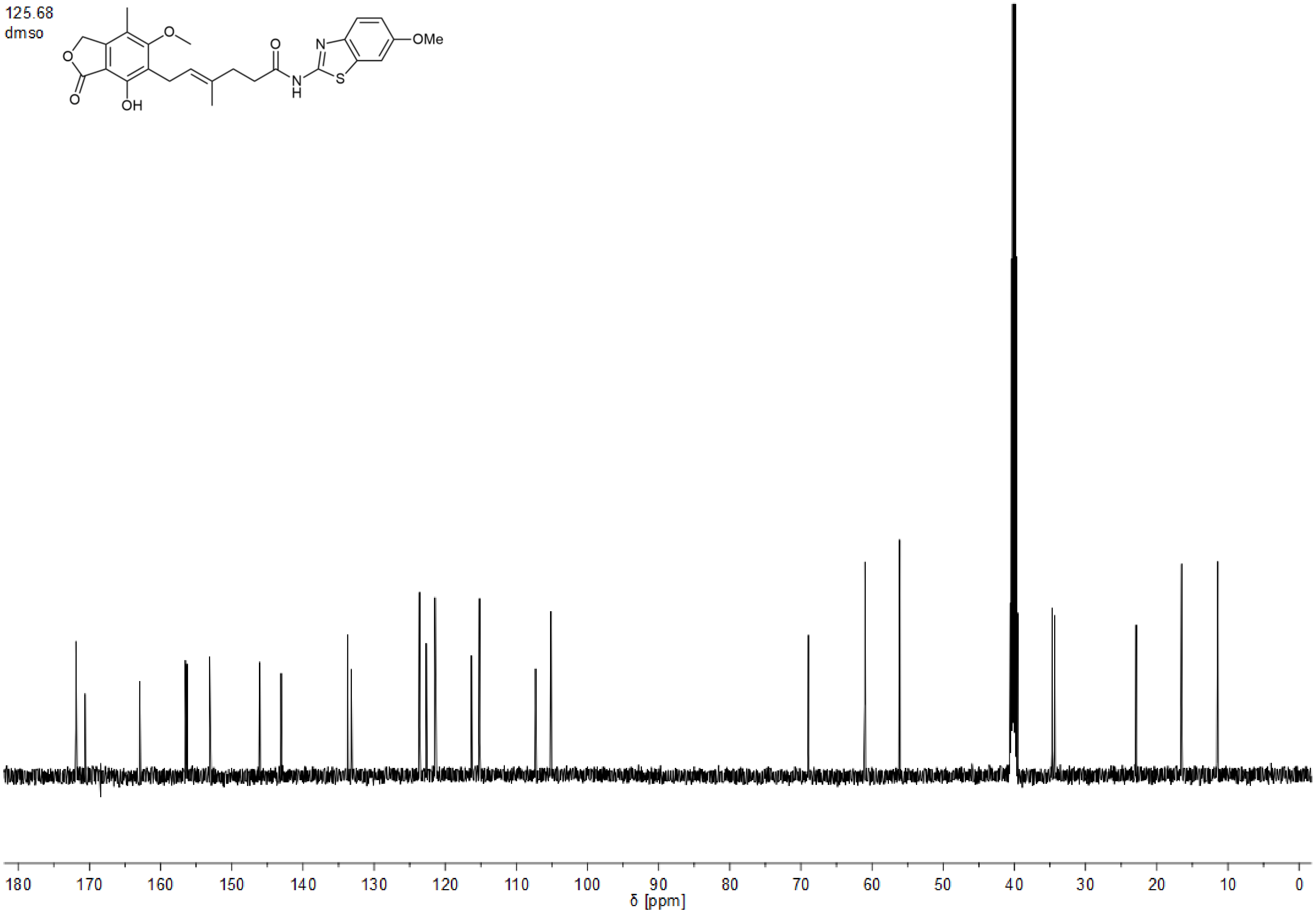

125.68
dmso
OH
O
N
H
S
OMe
180 170 160 150 140 130 120 110 100 90 80 70 60 50 40 30 20 10 0
δ [ppm]

N-(6-methylbenzo[*d*]thiazol-2-yl) mycophenolate (**A9**):

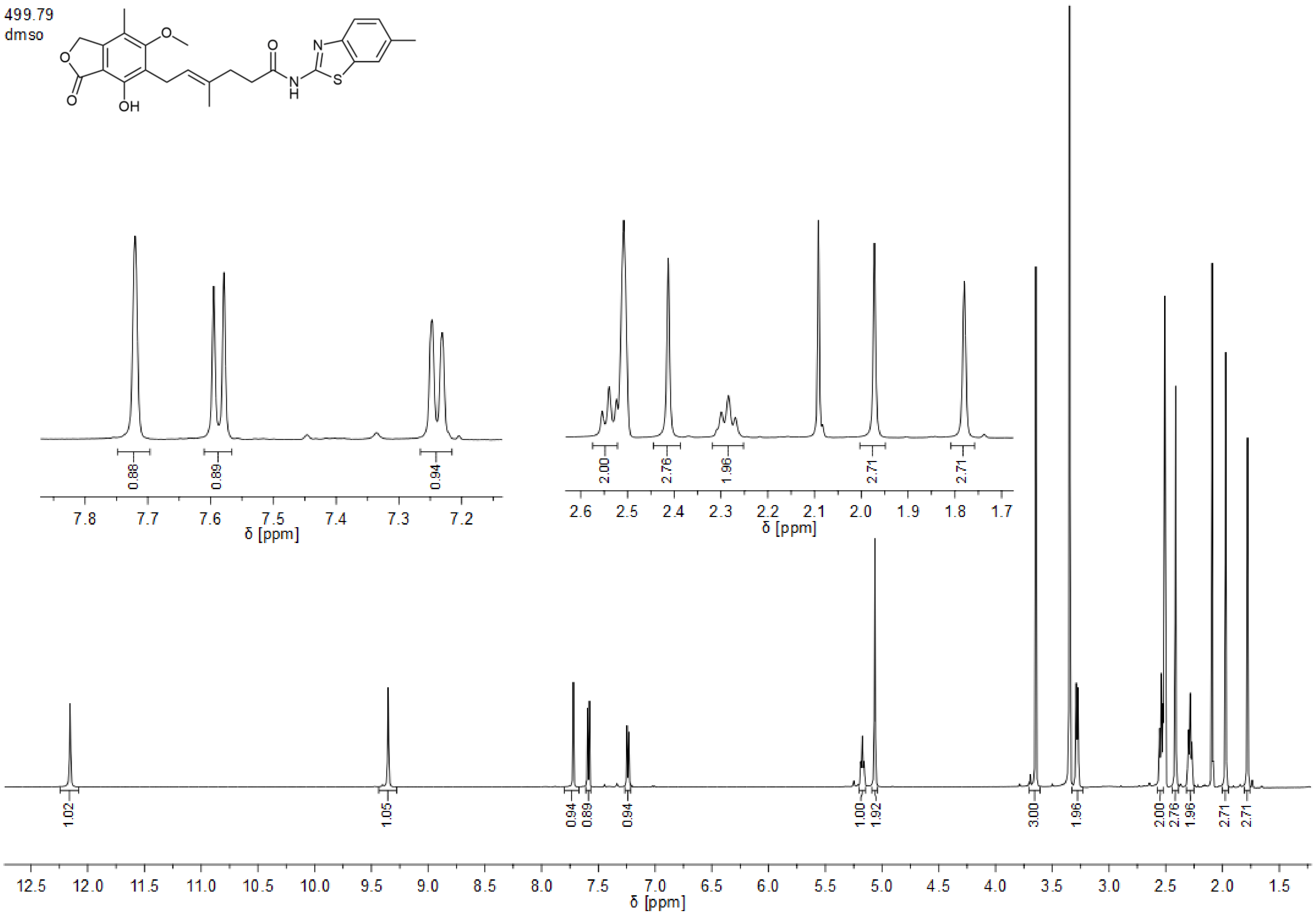

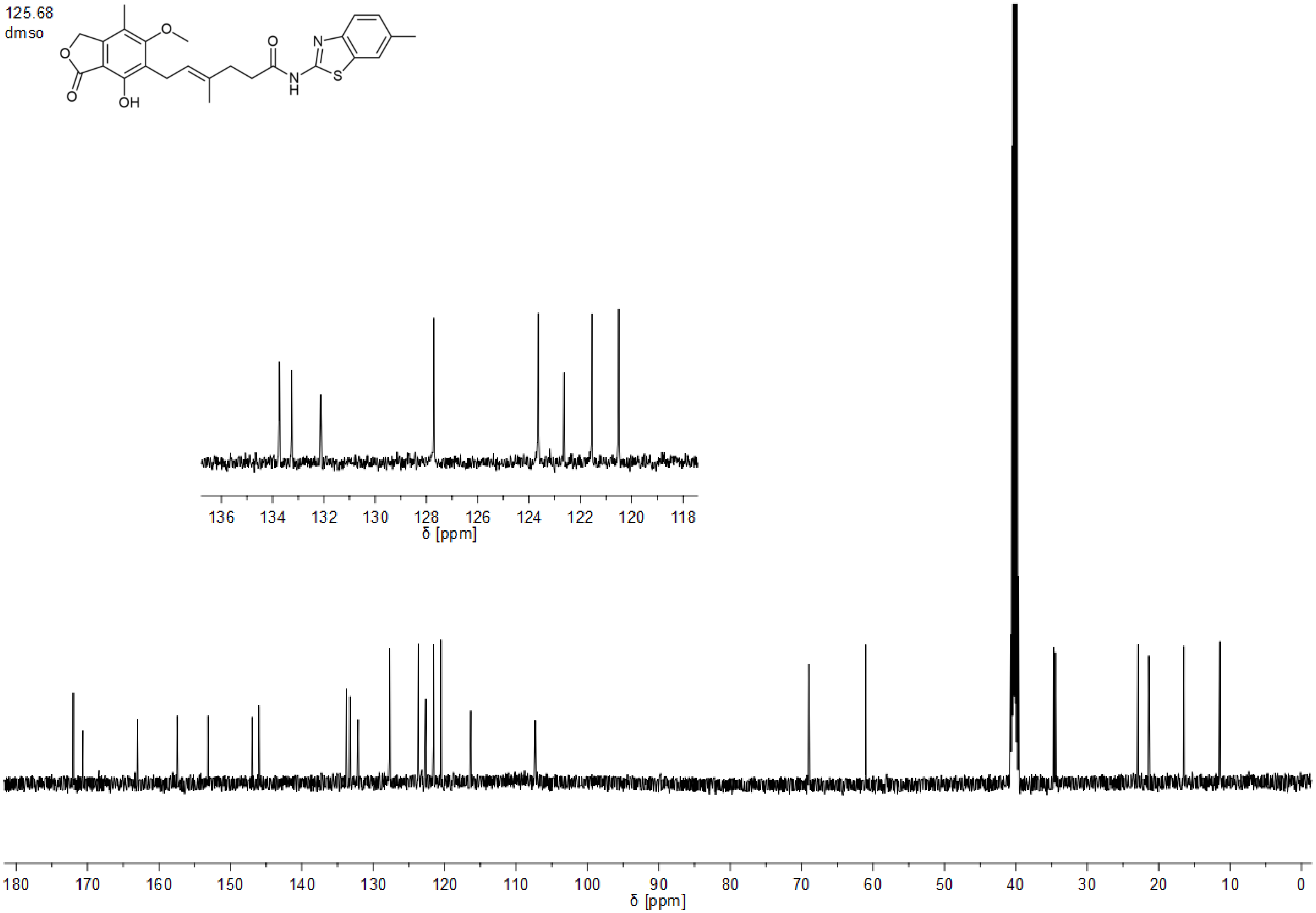
125.68
dmso
OH
δ [ppm]
δ [ppm]

N-(5,6-dimethylbenzo[*d*]thiazol-2-yl) mycophenolate (**A10**):

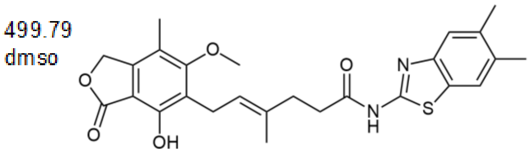

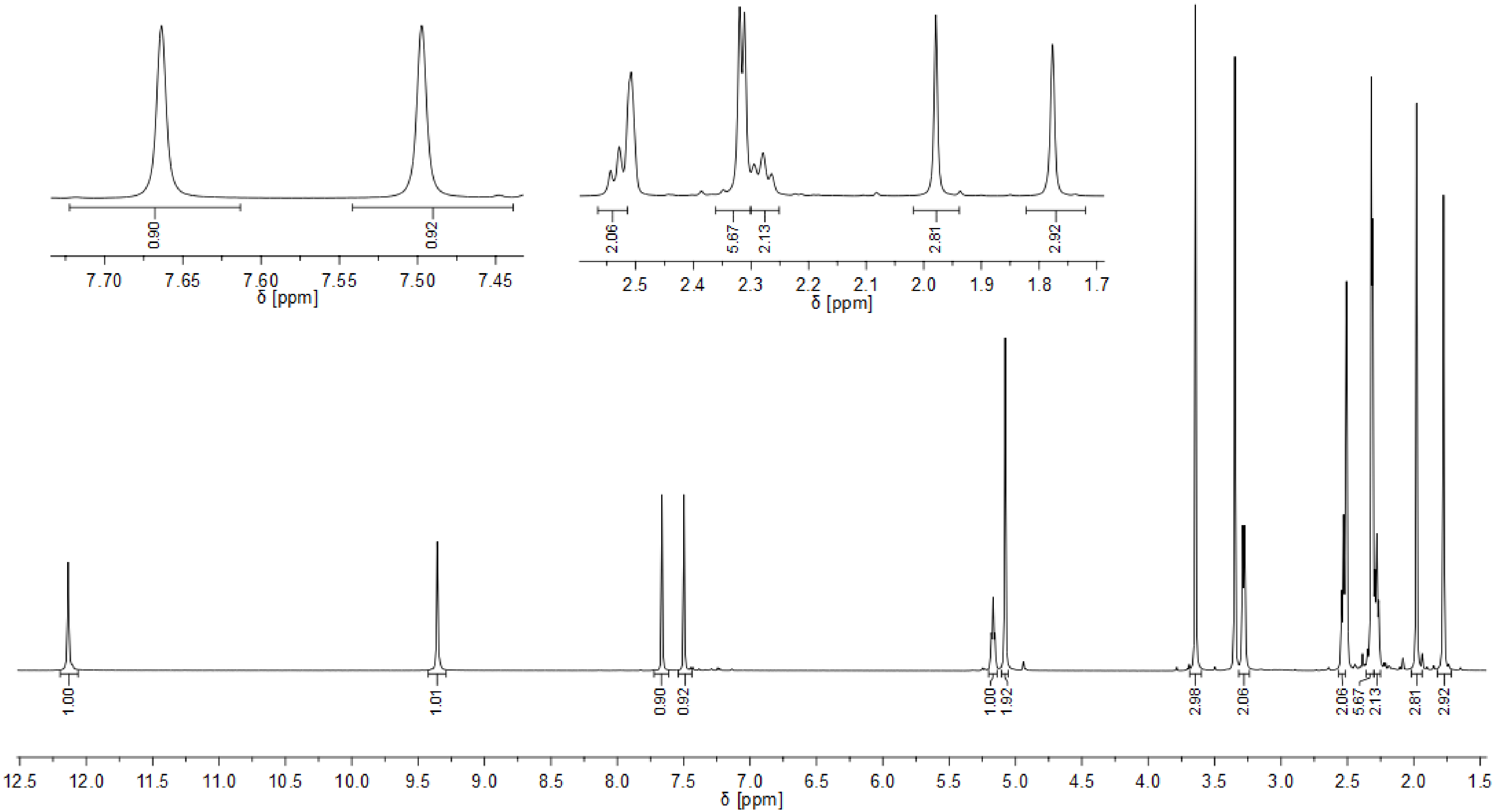

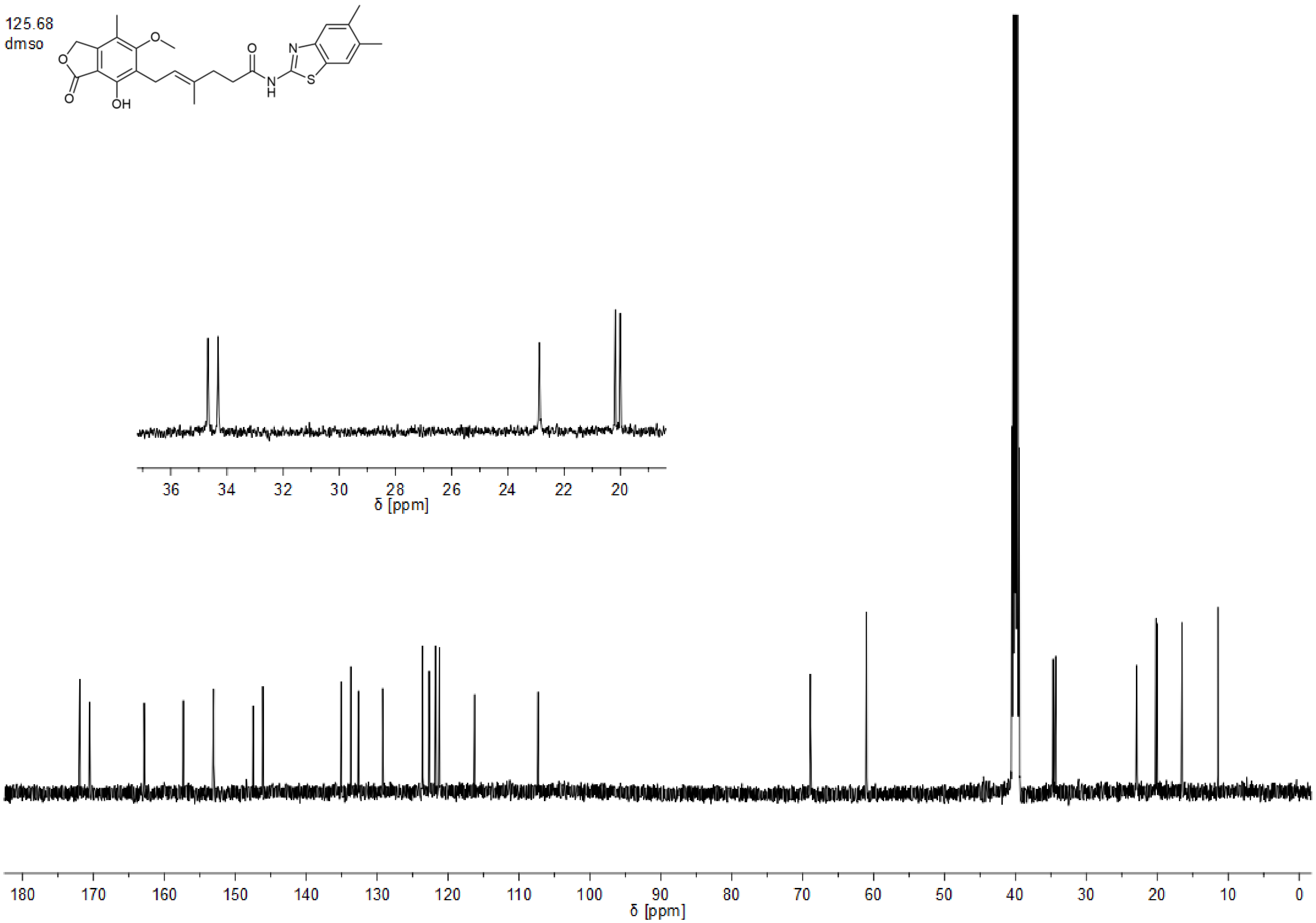
125.68
dmso
δ [ppm]
δ [ppm]

N-(6-fluorobenzo[*d*]thiazol-2-yl) mycophenolate (**A11**):

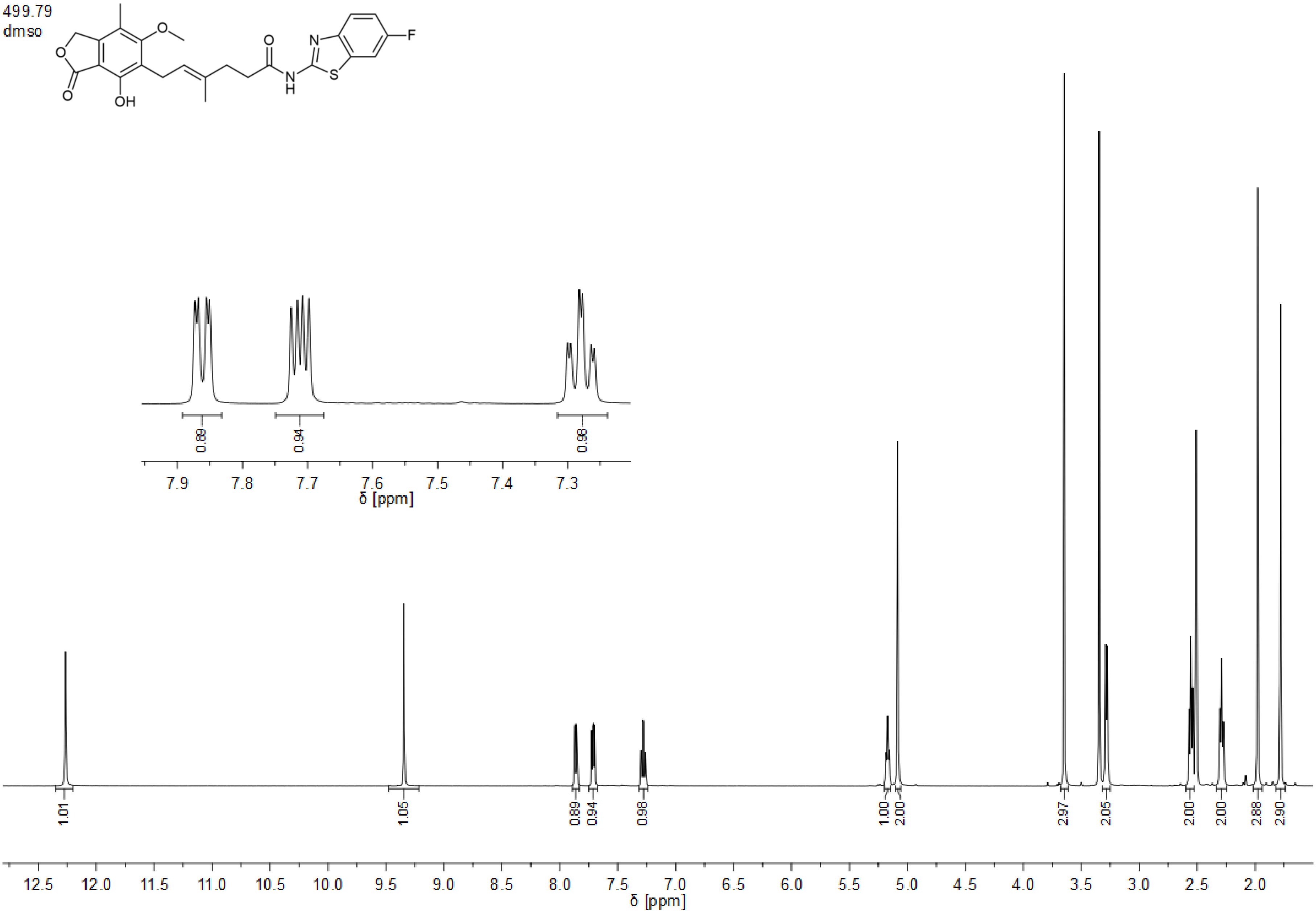

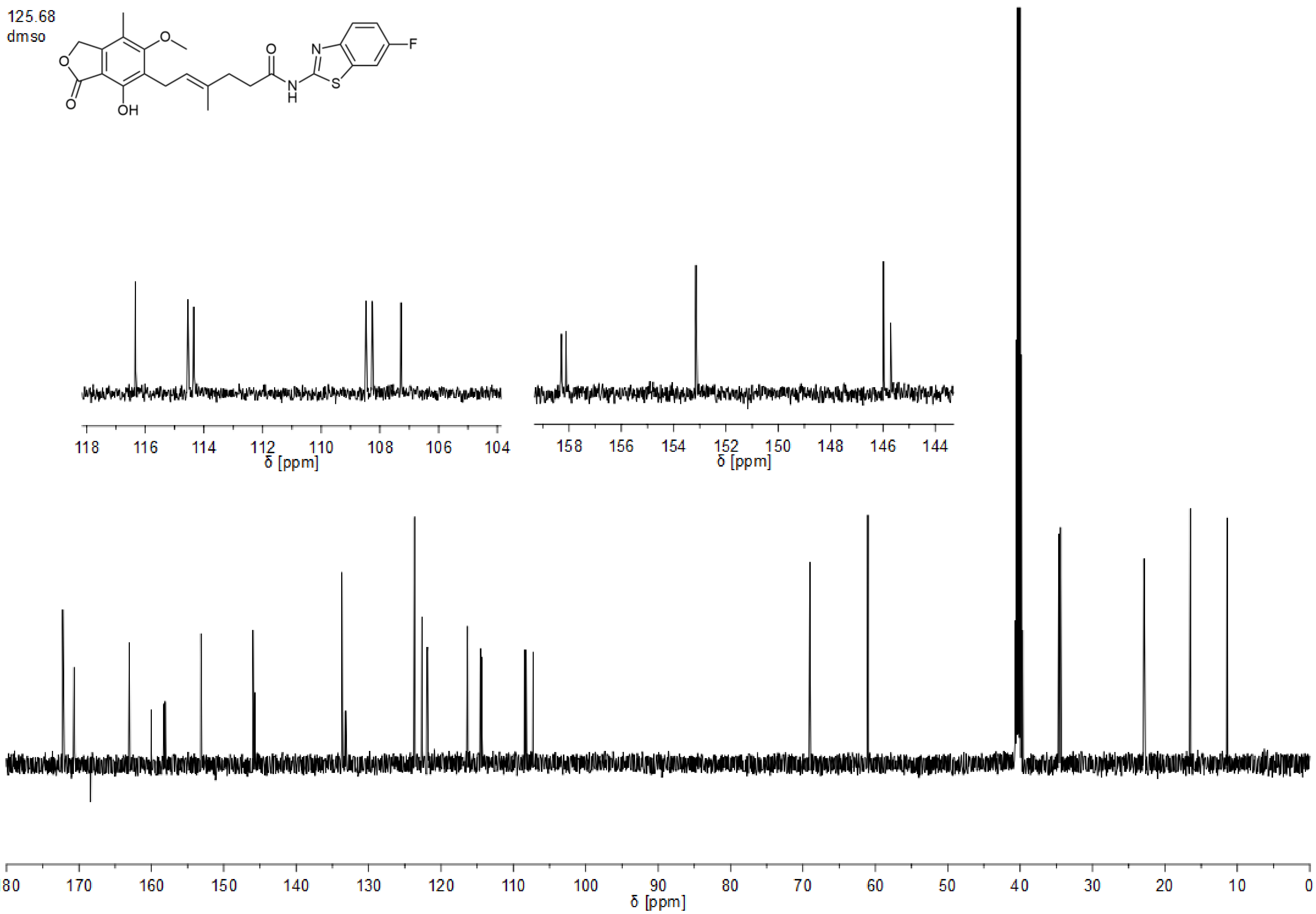
125.68
dmso
O
O
OH
O
O
N
H
N
S
F
118
116
114
112
110
108
106
104
δ [ppm]
158
156
154
152
150
148
146
144
δ [ppm]
180
170
160
150
140
130
120
110
100
90
80
70
60
50
40
30
20
10
0
δ [ppm]

N-(6-chlorobenzo[*d*]thiazol-2-yl) mycophenolate (**A12**):

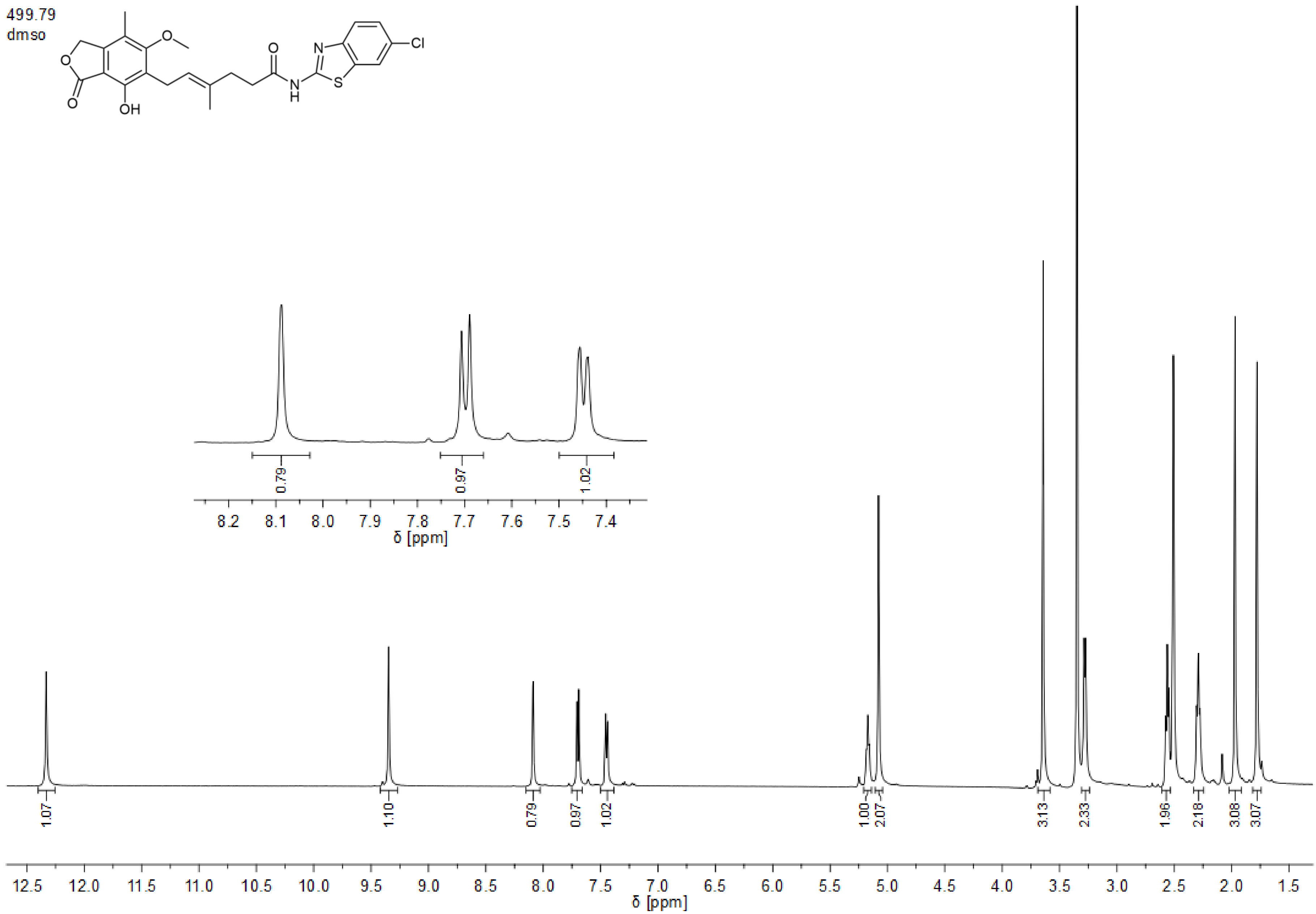

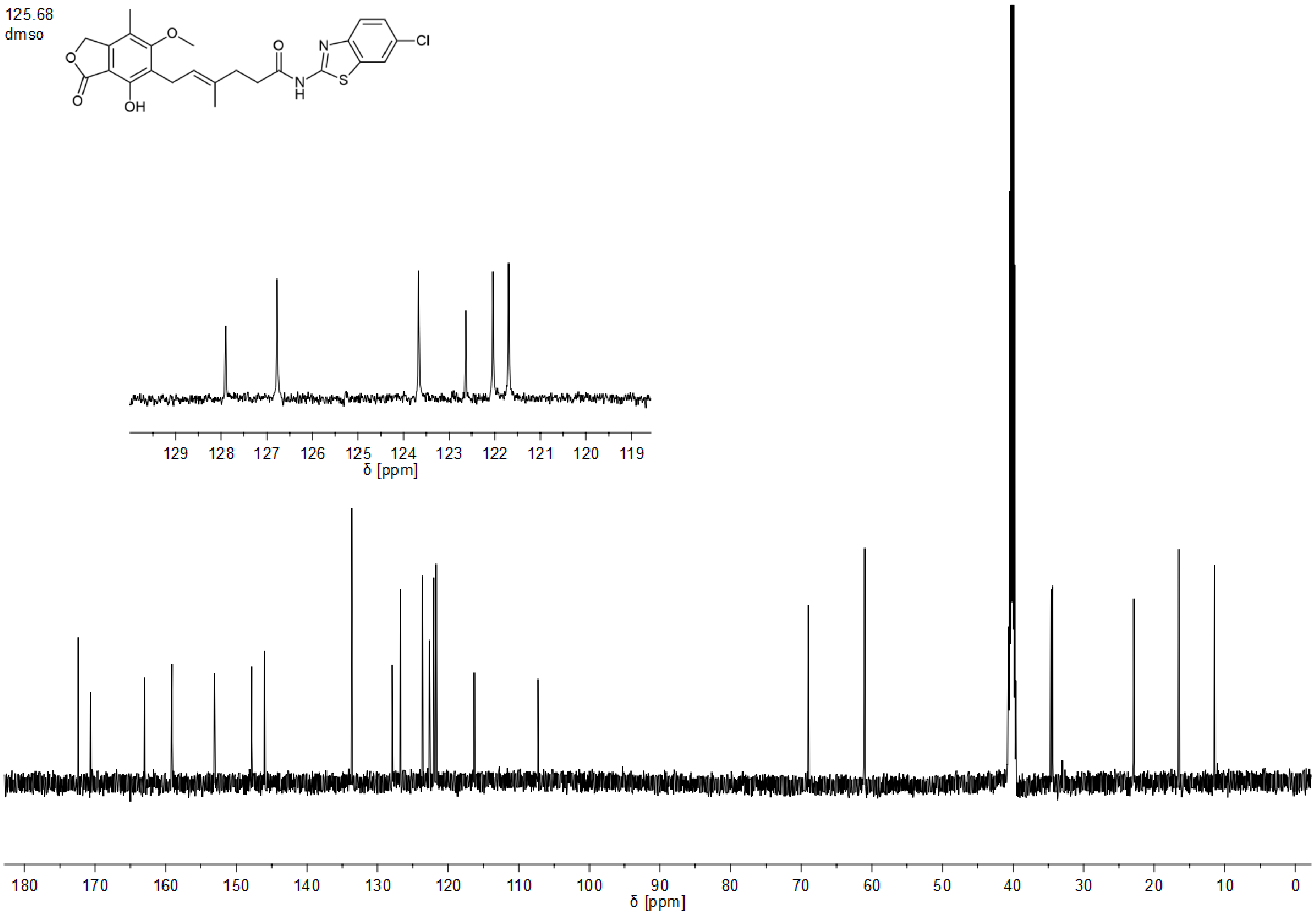
125.68
dmso
O
OH
N
H
S
Cl
129 128 127 126 125 124 123 122 121 120 119
δ [ppm]
180 170 160 150 140 130 120 110 100 90 80 70 60 50 40 30 20 10 0
δ [ppm]

N-(6-bromobenzo[*d*]thiazol-2-yl) mycophenolate (**A13**):

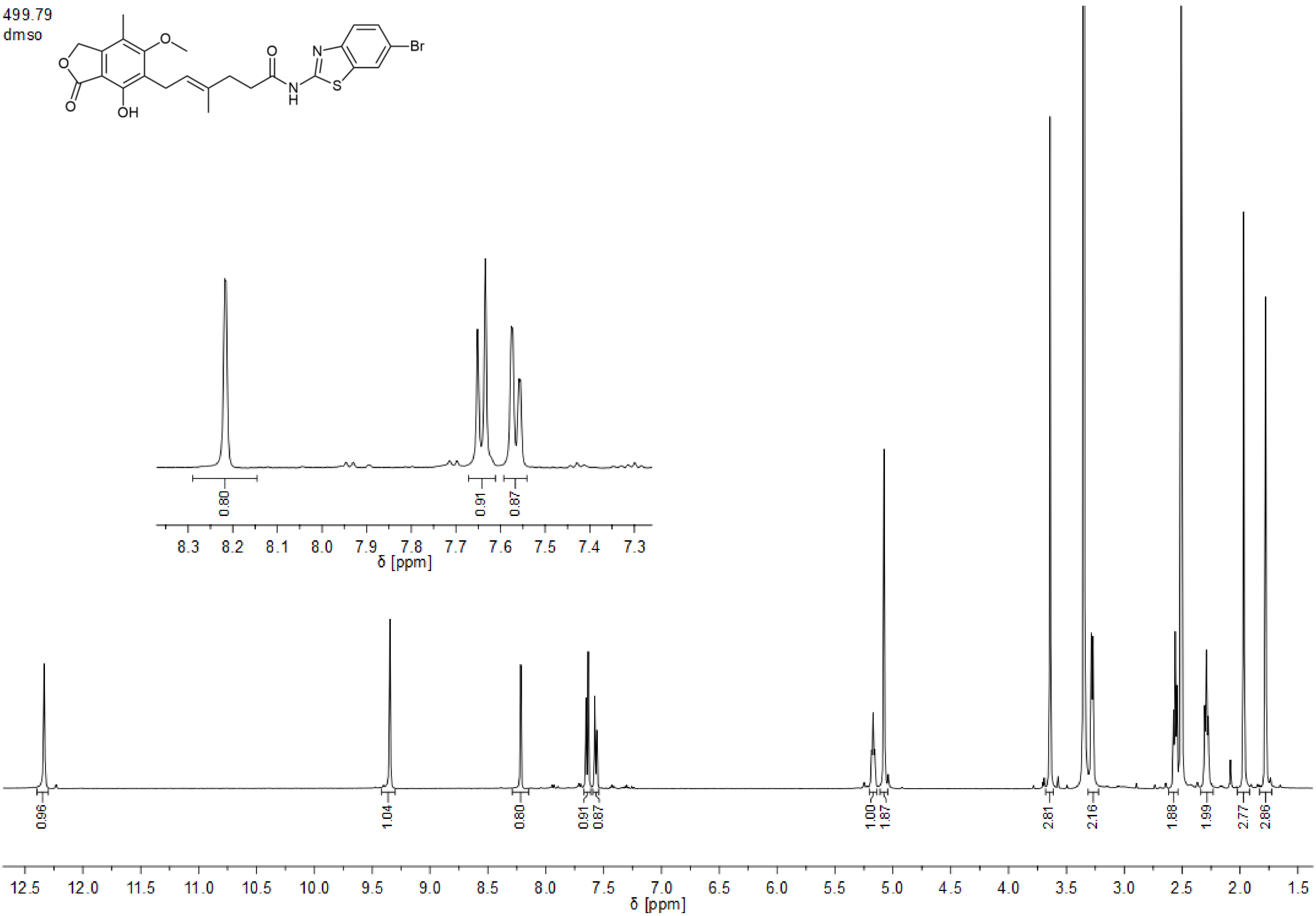

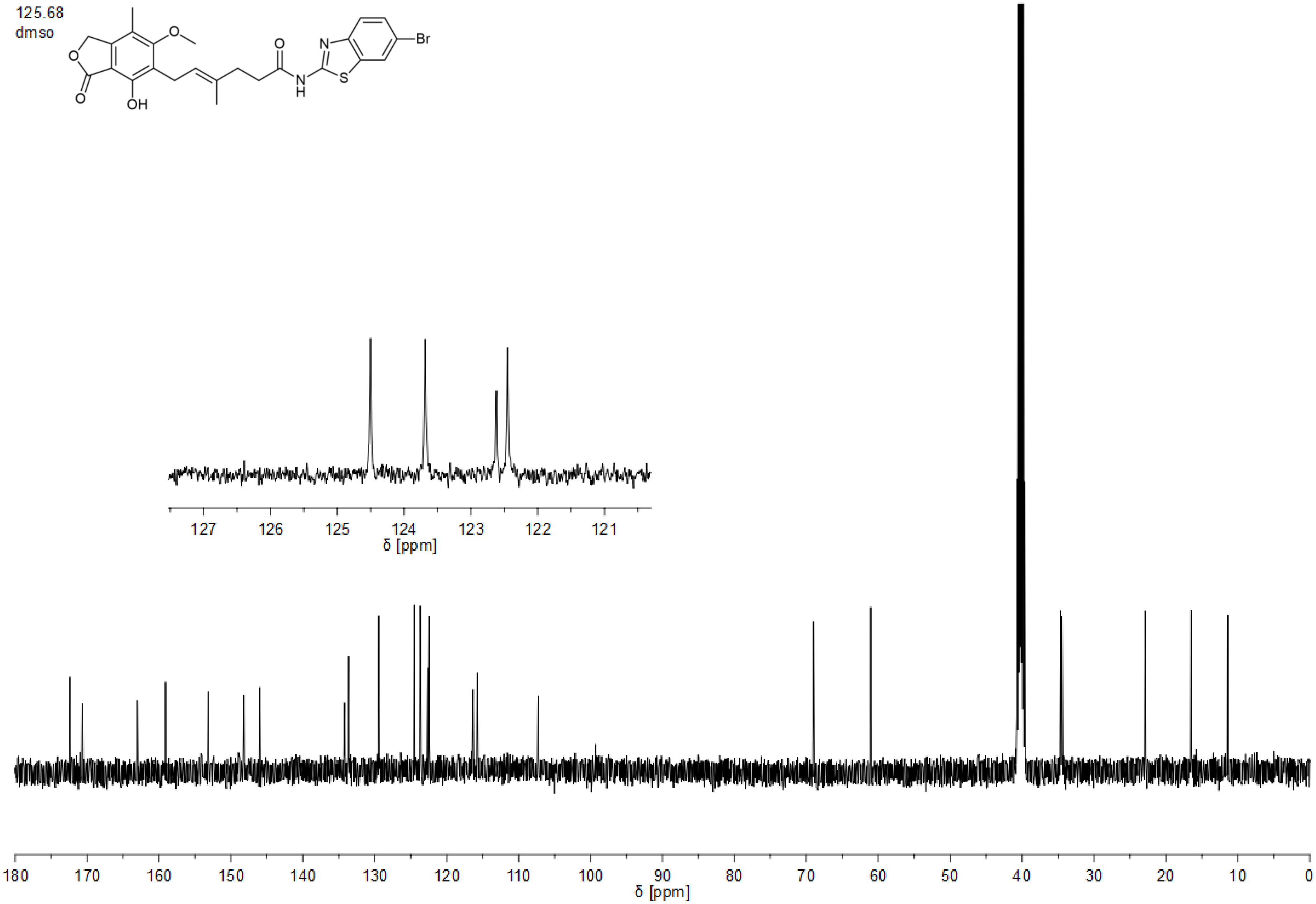
125.68
dmso
O
O
OH
O
N
H
N
S
Br
127 126 125 124 123 122 121
δ [ppm]
180 170 160 150 140 130 120 110 100 90 80 70 60 50 40 30 20 10 0
δ [ppm]

N-[6-(trifluoromethyl)benzo[*d*]thiazol-2-yl] mycophenolate (**A14**):

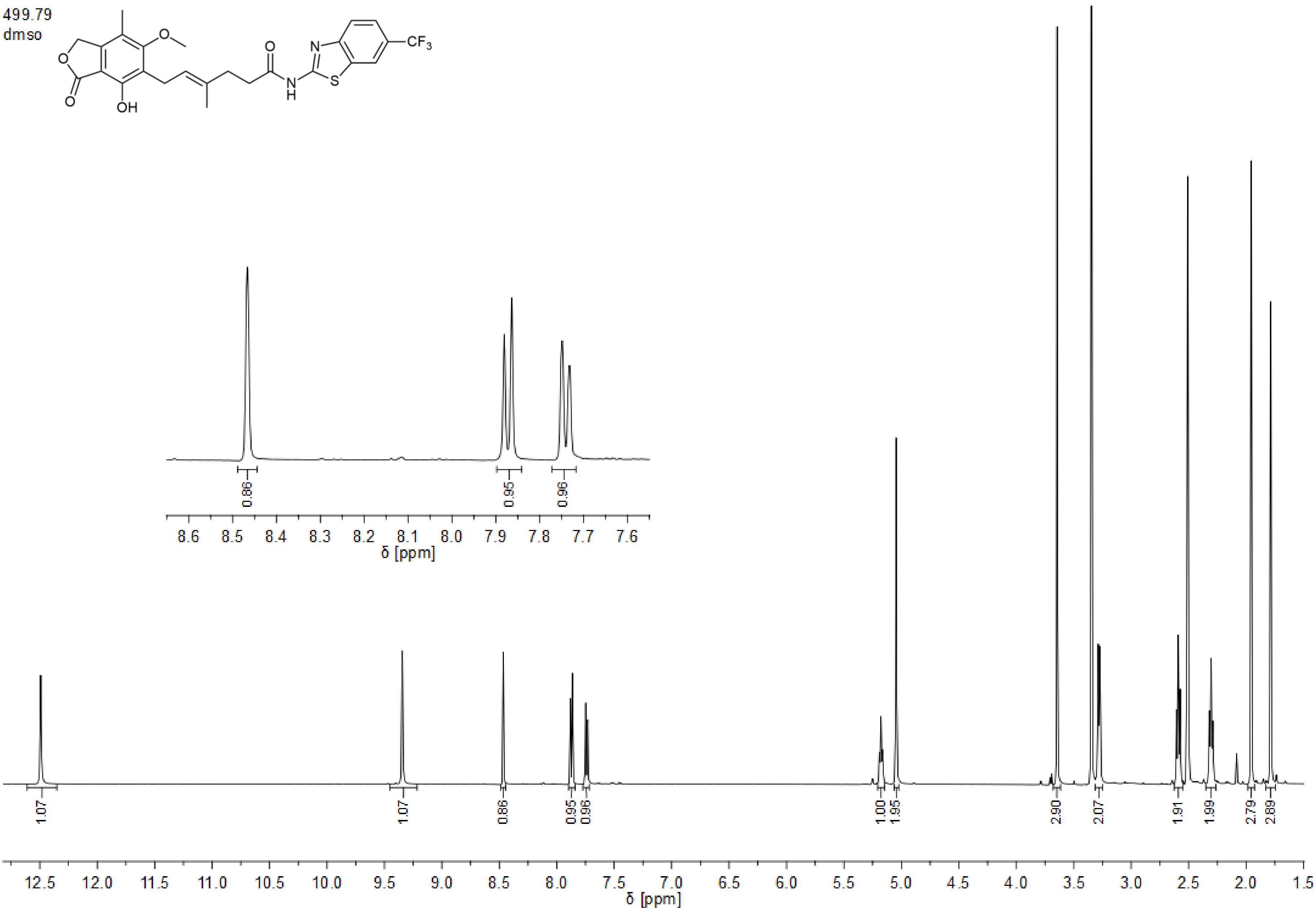

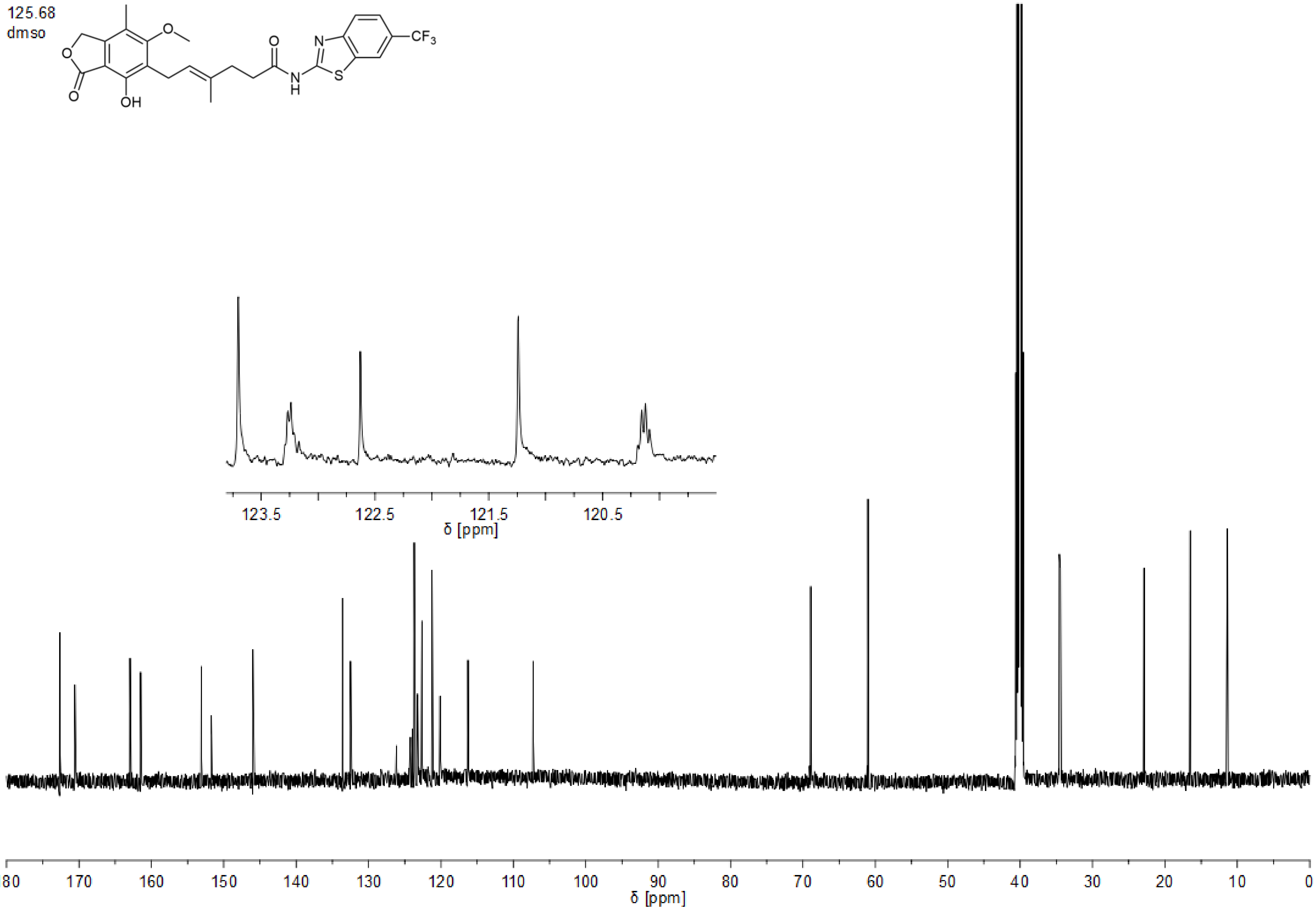
125.68
dmso
δ [ppm]

N-(6-nitrobenzo[*d*]thiazol-2-yl) mycophenolate (**A15**):

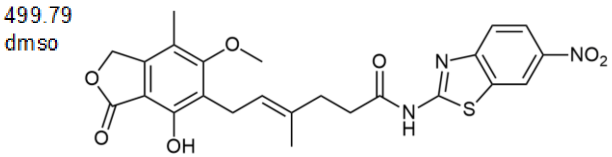

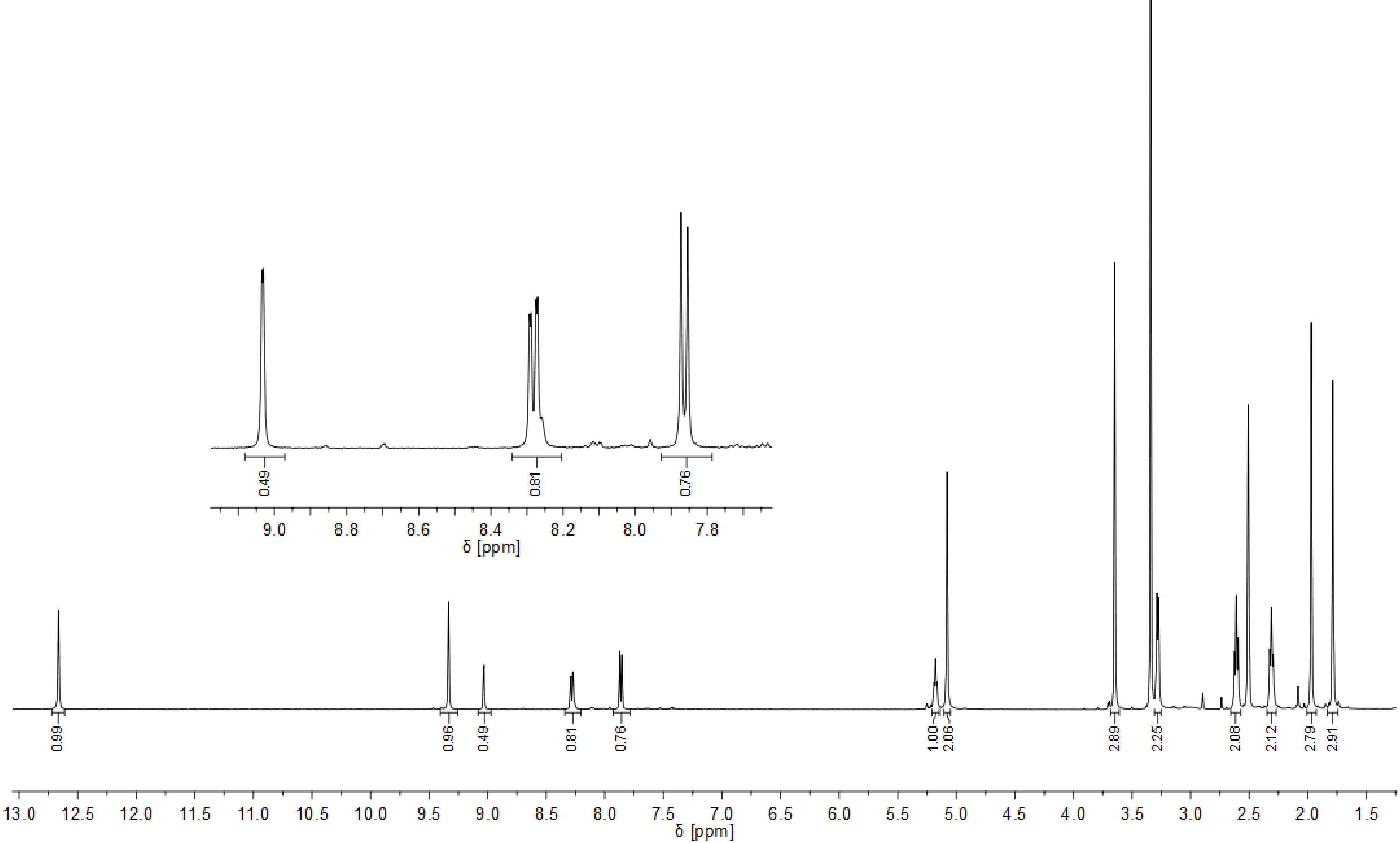

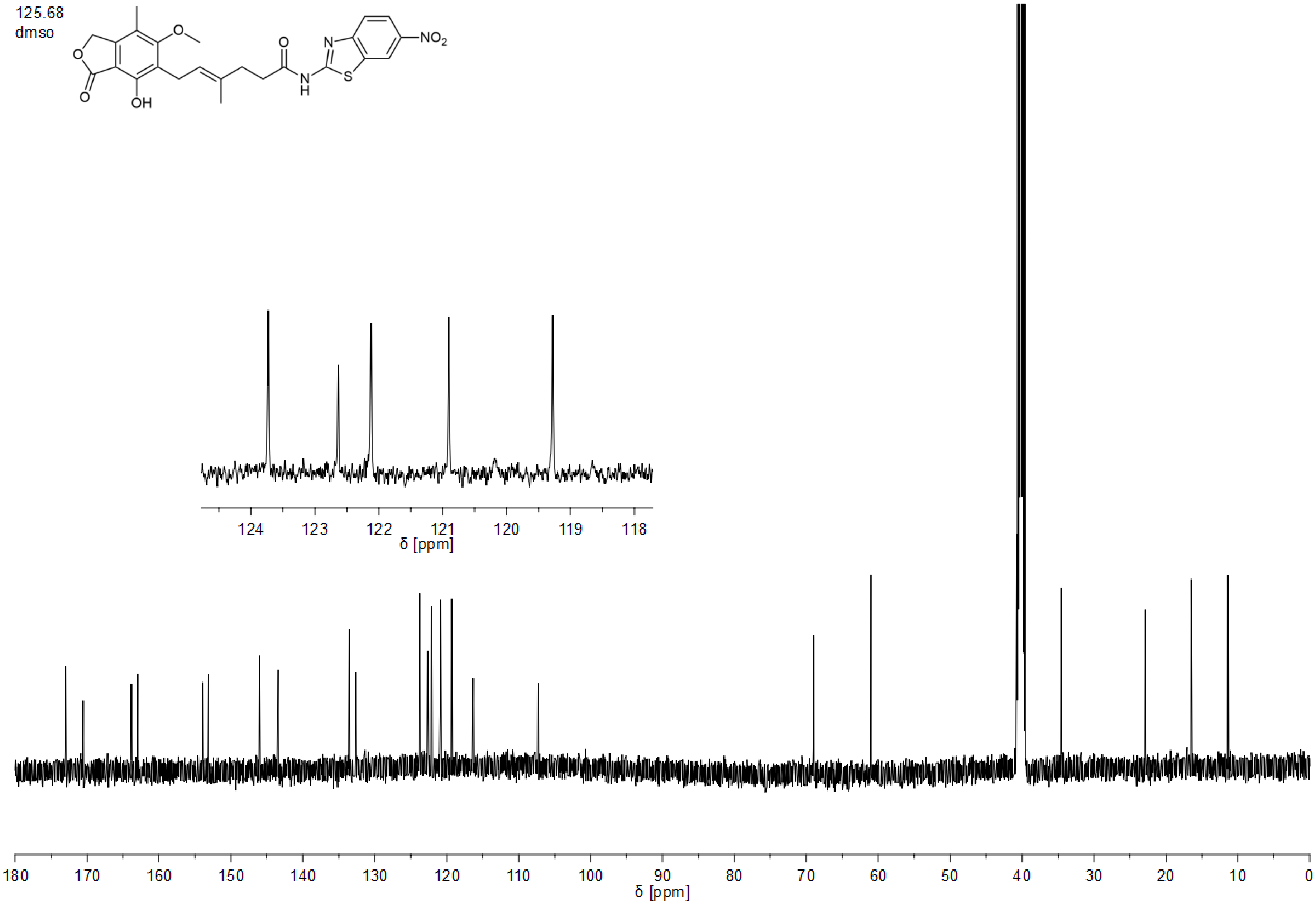
125.68
dmso
O
OH
N
H
S
NO_2
124 123 122 121 120 119 118
δ [ppm]
180 170 160 150 140 130 120 110 100 90 80 70 60 50 40 30 20 10 0
δ [ppm]

N-(4-methoxybenzo[*d*]thiazol-2-yl) mycophenolate (**A16**):

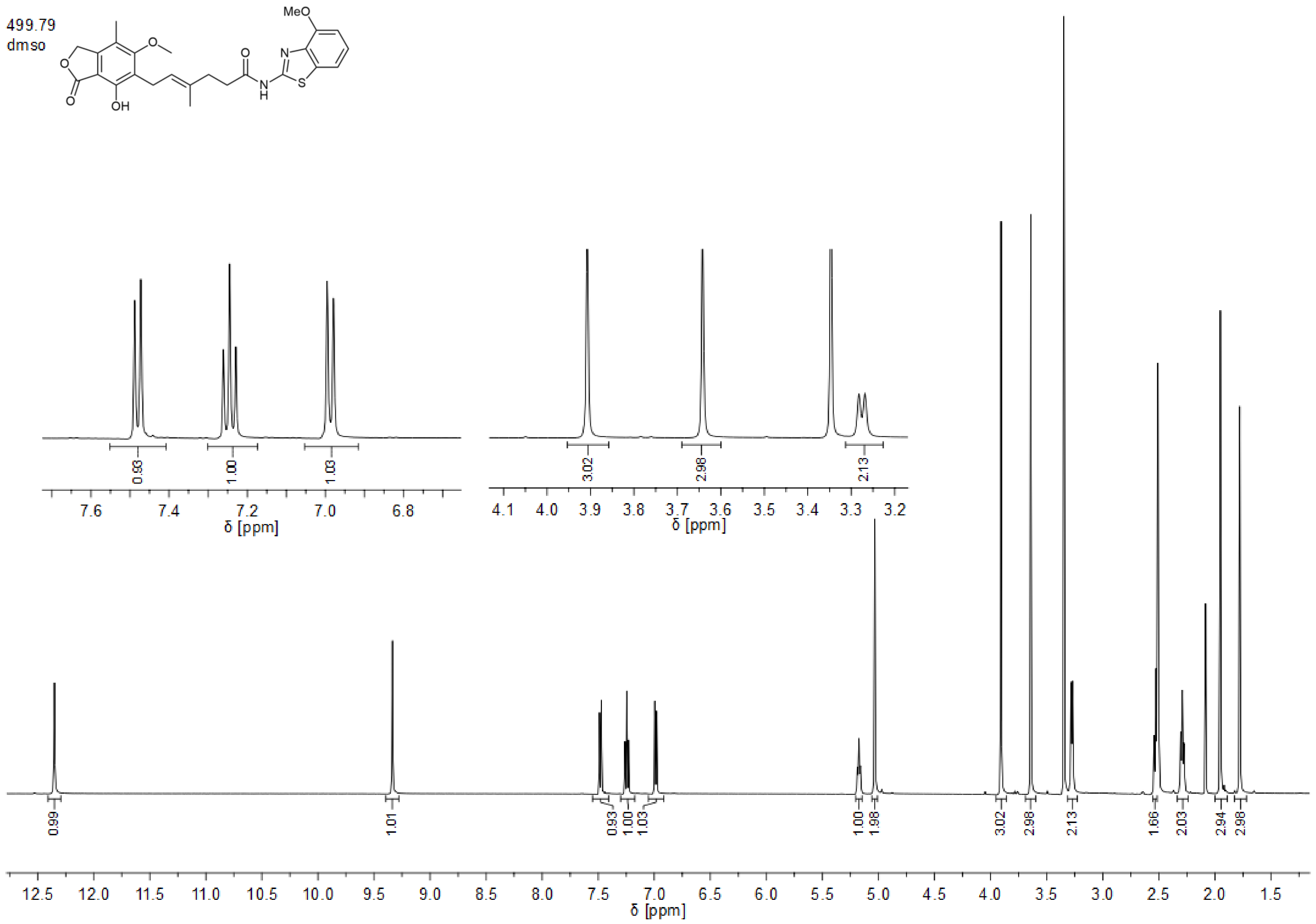

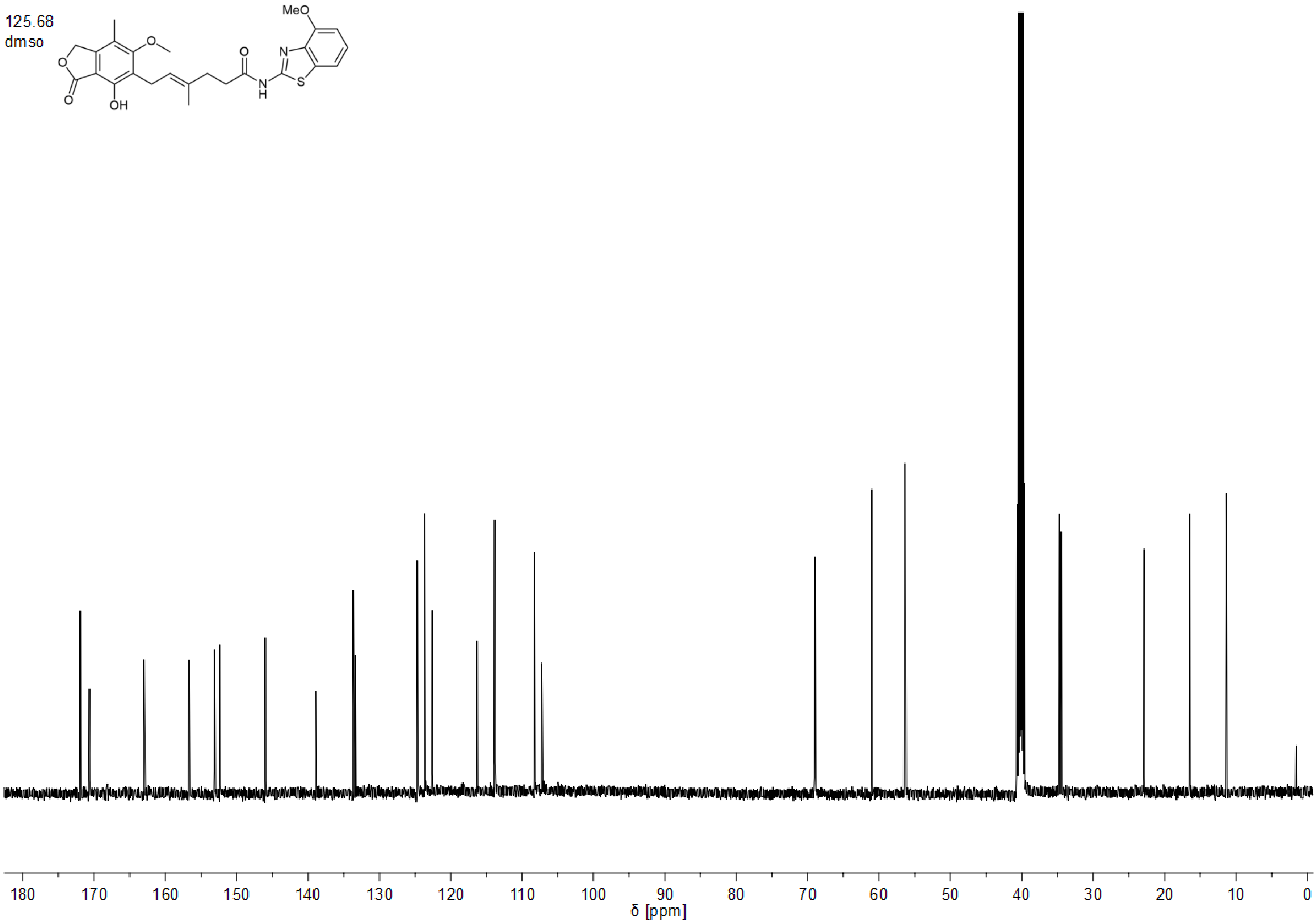
125.68
dmso
MeO
N
S
O
N
H
O
O
OH
O
180 170 160 150 140 130 120 110 100 90 80 70 60 50 40 30 20 10 0
δ [ppm]

N-(4-methylbenzo[*d*]thiazol-2-yl) mycophenolate (**A17**):

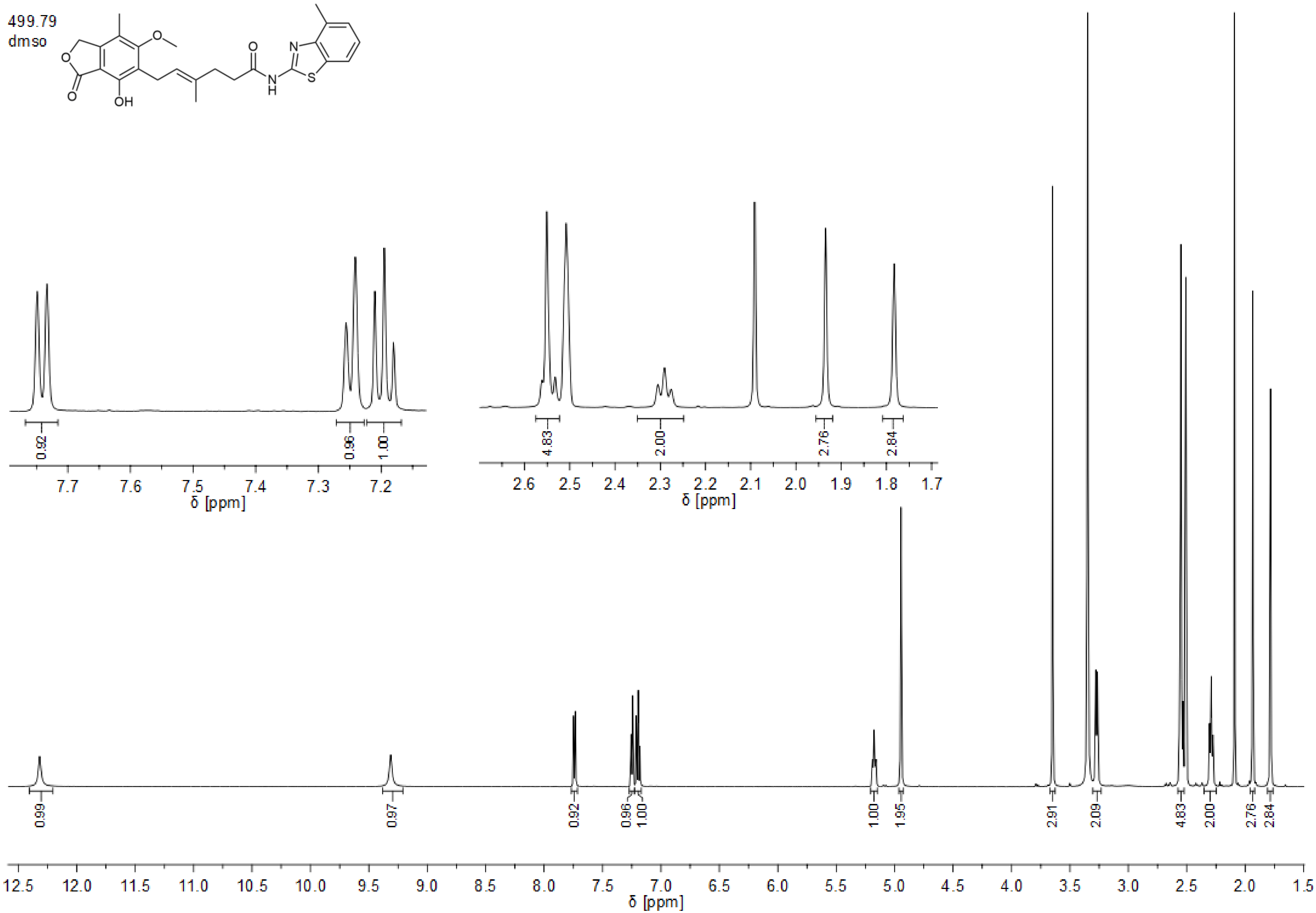

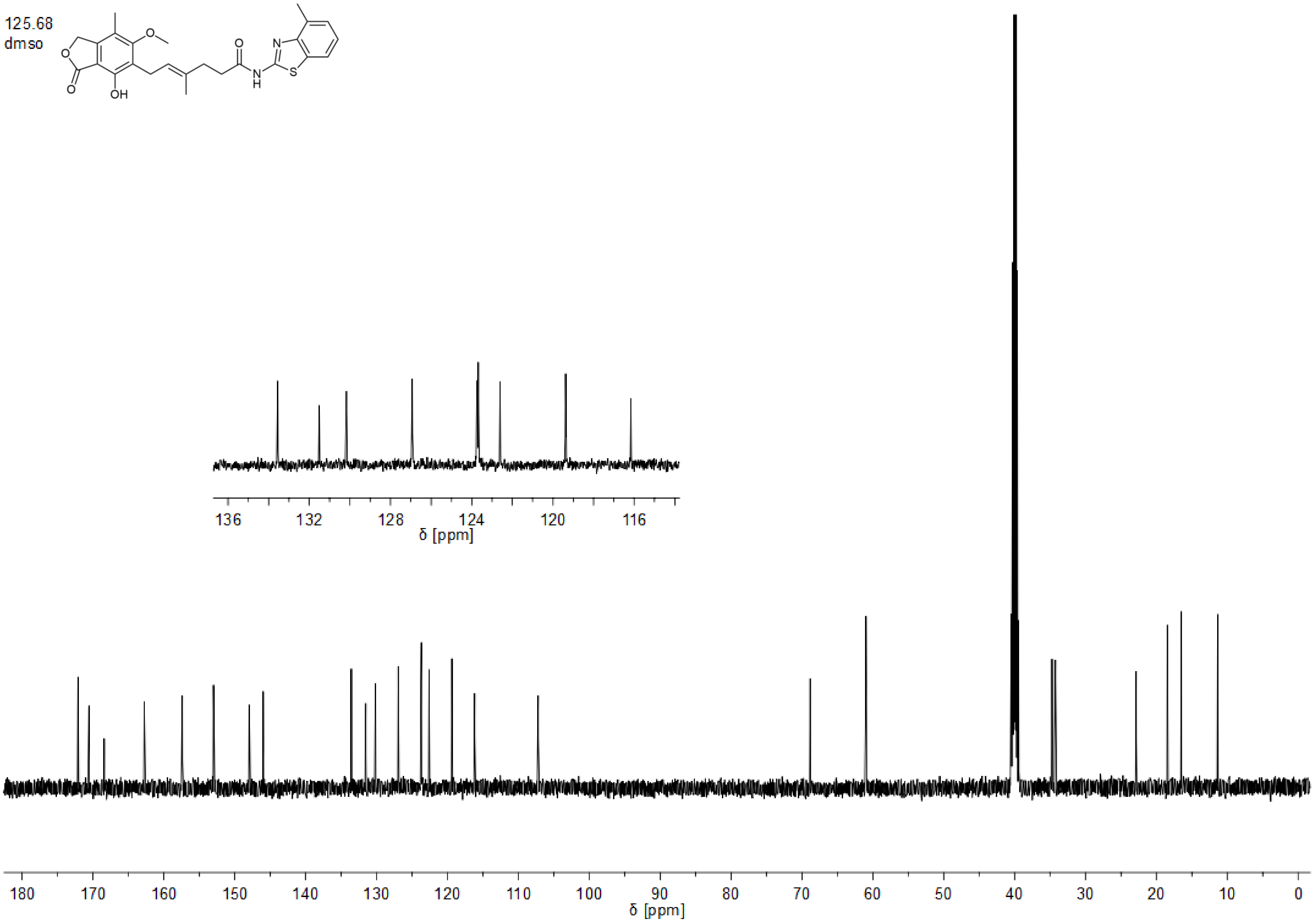
125.68
dmso
O
O
OH
O
O
N
H
N
S
136
132
128
124
120
116
δ [ppm]
180
170
160
150
140
130
120
110
100
90
80
70
60
50
40
30
20
10
0
δ [ppm]

N-(4-chlorobenzo[*d*]thiazol-2-yl) mycophenolate (**A18**):

499.79
dmso

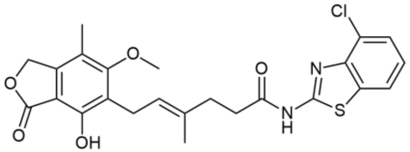

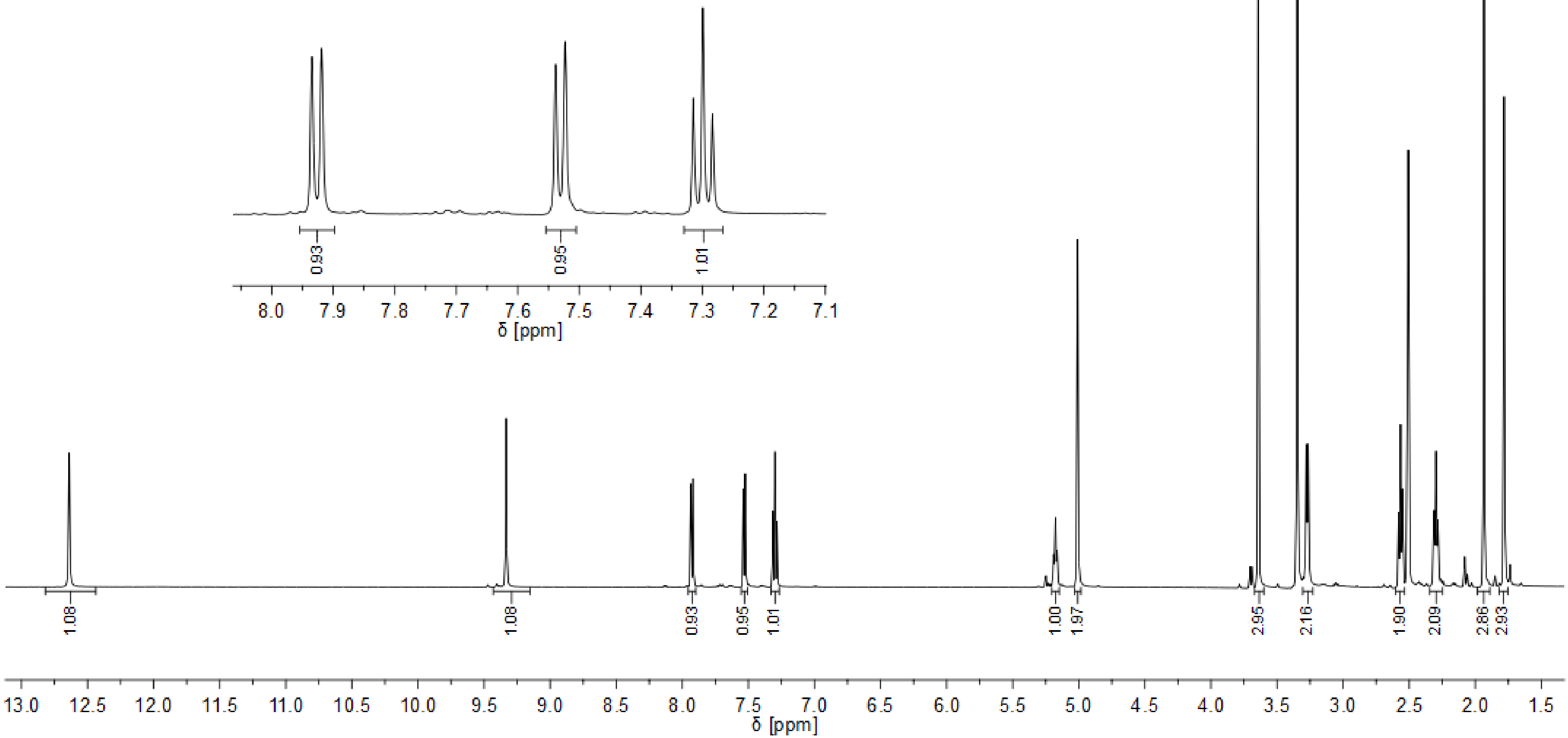

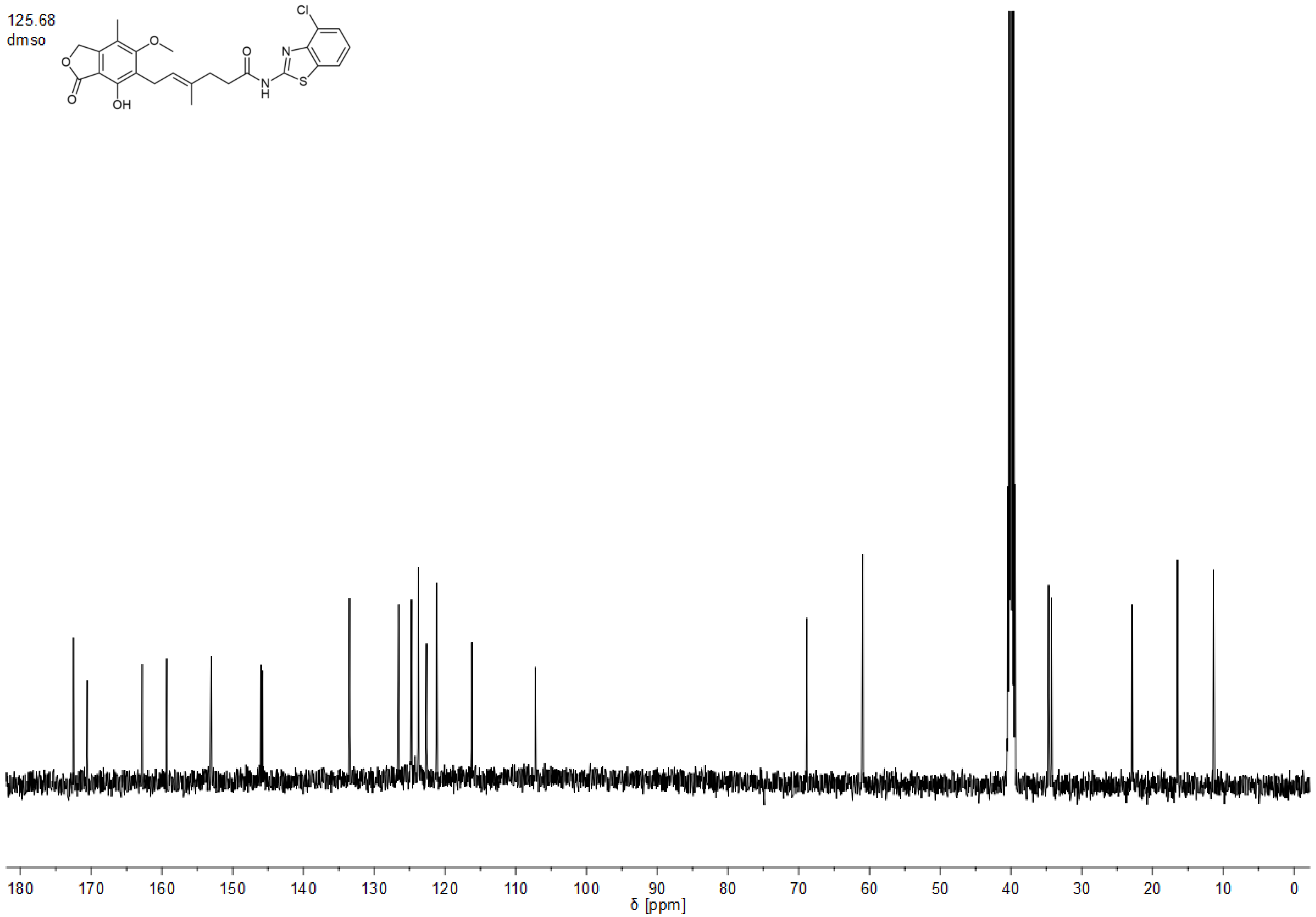
125.68
dmso
Cl
N
S
O
OH
H
180 170 160 150 140 130 120 110 100 90 80 70 60 50 40 30 20 10 0
δ [ppm]

Mycophenolic acid (**MPA**):

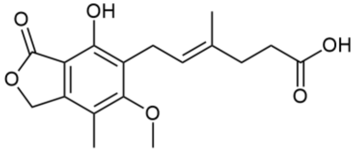

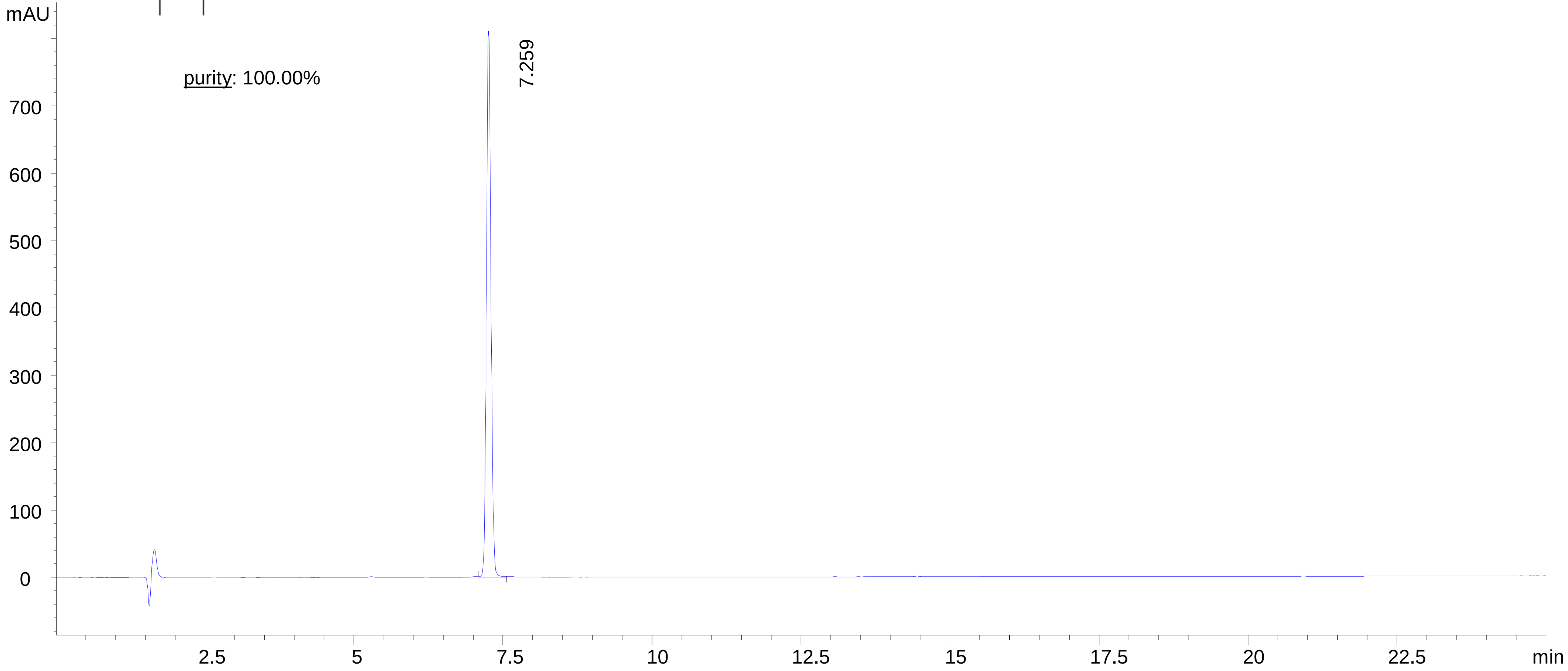

N-(benzo[*d*]thiazol-2-yl) mycophenolate (**A1**):

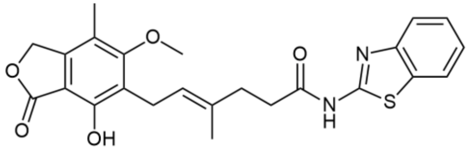

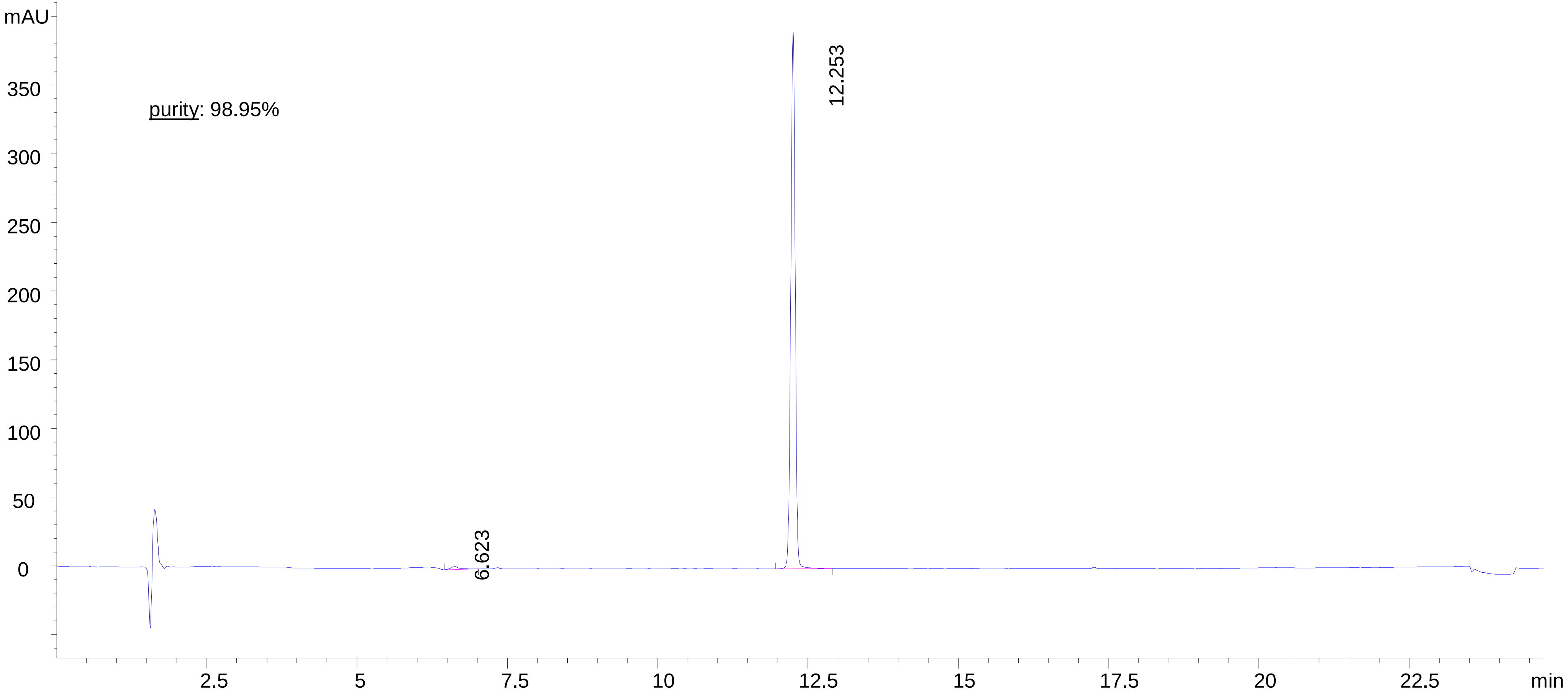

N-(benzo[*d*]oxazol-2-yl) mycophenolate (**A2**):

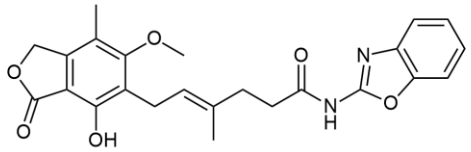

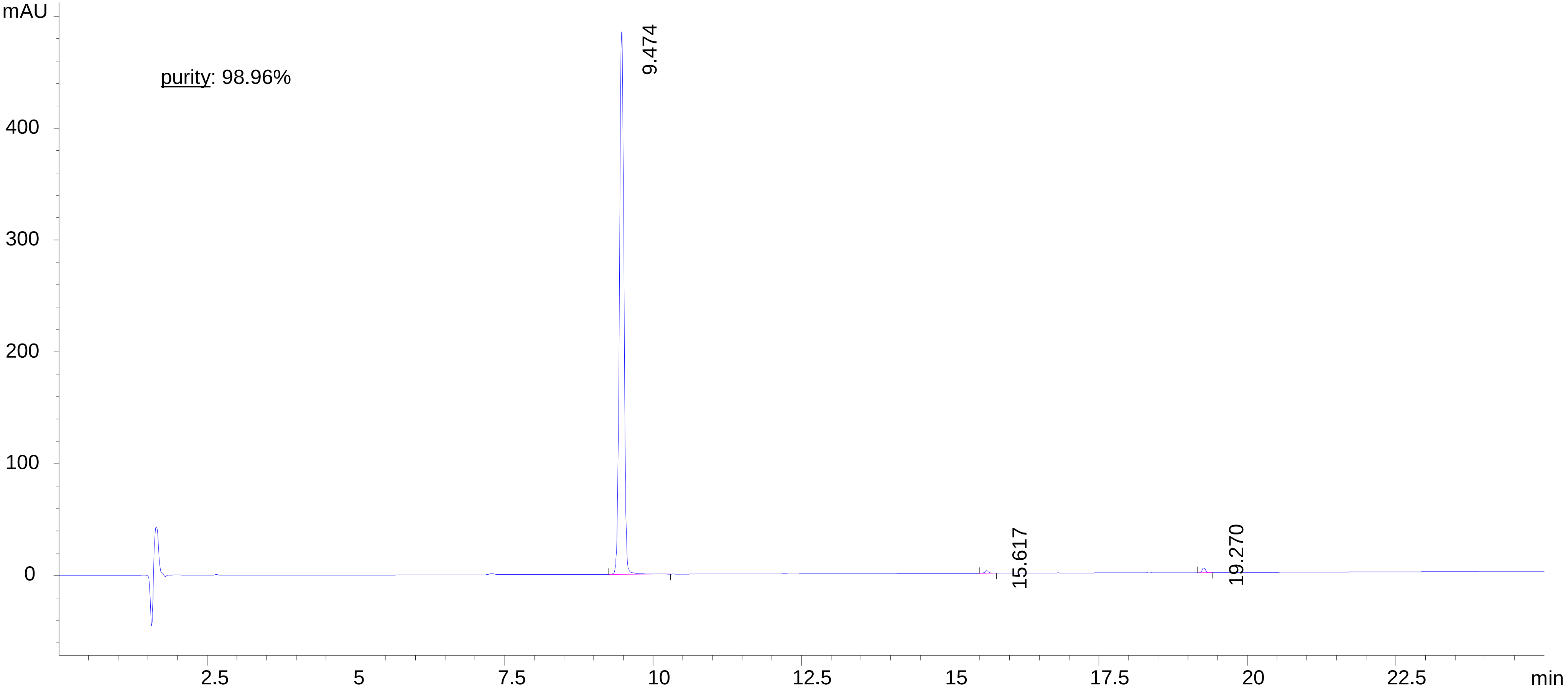

N-(1*H*-benzo[*d*]imidazol-2-yl) mycophenolate (**A3**):

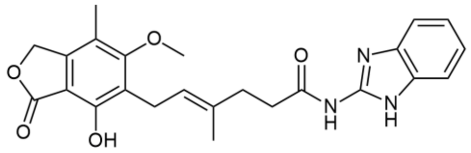

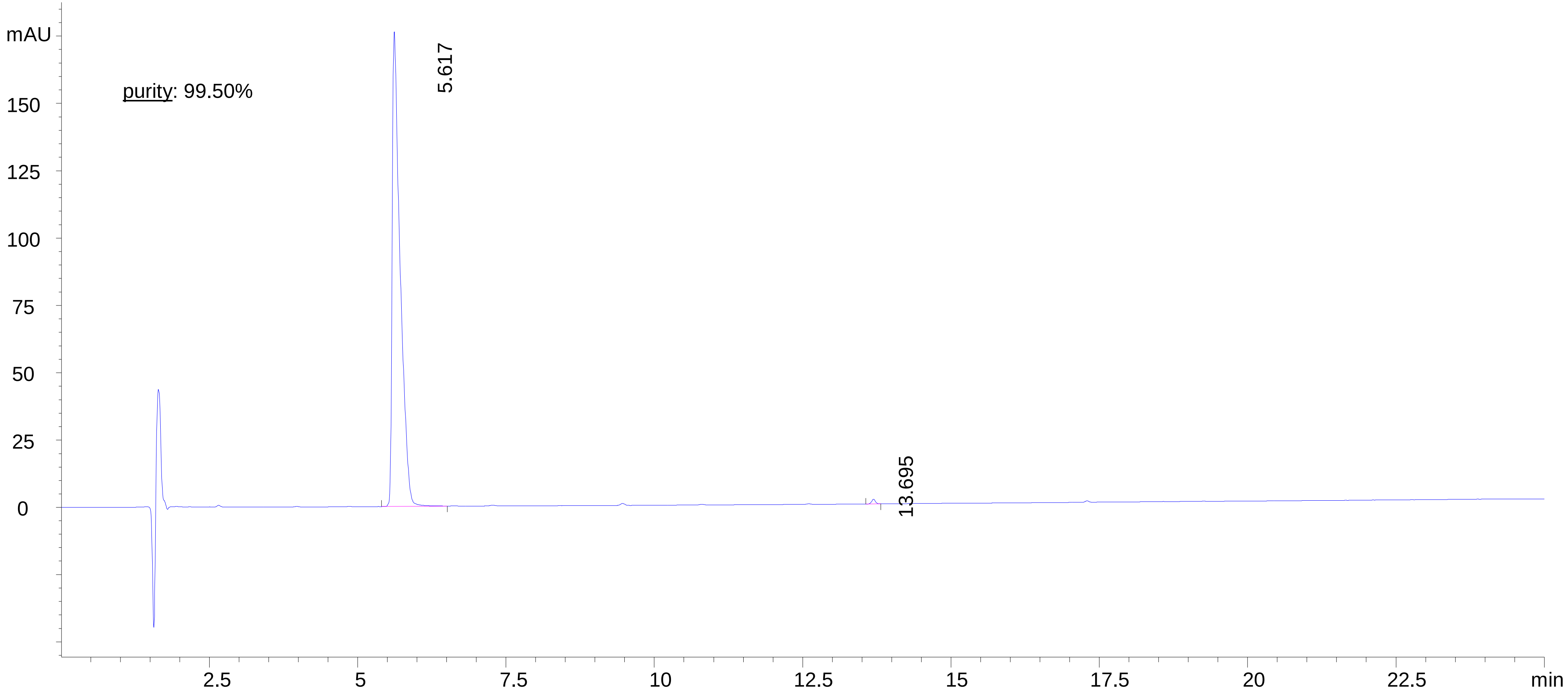

N-(benzo[*d*]thiazol-6-yl) mycophenolate (**A4**):

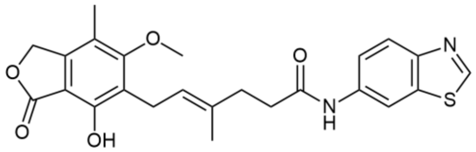

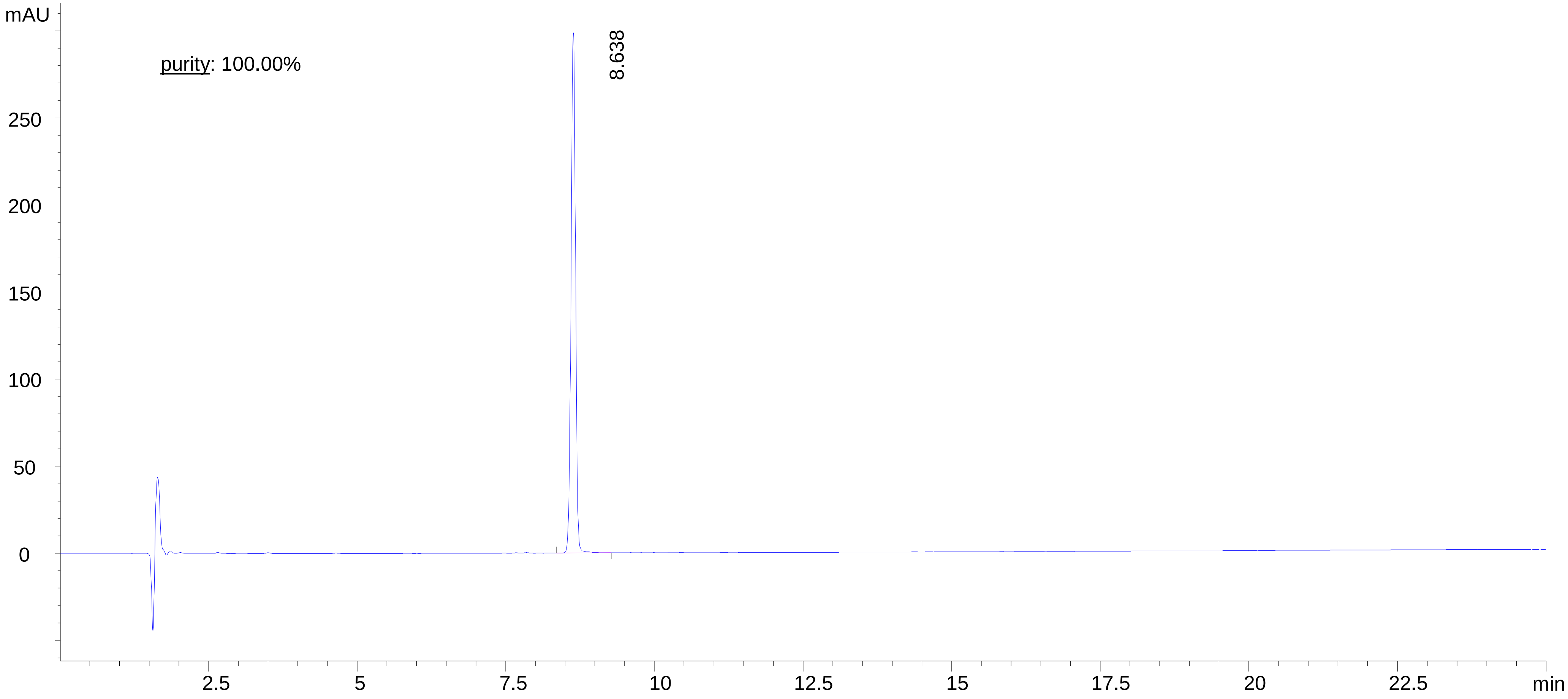

N-(benzo[*d*]thiazol-5-yl) mycophenolate (**A5**):

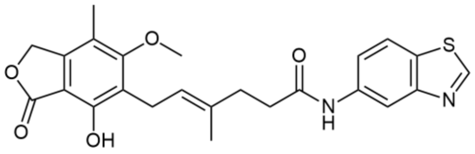

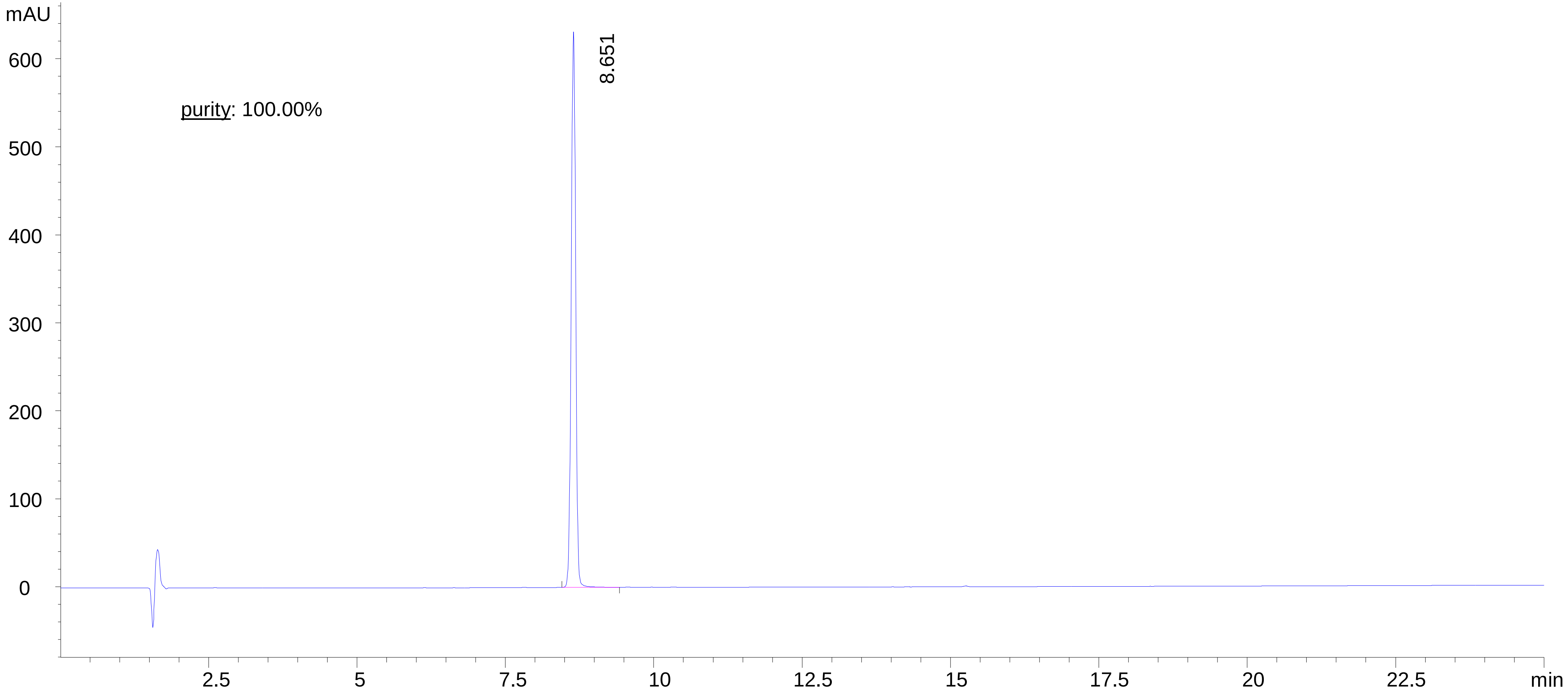

N-(benzo[*d*]oxazol-5-yl) mycophenolate (**A6**):

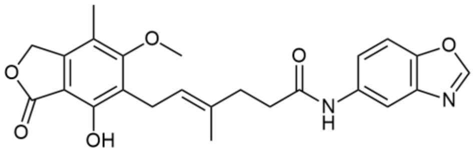

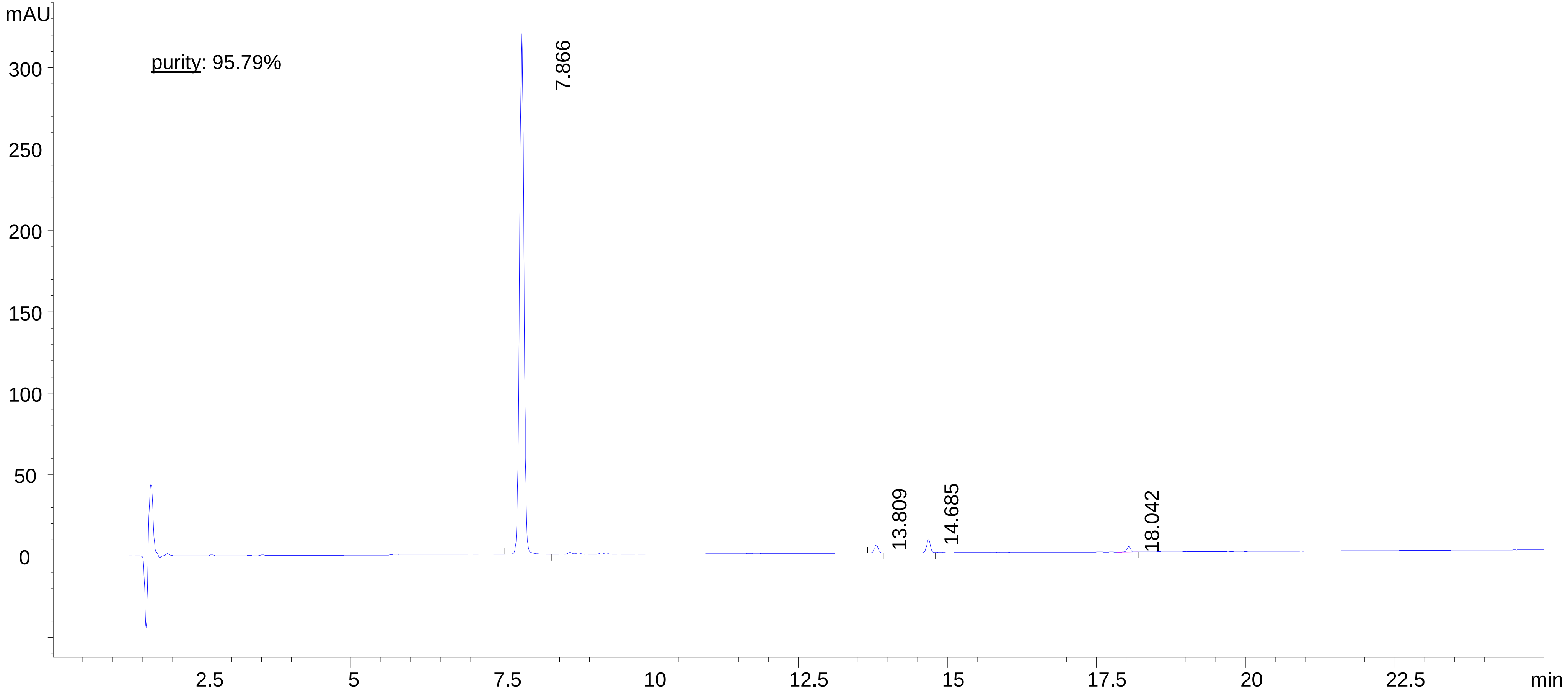

N-[1-(pirymidin-2-yl)methyl] mycophenolate (**A7**):

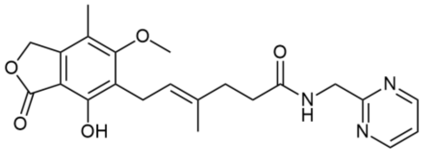

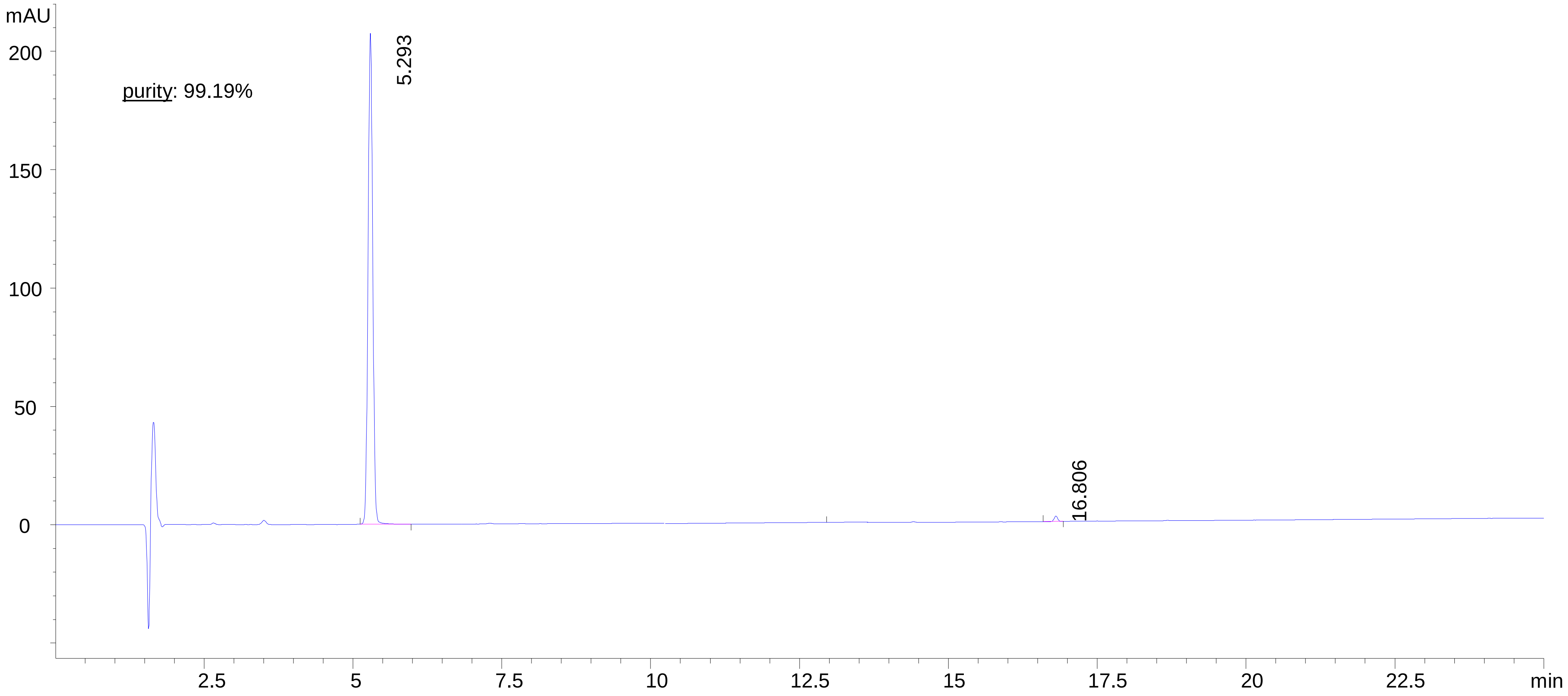

N-(6-methoxybenzo[*d*]thiazol-2-yl) mycophenolate (**A8**):

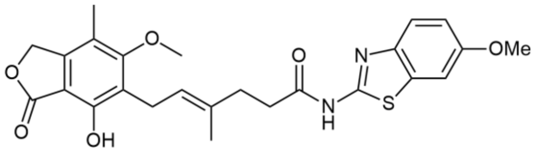

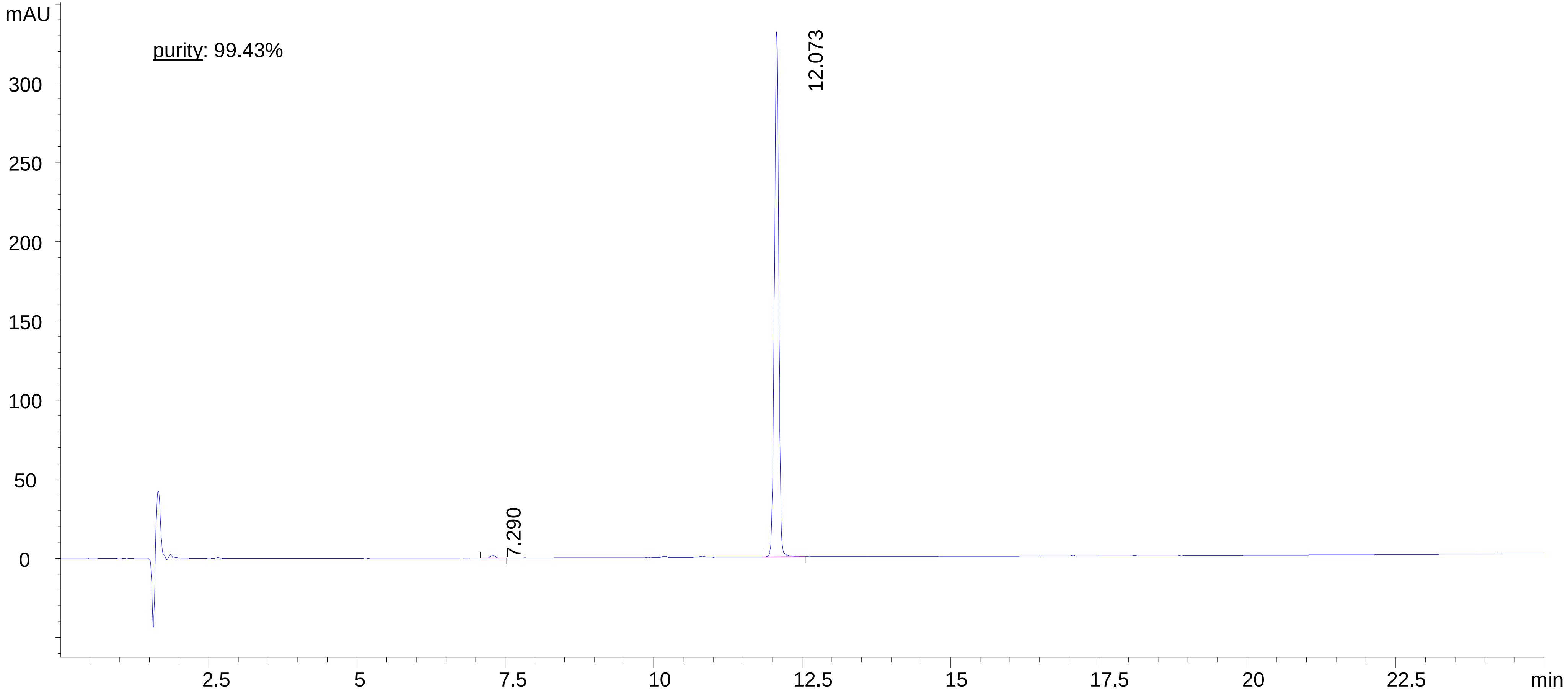

N-(6-methylbenzo[*d*]thiazol-2-yl) mycophenolate (**A9**):

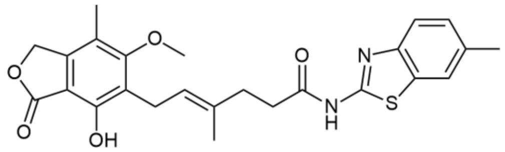

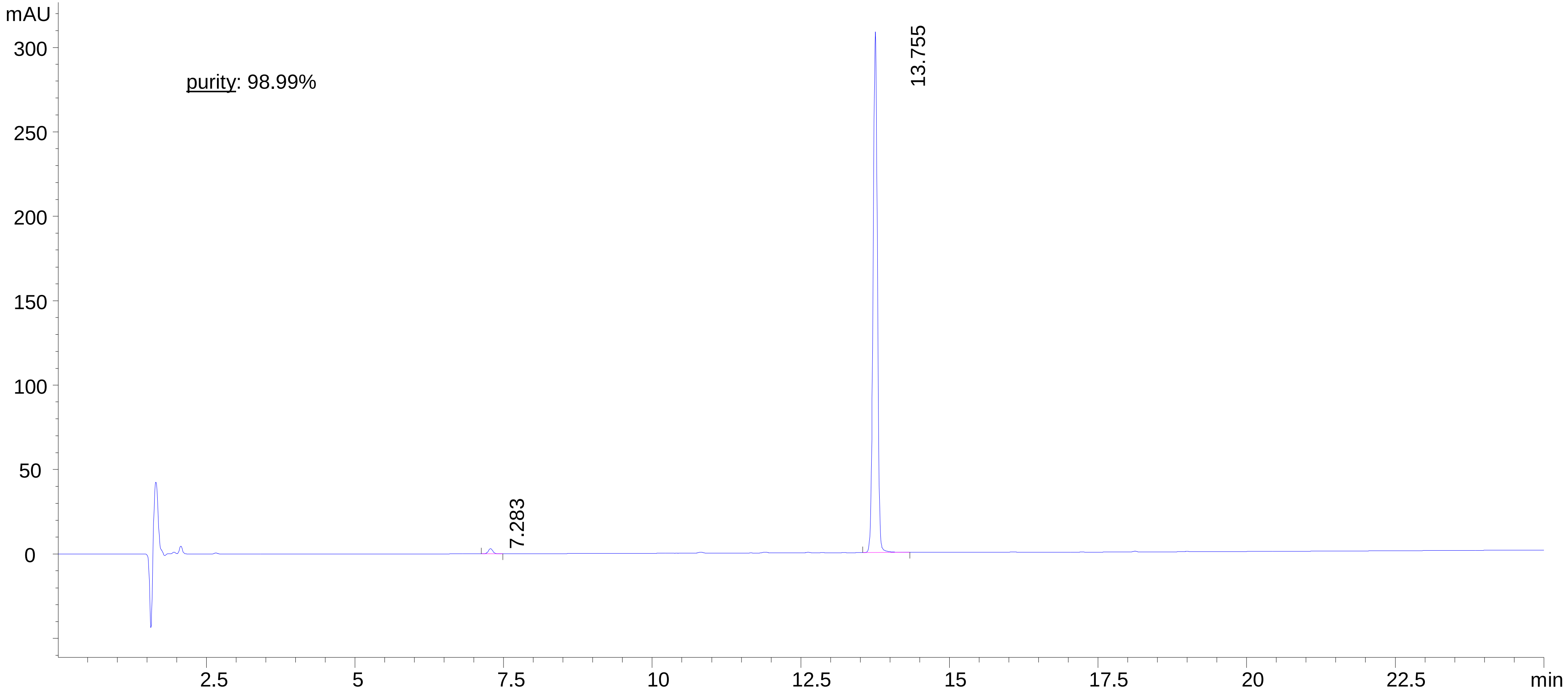

N-(5,6-dimethylbenzo[*d*]thiazol-2-yl) mycophenolate (**A10**):

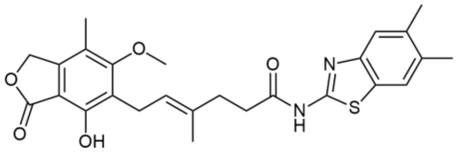

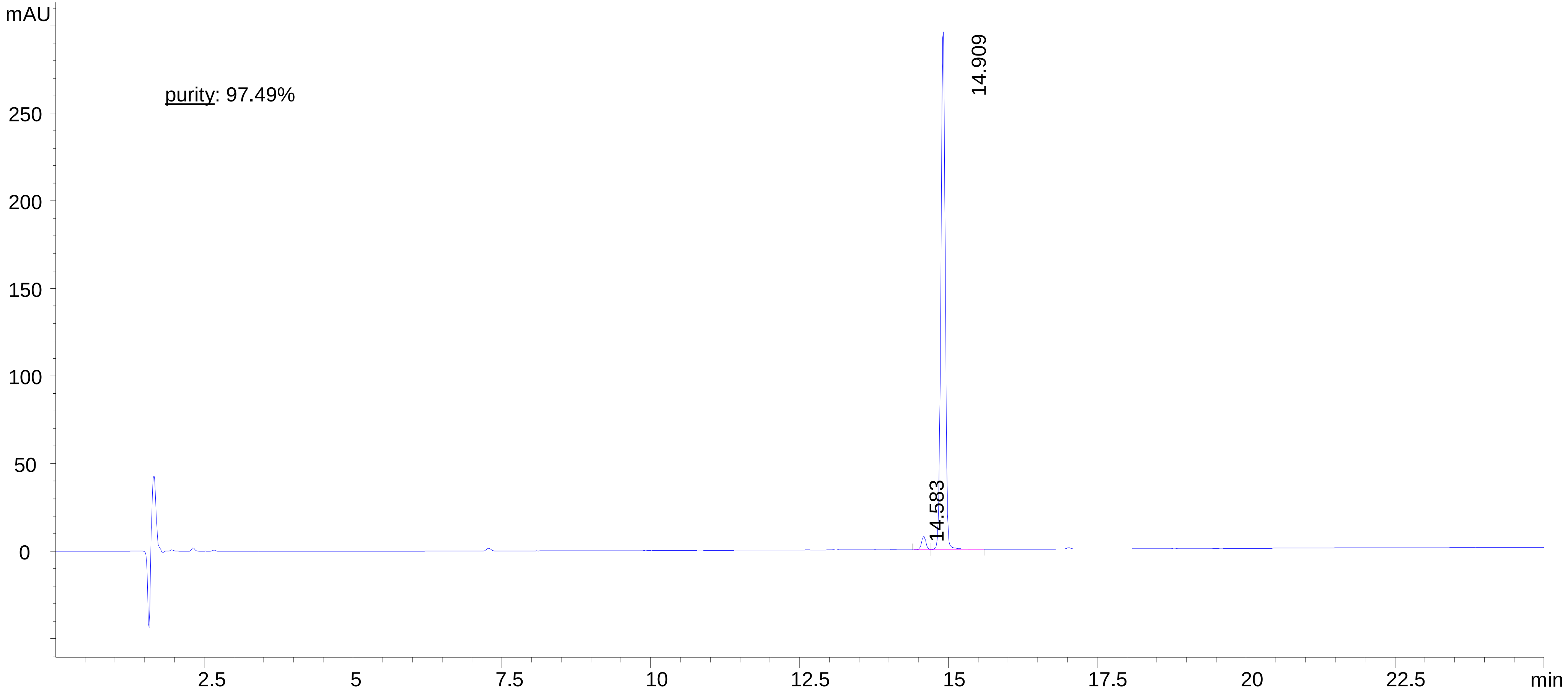

N-(6-fluorobenzo[*d*]thiazol-2-yl) mycophenolate (**A11**):

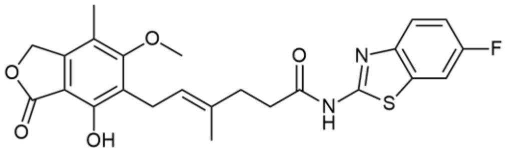

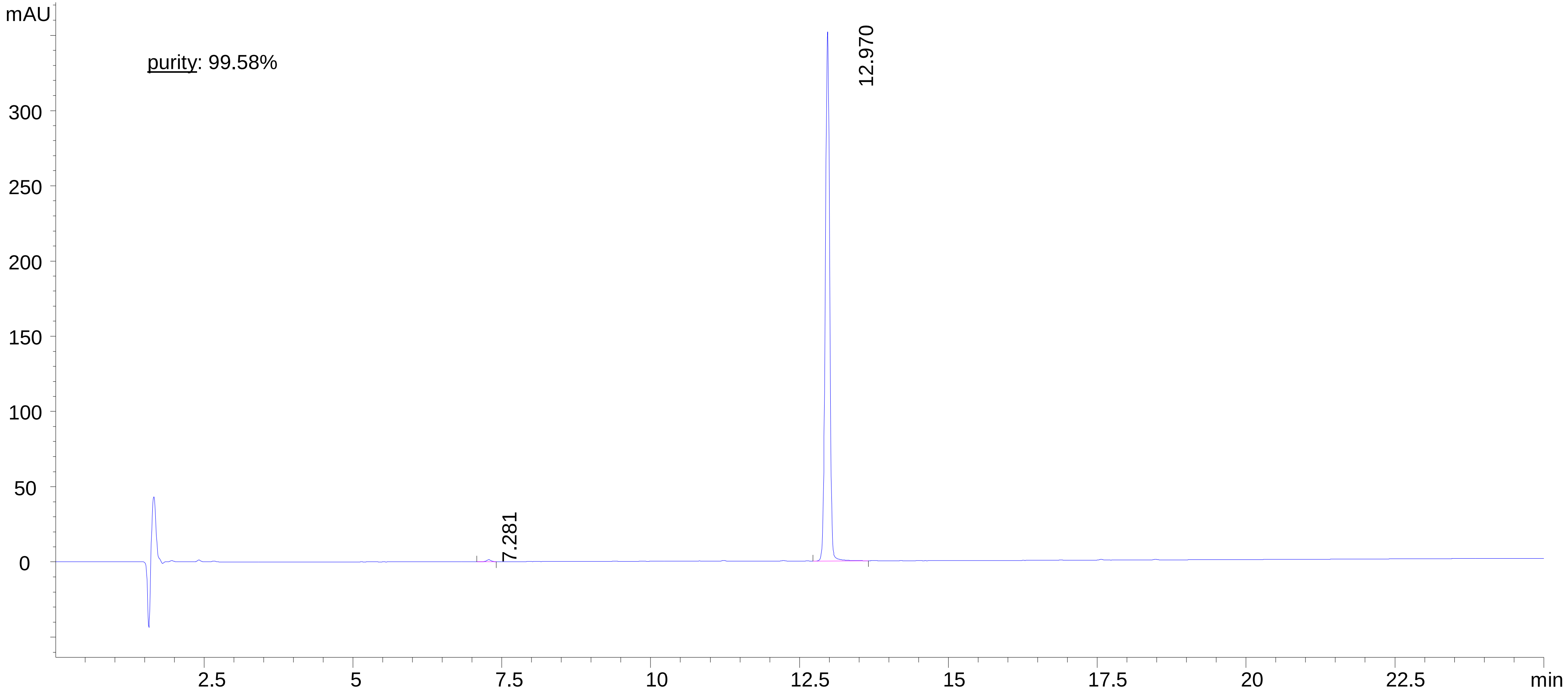

N-(6-chlorobenzo[*d*]thiazol-2-yl) mycophenolate (**A12**):

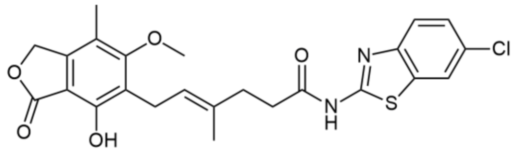

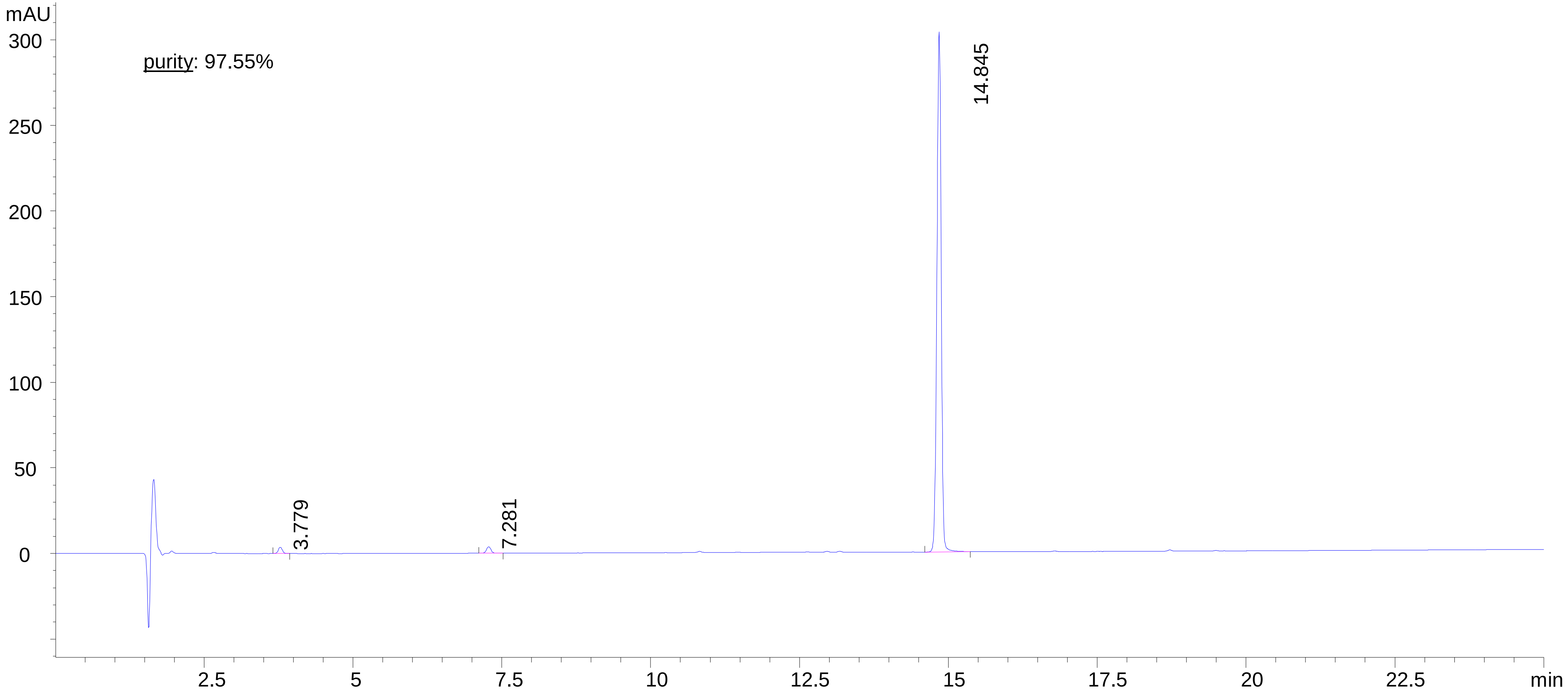

N-(6-bromobenzo[*d*]thiazol-2-yl) mycophenolate (**A13**):

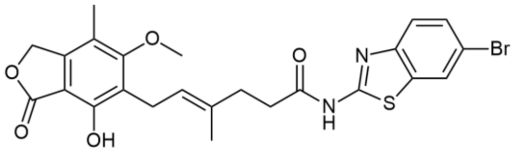

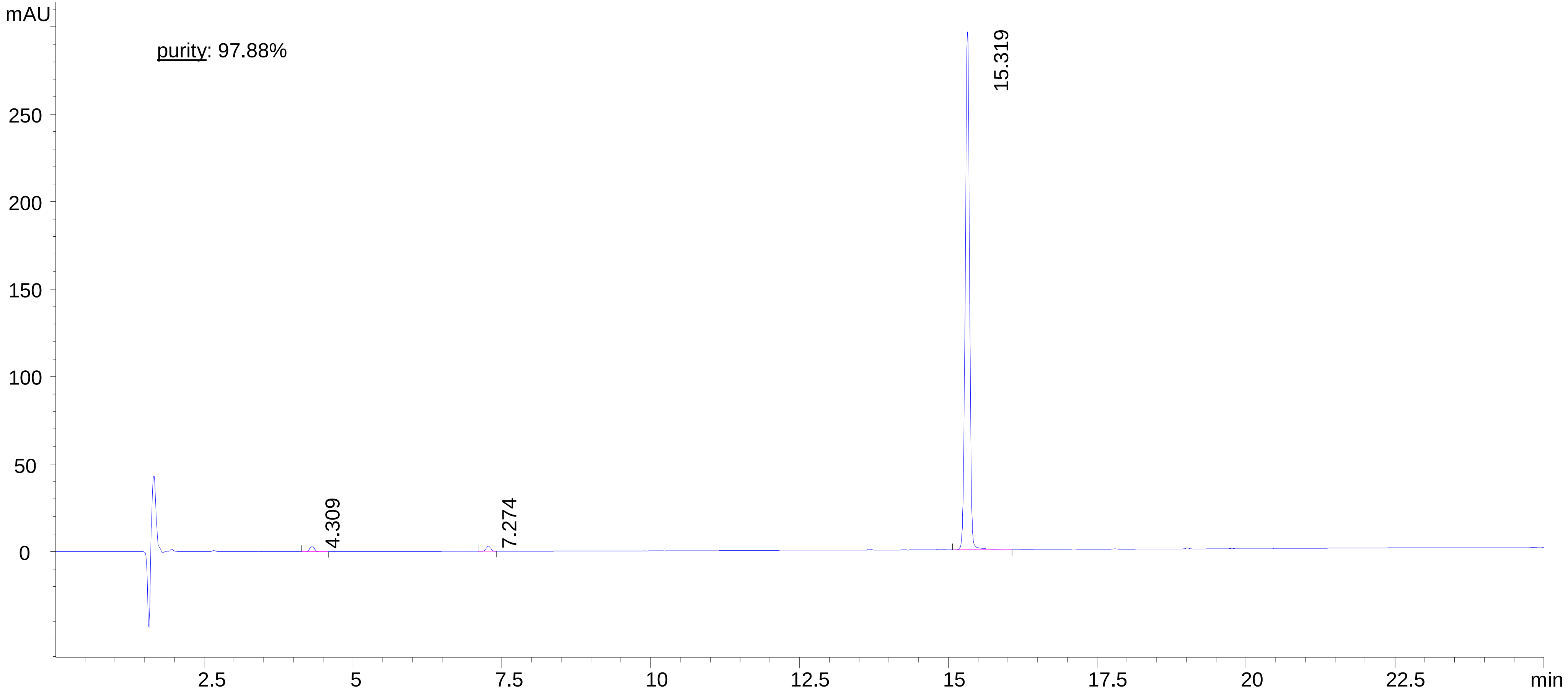

N-[6-(trifluoromethyl)benzo[*d*]thiazol-2-yl] mycophenolate (**A14**):

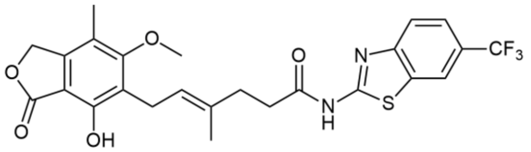

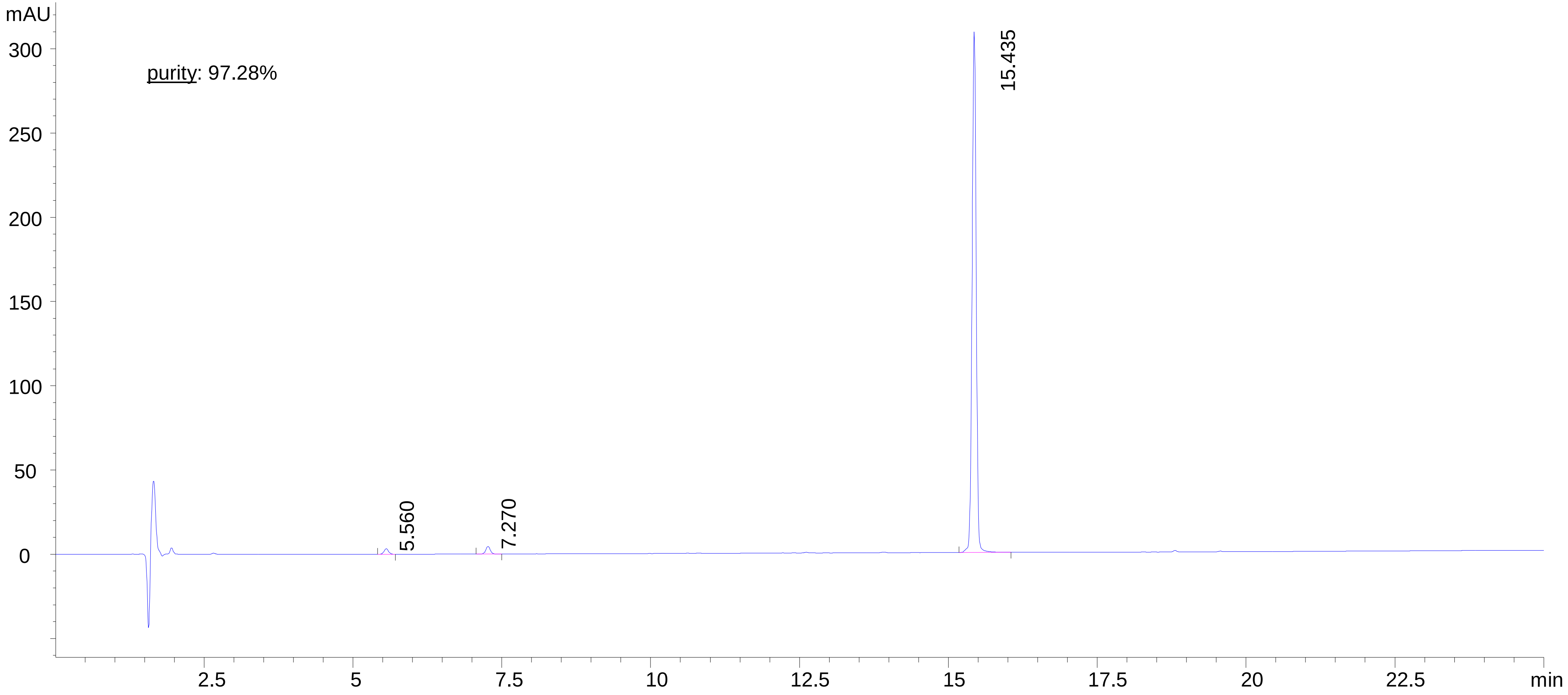

N-(6-nitrobenzo[*d*]thiazol-2-yl) mycophenolate (**A15**):

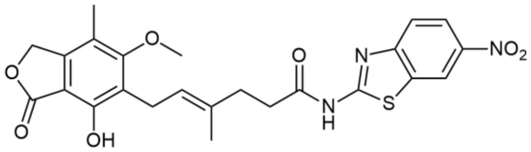

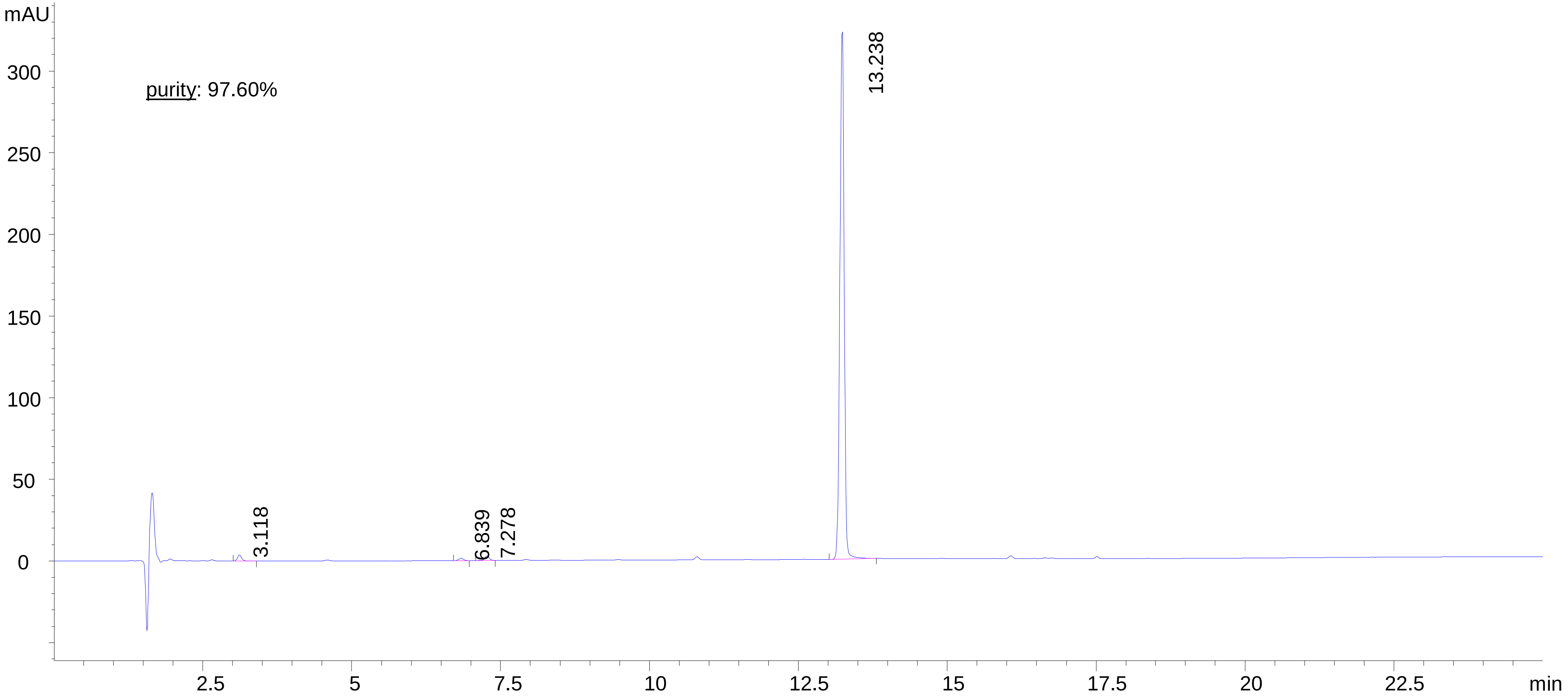

N-(4-methoxybenzo[*d*]thiazol-2-yl) mycophenolate (**A16**):

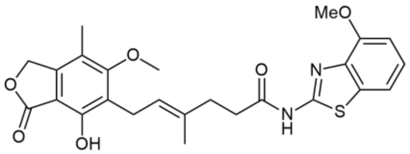

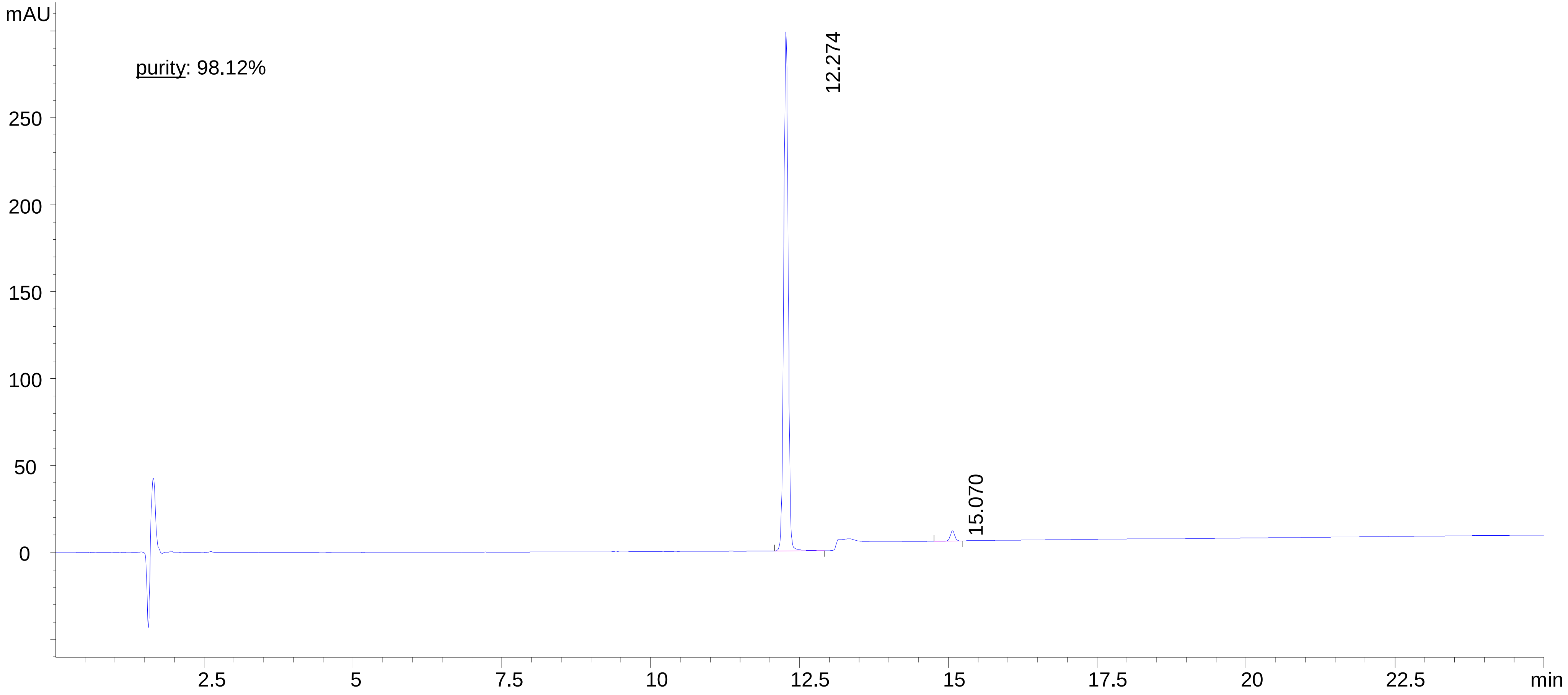

N-(4-methylbenzo[*d*]thiazol-2-yl) mycophenolate (**A17**):

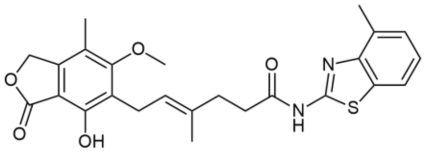

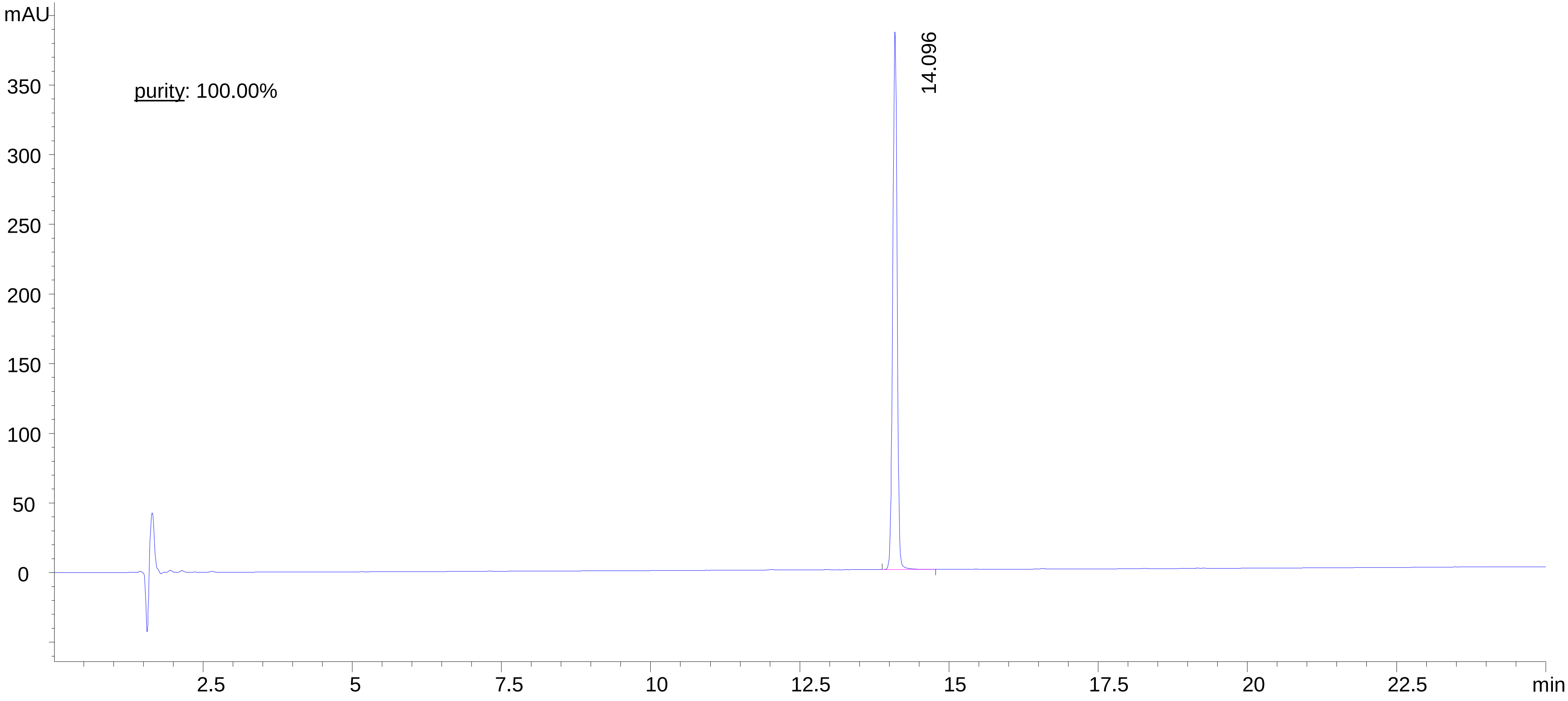

N-(4-chlorobenzo[*d*]thiazol-2-yl) mycophenolate (**A18**):

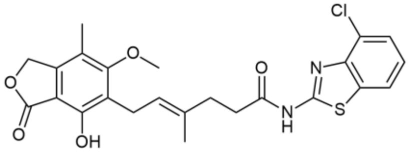

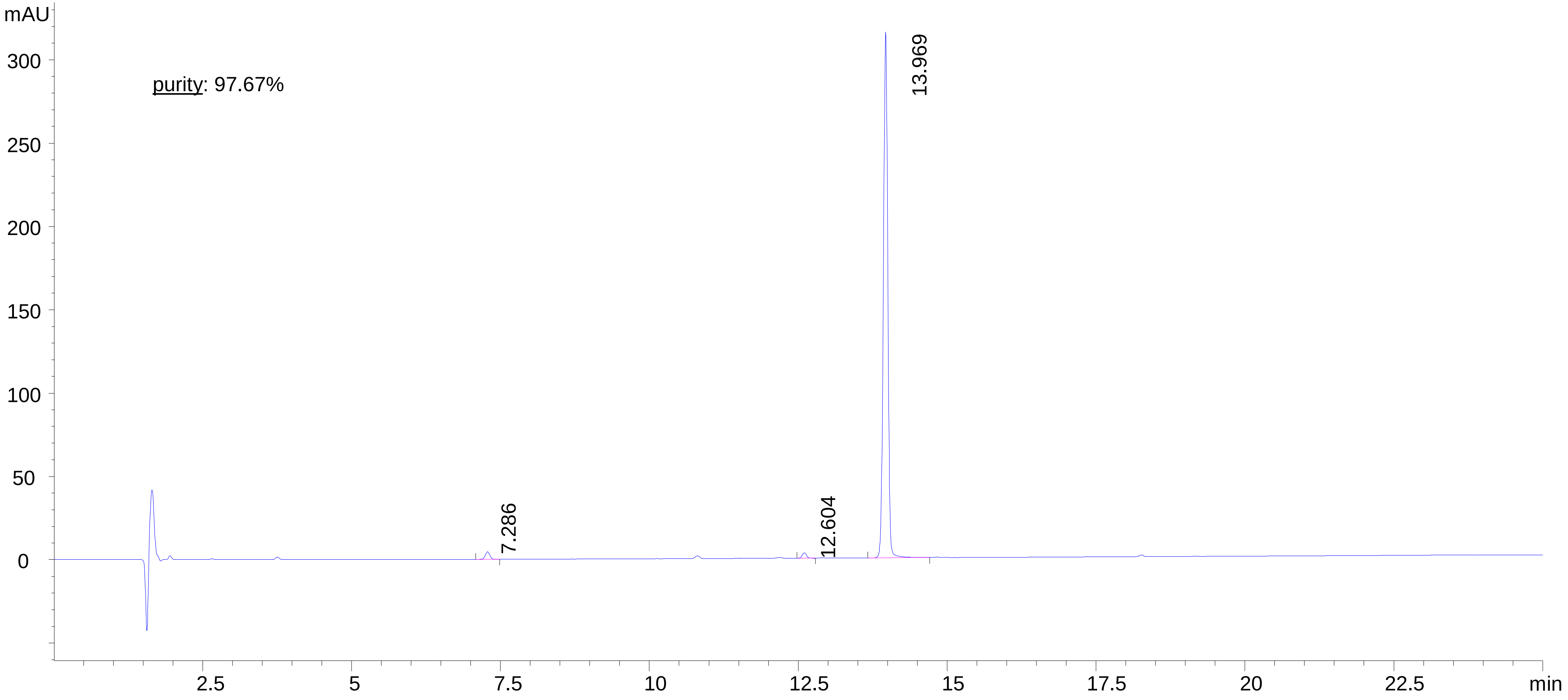

Mycophenolic acid (**MPA**):

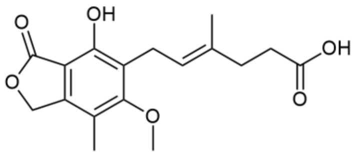

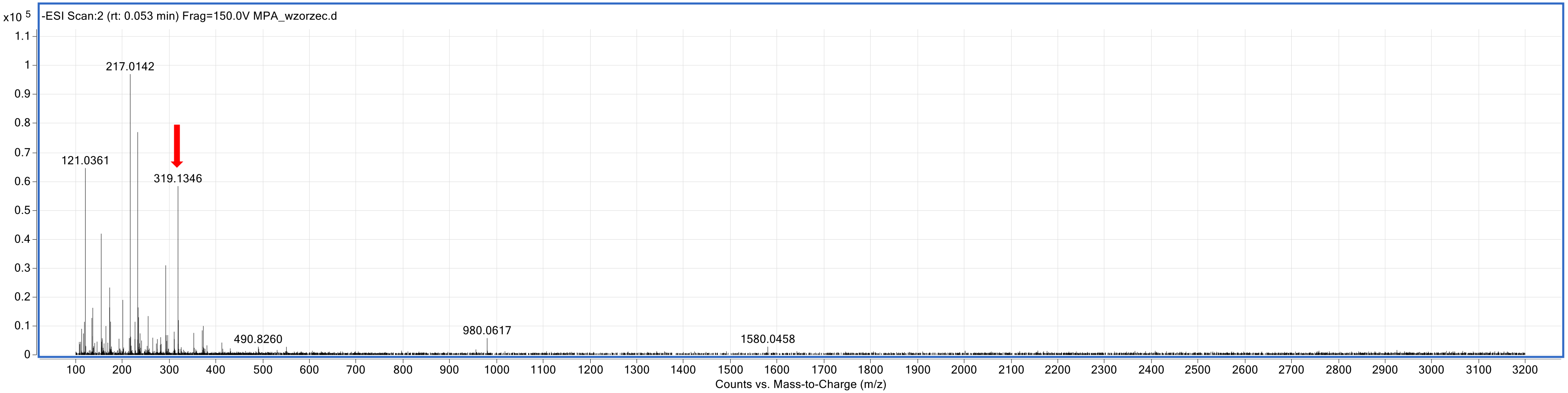

N-(benzo[*d*]thiazol-2-yl) mycophenolate (**A1**):

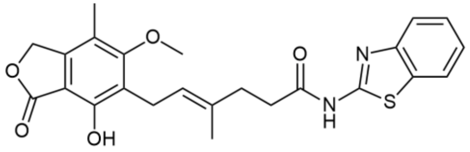

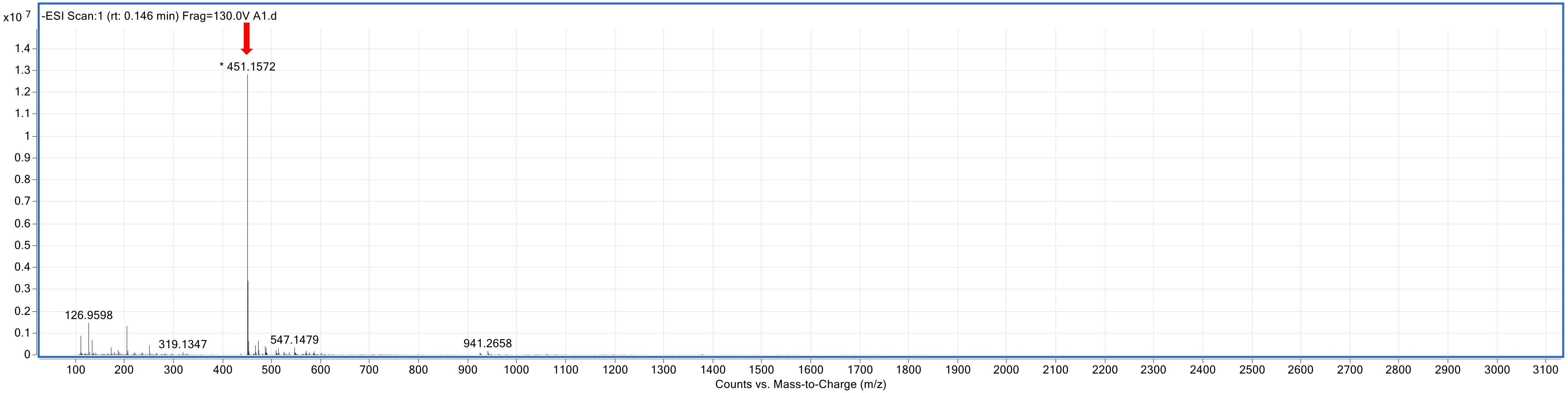

N-(benzo[*d*]oxazol-2-yl) mycophenolate (**A2**):

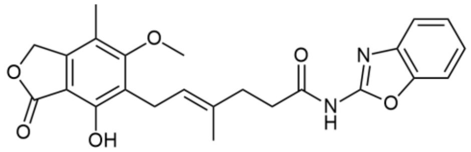

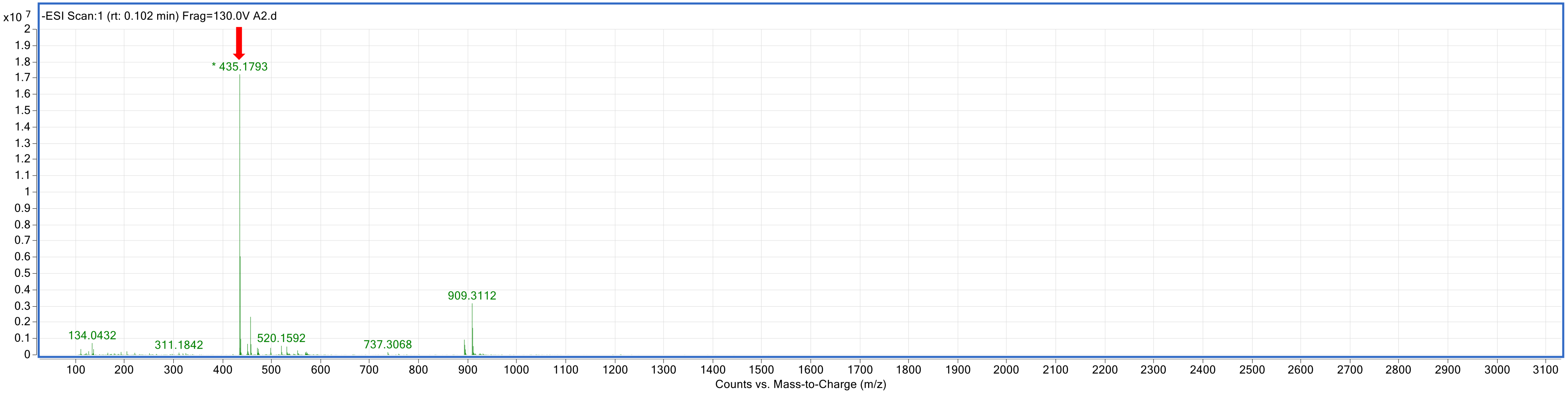

N-(1*H*-benzo[*d*]imidazol-2-yl) mycophenolate (**A3**):

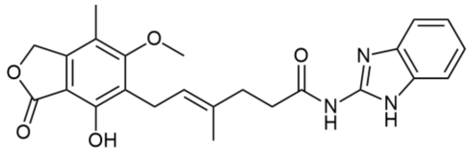

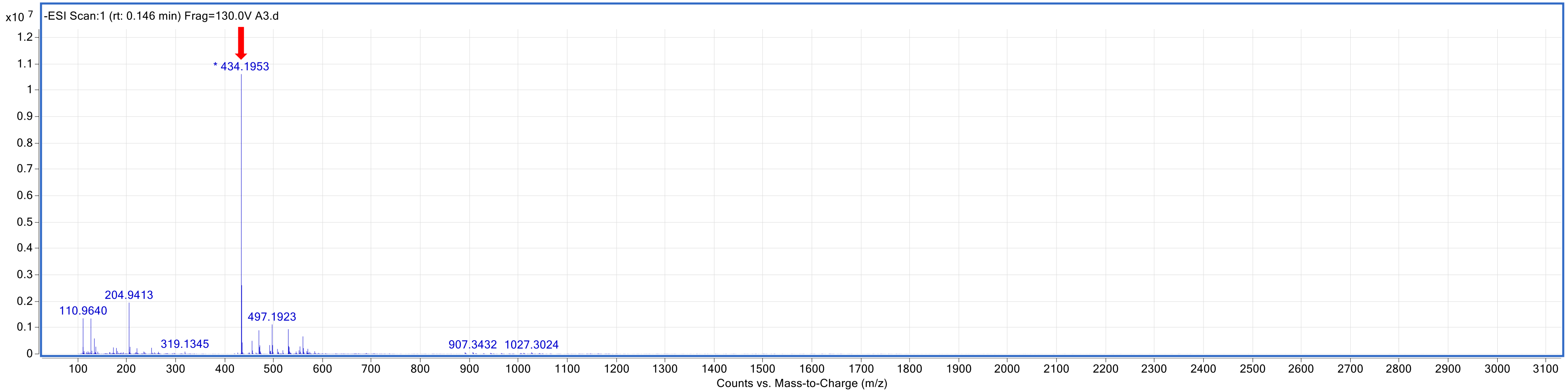

N-(benzo[*d*]thiazol-6-yl) mycophenolate (**A4**):

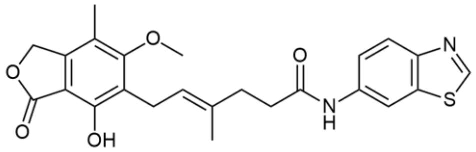

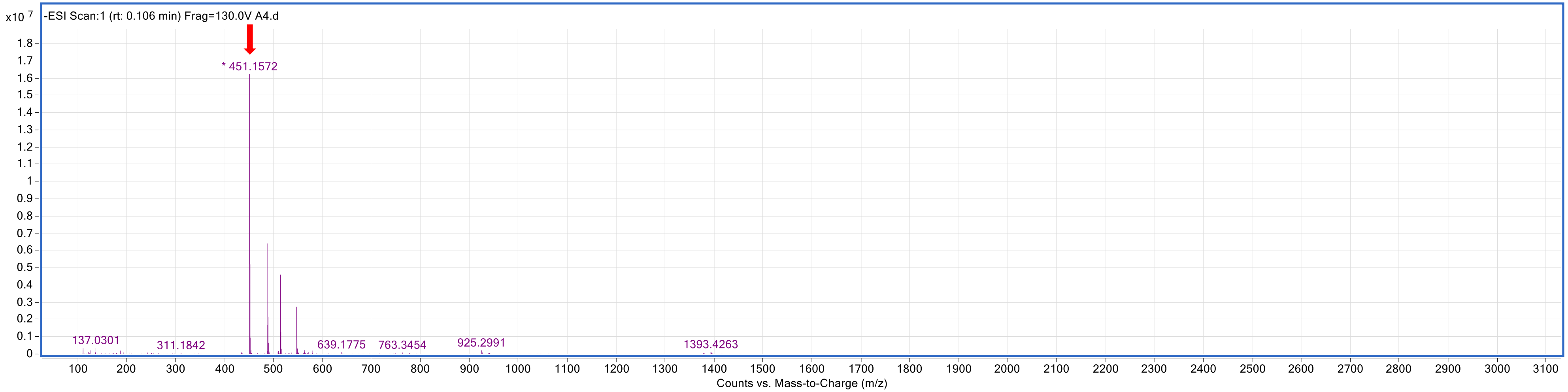

N-(benzo[*d*]thiazol-5-yl) mycophenolate (**A5**):

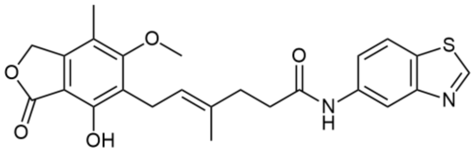

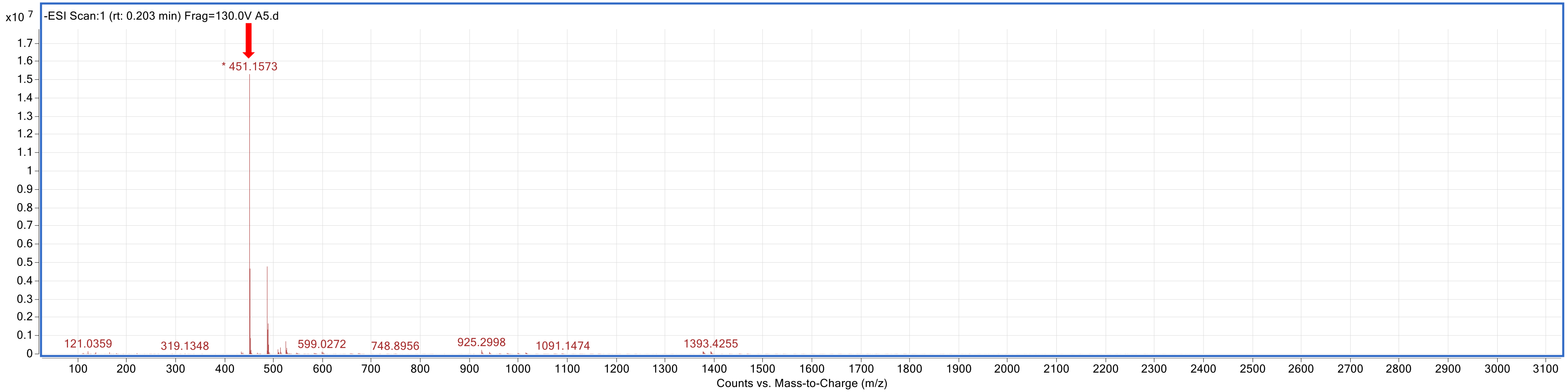

N-(benzo[*d*]oxazol-5-yl) mycophenolate (**A6**):

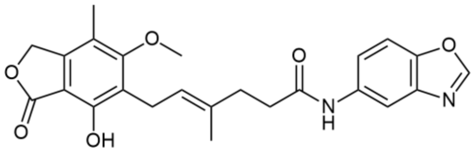

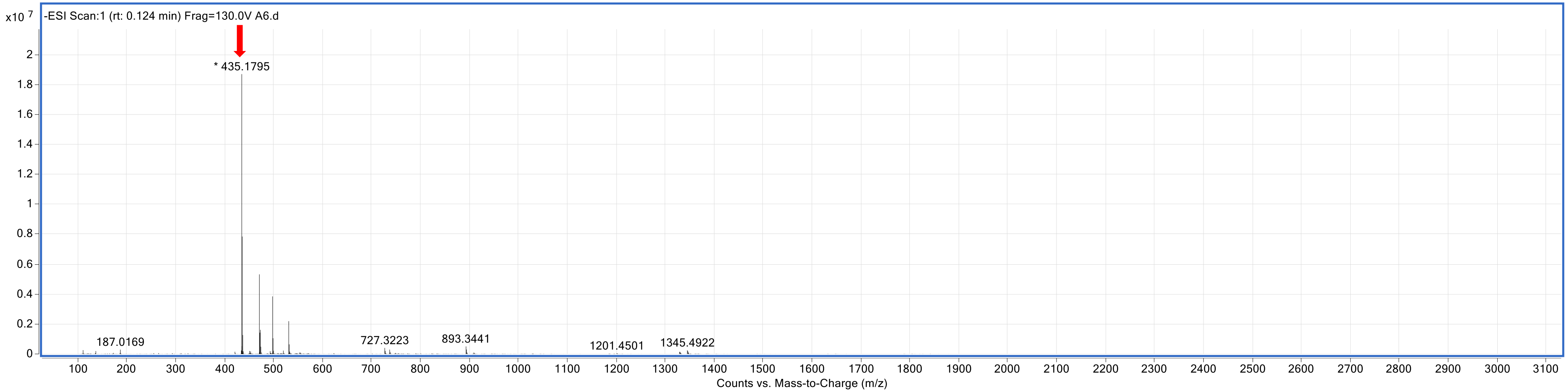

N-[1-(pirymidin-2-yl)methyl] mycophenolate (**A7**):

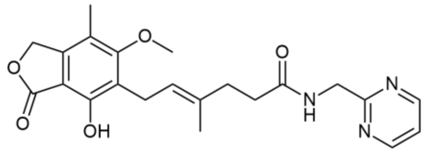

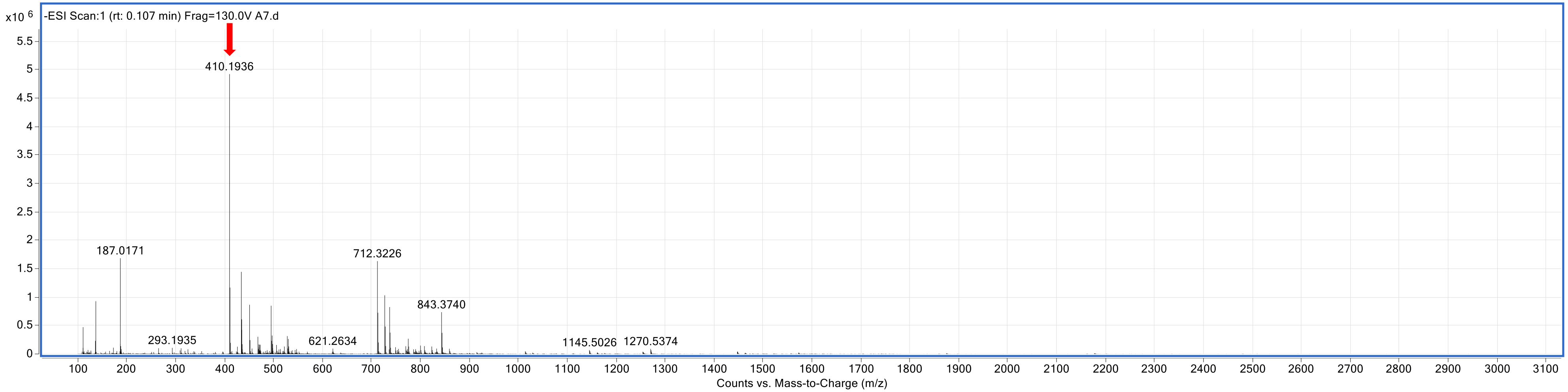

N-(6-methoxybenzo[*d*]thiazol-2-yl) mycophenolate (**A8**):

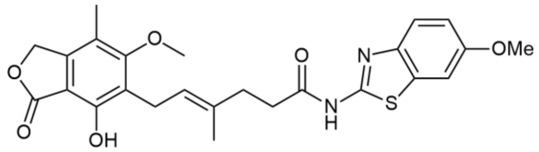

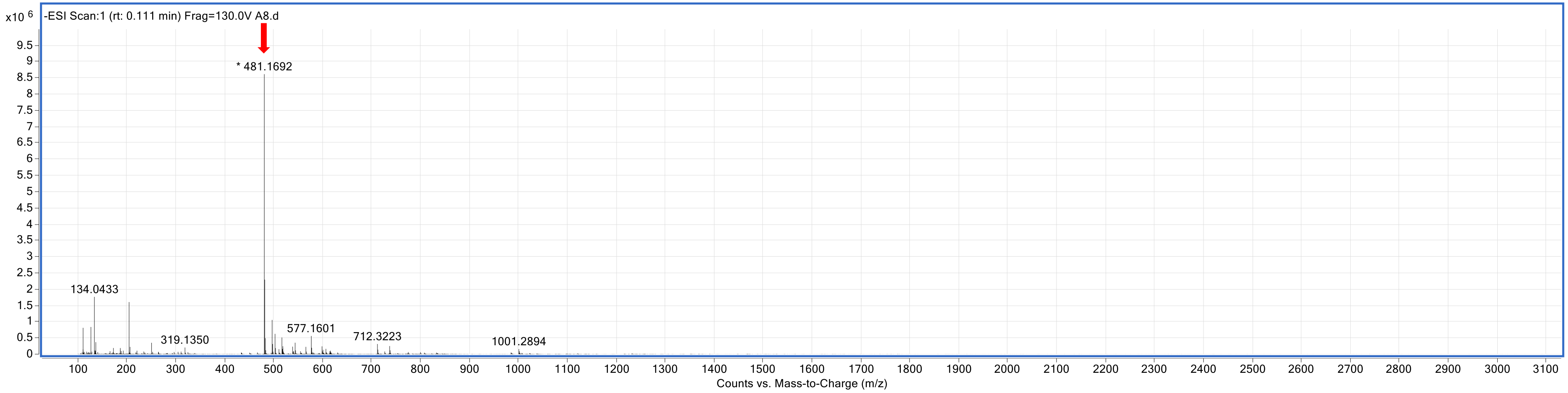

N-(6-methylbenzo[*d*]thiazol-2-yl) mycophenolate (**A9**):

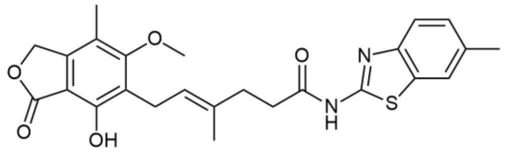

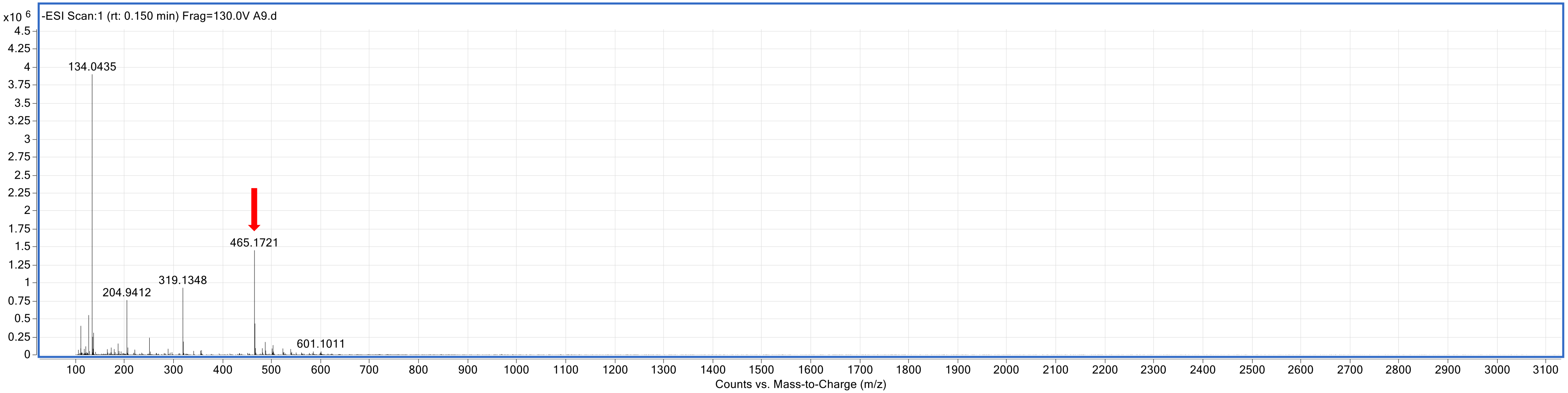

N-(5,6-dimethylbenzo[*d*]thiazol-2-yl) mycophenolate (**A10**):

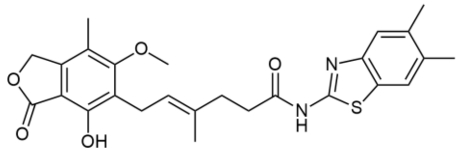

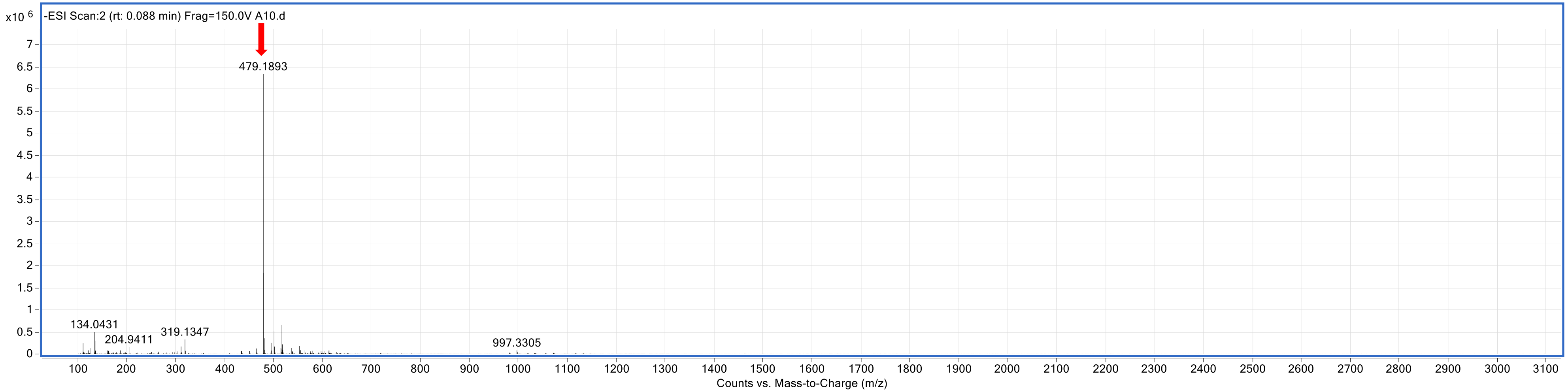

N-(6-fluorobenzo[*d*]thiazol-2-yl) mycophenolate (**A11**):

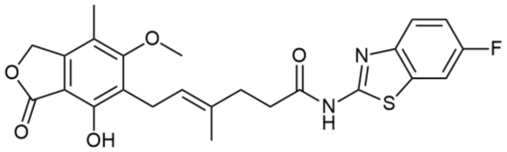

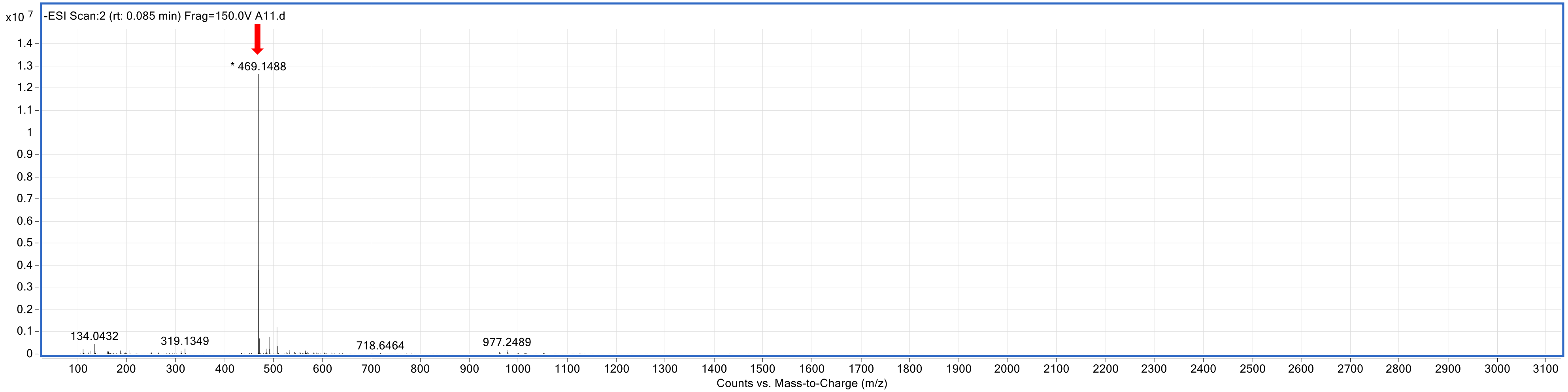

N-(6-chlorobenzo[*d*]thiazol-2-yl) mycophenolate (**A12**):

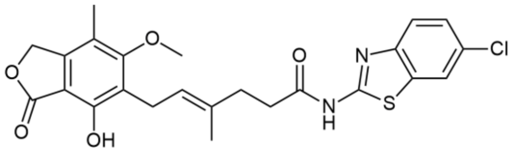

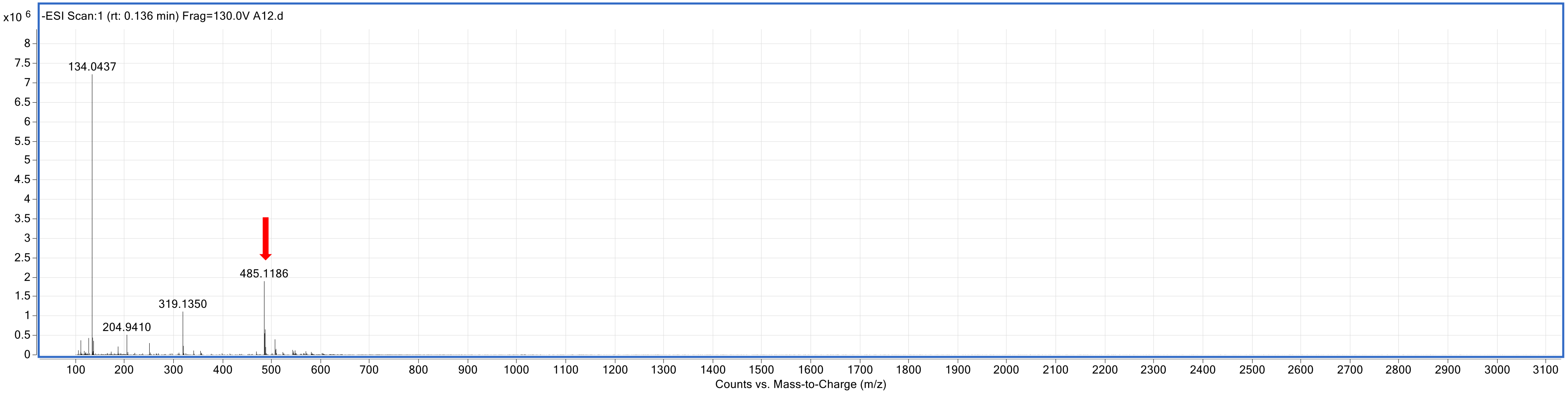

N-(6-bromobenzo[*d*]thiazol-2-yl) mycophenolate (**A13**):

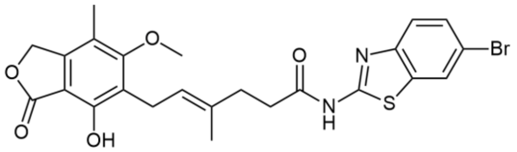

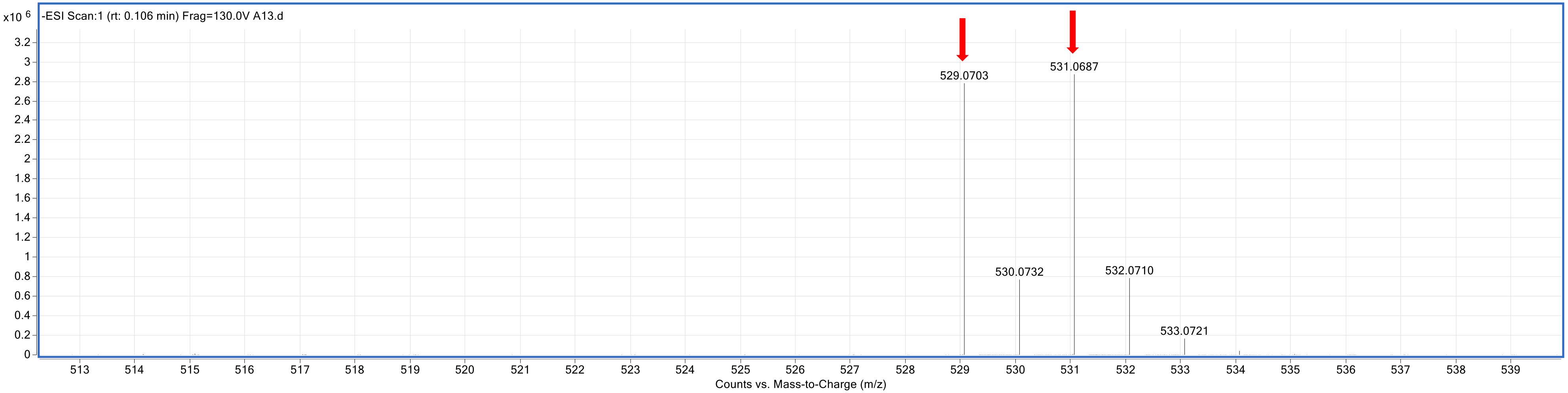

N-[6-(trifluoromethyl)benzo[*d*]thiazol-2-yl] mycophenolate (**A14**):

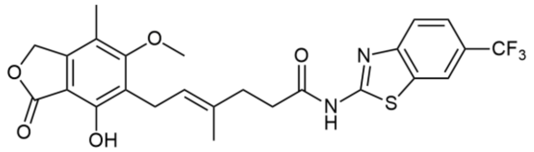

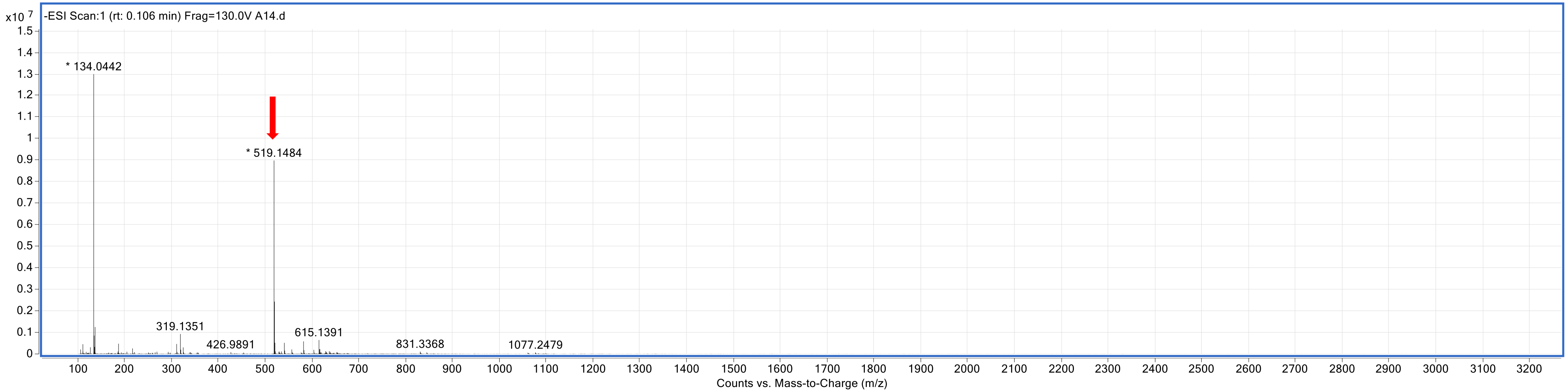

N-(6-nitrobenzo[*d*]thiazol-2-yl) mycophenolate (**A15**):

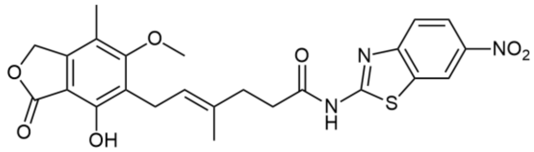

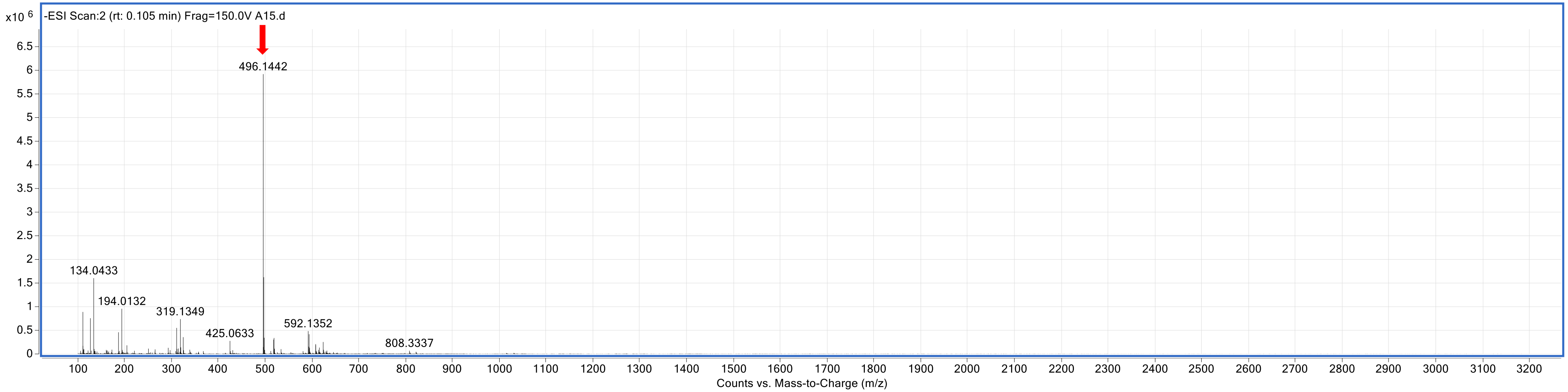

N-(4-methoxybenzo[*d*]thiazol-2-yl) mycophenolate (**A16**):

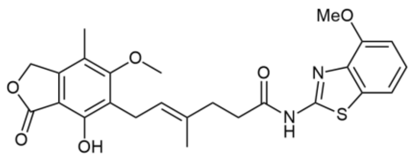

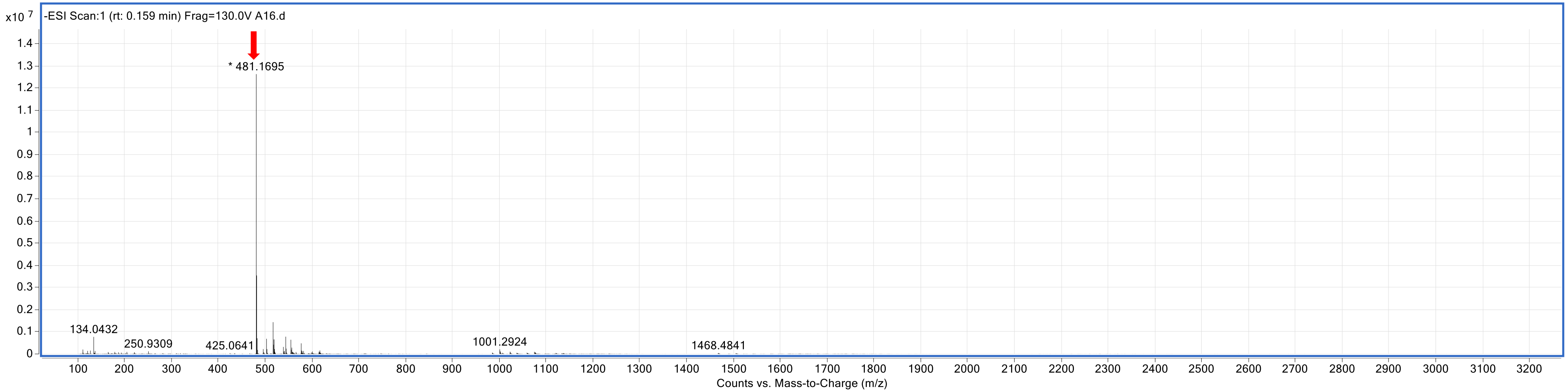

N-(4-methylbenzo[*d*]thiazol-2-yl) mycophenolate (**A17**):

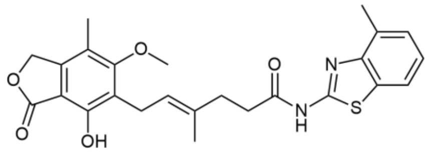

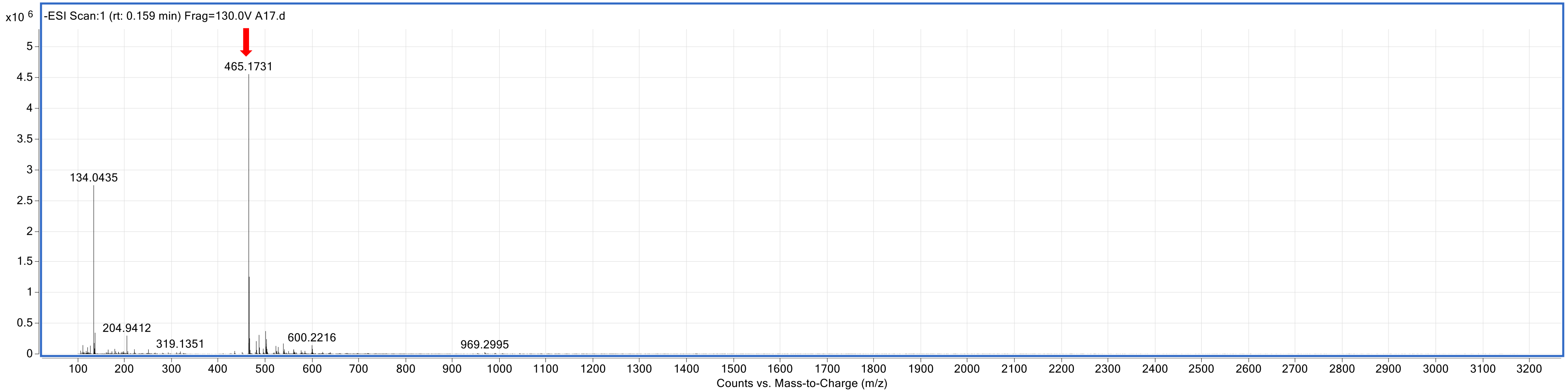

N-(4-chlorobenzo[*d*]thiazol-2-yl) mycophenolate (**A18**):

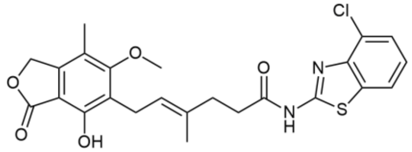

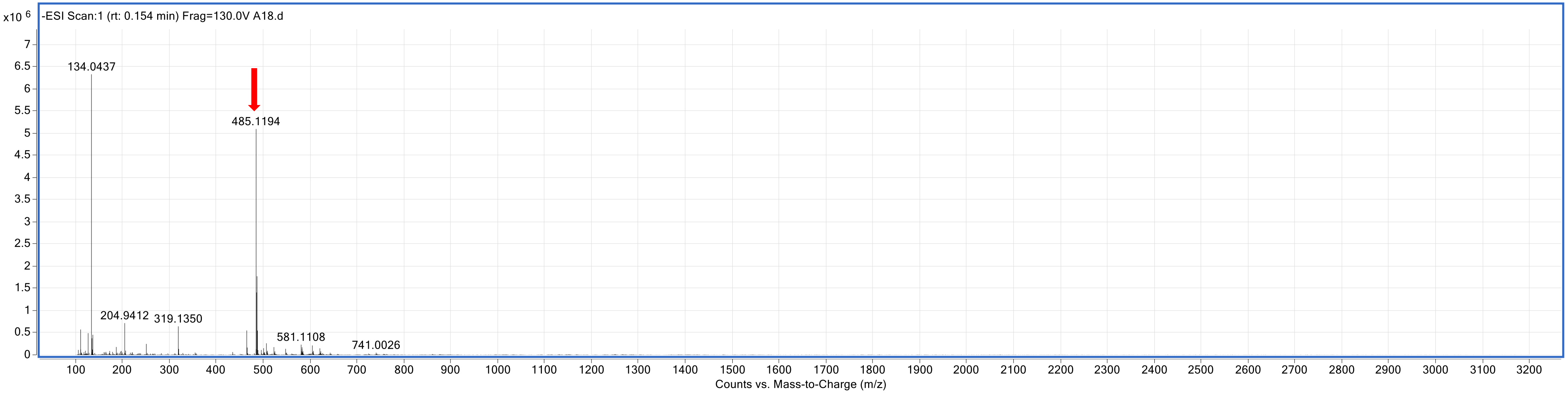

Brief study on melting points

Beside influencing reactions outcomes, the electron affinity of amine's substituent should affect amide's melting points. In general, 4-substituted 2-aminobenzo[*d*]thiazoles (**A16**-**A18**) are marked by lowered melting points in contrast to their 6-substituted counterparts (**A1**, **A8**-**A15**). Within this group one can observe melting point growth with molecular weight increase rather than altering electron character of substituent however correlation between these two parameters is not this simple. Slightly lower melting point values were observed in **A2** and **A4**-**A7** examples, probably due to different hydrogen bonding abilities arising from different electronegativity of heteroatoms and modified atom arrangement. Accumulation of strongly electronegative nitrogen atoms in **A3** had a great impact on hydrogen bonding and thus melting point of amide derivative. Some premises concerning structural units' contribution into the enthalpy of melting and hence melting point may be found in the literature[ESI1].

Beside strict analysis of real melting point values, calculations based on the literature[ESI1] have been made before proper assessment. They were made based on following equations:

$$\Delta H_m = \sum n_i m_i \tag{1}$$

$$\Phi = 2.435^{SP3+0.5SP2+0.5RING-1} \tag{2}$$

$$\Delta S_m = C - R\ln\sigma + R\ln\Phi \tag{3}$$

$$T_m = \frac{\Delta H_m}{\Delta S_m} - 273.15 \tag{4}$$

where:

ΔH_m — total phase change enthalpy of melting $\left[\frac{kJ}{mol}\right]$,

Φ — molecular flexibility number [—],

ΔS_m — total entropy of melting $\left[\frac{J}{K\cdot mol}\right]$,

T_m — melting point $[^0C]$,

n_i — number of times group i appears in compound [—],

m_i — contribution of group i to enthalpy of melting [—],

$SP3, SP2, RING$ — the number of non-ring, nonterminal sp^3/sp^2 atoms, the number of single or fused aromatic systems [—],

C — experimentally established constant $\left[\frac{J}{K\cdot mol}\right]$,

σ — the number of positions into which molecule can be rotated [—],

R — gas constant $\left[\frac{J}{K\cdot mol}\right]$.

As the first example a calculation for mycophenolic acid (**MPA**) is provided below.

$$SP3 = 4, SP2 = 3, RING = 1, C = 50, R = 8.31445\ \frac{J}{K\cdot mol}, \sigma = 1$$

$$\Delta H_m = COOH(X) + OH(Y) + COO(Y) + O(Y) + 2\cdot CH_3(Y) + CH_3(X) + CH_2(YY) + 3\cdot CH_2(Y) += C < (Y) + \\ = CH - (Y) + 6\cdot C_{Ar}$$

$$\Delta H_m = 9.487 + 6.086 + 7.391 + 4.118 + 2\cdot 2.688 + 1.183 + 2.834 + 3\cdot 2.365 - 4.207 + 2.147 - 6 * 0.561 \\ = 38.14\ \frac{kJ}{mol}$$

$$\Phi = 2.435^{4+0.5\cdot3+0.5\cdot1-1} = 85.60$$

$$\Delta S_m = 50 - 8.31446 \cdot \ln 1 + 8.31446 \cdot \ln 85.60 = 87.00 \frac{J}{K \cdot mol}$$

$$T_m = \frac{\Delta H_m}{\Delta S_m} - 273.15 = \frac{38.14 \cdot 1000}{87.00} - 273.15 = 165.3\ ^0C$$

As the second example a calculation for **A2** is provided below.

$$SP3 = 5, SP2 = 3, RING = 2, C = 50, R = 8.31445 \frac{J}{K \cdot mol}, \sigma = 1$$

$$\Delta H_m = \Delta H_m(MPA) - COOH(X) + CONH(Y) + 3 \cdot C_{Ar} + 4 \cdot CH_{Ar} + N_{Ar} + S_{Ar}$$

$$\Delta H_m = 38.14 - 9.487 + 10.947 - 3 \cdot 0.561 + 4 \cdot 1.642 + 2.58 + 2.915 = 50.01 \frac{kJ}{mol}$$

$$\Phi = 2.435^{5+0.5 \cdot 3+0.5 \cdot 2-1} = 325.27$$

$$\Delta S_m = 50 - 8.31446 \cdot \ln 1 + 8.31446 \cdot \ln 325.27 = 98.10 \frac{J}{K \cdot mol}$$

$$T_m = \frac{\Delta H_m}{\Delta S_m} - 273.15 = \frac{50.01 \cdot 1000}{98.10} - 273.15 = 236.7^0C$$

Table 1 summarizes the calculated melting points (**MP$_{cal.}$**) and measured ones (**MP$_{exp.}$**), disjunction between them (**Δ**) as well as absolute relative error (**δ**) devised for average value of actual melting point measurement.

Symbol	MP$_{cal.}$ [^{o}C]	MP$_{exp.}$ [^{o}C]		Δ [^{o}C]	δ [%]
		min	max		
**MPA**	165.3	142.8	144.9	21.5	14.9
**A1**	236.7	204.5	210.0	29.5	14.2
**A2**	231.4	174.5	177.0	55.7	31.7
**A3**	295.0	253.0	254.5	41.3	16.3
**A4**	236.7	175.0	176.3	61.1	34.8
**A5**	236.7	114.4	116.7	121.2	104.8
**A6**	231.4	83.8	87.2	145.9	170.6
**A7**	230.5	106.9	109.6	122.3	112.9
**A8**	235.6	246.1	247.9	-11.4	4.6
**A9**	247.3	256.8	259.7	-11.0	4.2
**A10**	258.0	245.6	247.2	11.6	4.7
**A11**	241.7	232.1	235.6	7.8	3.4
**A12**	253.4	261.4	263.3	-9.0	3.4
**A13**	258.2	264.6	267.4	-7.8	2.9
**A14**	206.7	261.2	263.5	-55.7	21.2
**A15**	250.1	278.0	280.0	-28.9	10.4
**A16**	235.6	165.4	167.2	69.3	41.7
**A17**	247.3	173.3	175.8	72.8	41.7
**A18**	253.4	223.2	226.7	28.5	12.6

Table 1. Melting point evaluation of new amide derivatives **A1**-**A18** and **MPA** as well

Despite the tentative character of these calculations, some conclusions may be drawn. In general, relative error of these estimations is within the range between 2.9 and 170.6%. The biggest over-estimation can be seen in the case of 6- and 5-substituted benzothiazole and 5-substituted benzoxazole as well as pyrimidine derivative, namely **A4**-**A7**, as this method does not differentiate loci influence on enthalpy of melting, and thus on melting point, and under-records heteroatom involvement.

The remaining largest underestimations are observed for 2-aminobenzoxazole (**A2**) and 4-substituted 2-aminobenzothiazoles (**A16**-**A18**). Heteroaromatic oxygen contribution into enthalpy of melting seems to be the most over-rated among the rest of heteroaromatic fragments. 10 of 18 amides have the estimation error within absolute error value, which was determined to be 33.2 degree for this method [1]. 9 over 18 derivatives possess negative disjunction value (however for one example it almost equals zero value) suggesting lack of specific interactions reckoning within this approach. Remaining examples are represented by the opposite sign thus exhibiting over-estimation of present interplay.

Aforementioned deliberations show ineffectiveness of this model for such multifunctional derivatives, implicitly heterocyclic, with vague hydrogen bonding architecture and other intermolecular interactions. Despite these facts, one may assume that the disjunction symbol may be convergent with the ease of hydrogen bonds formation (as well as their distribution), while its value reflects the strength of this effect. Eventually, these factors, to some extent, affect the final melting point value.

ESI1. Jain A, Yang G, Yalkowsky SH. Estimation of Melting Points of Organic Compounds. Ind Eng Chem Res 2004;43:7618–7621.

PBMCs proliferation measurements

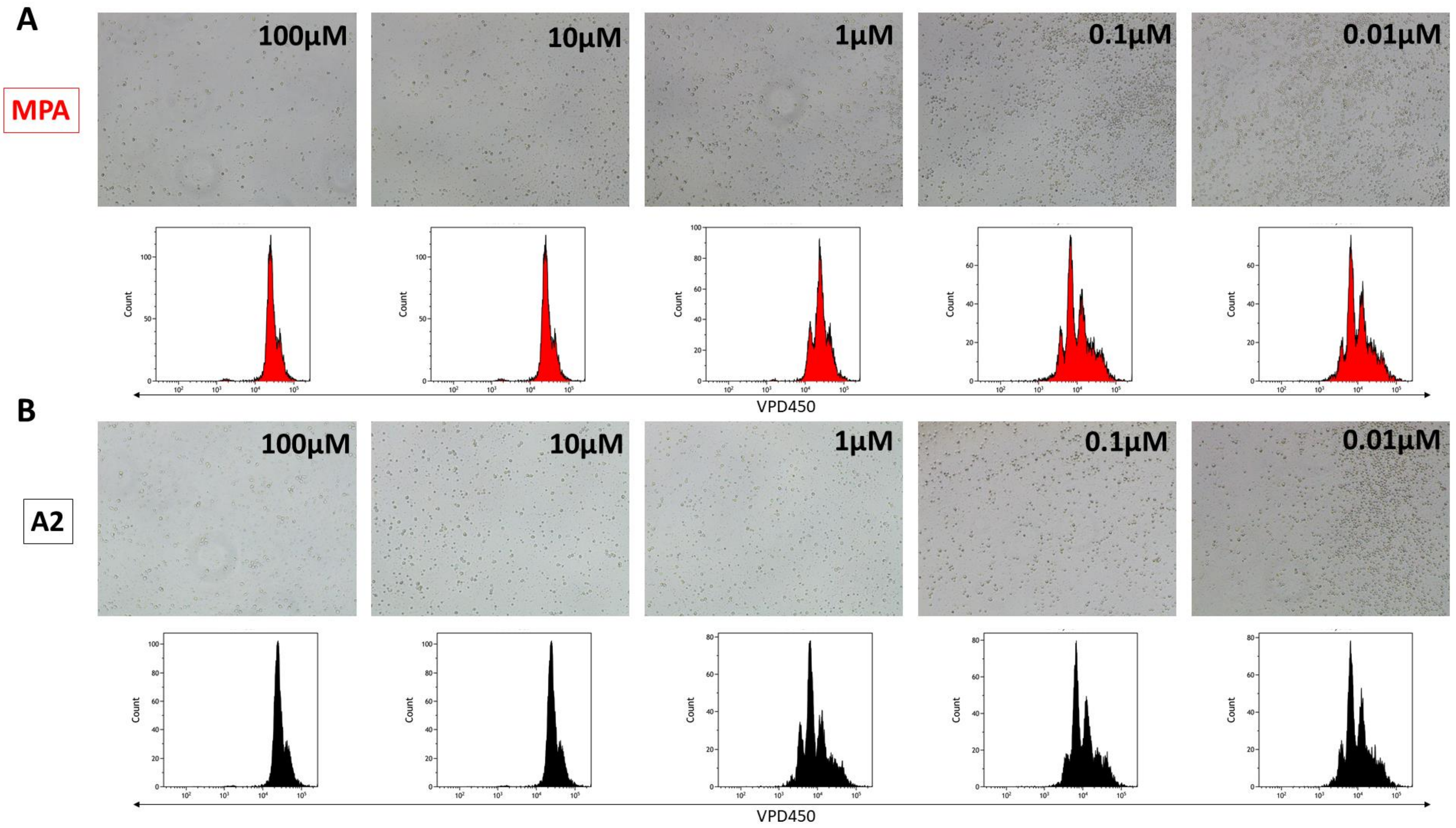

ESI Figure 1. PBMCs proliferative response. Detection of cell proliferation in PBMCs by VPD450 staining assay of cells treated with different concentrations (100; 10; 1; 0.1 and 0.01 µM) of **MPA** (A, red) and A2 (B, black) for 96 h by flow cytometry. The effect of **MPA** and **A2** on PBMCs observed using an inverted microscope. Data presents analysis of one representative experiment with the most active compound **A2** and **MPA** as a control

A7 and A18 as IMPDH inhibitors — mechanistic approach

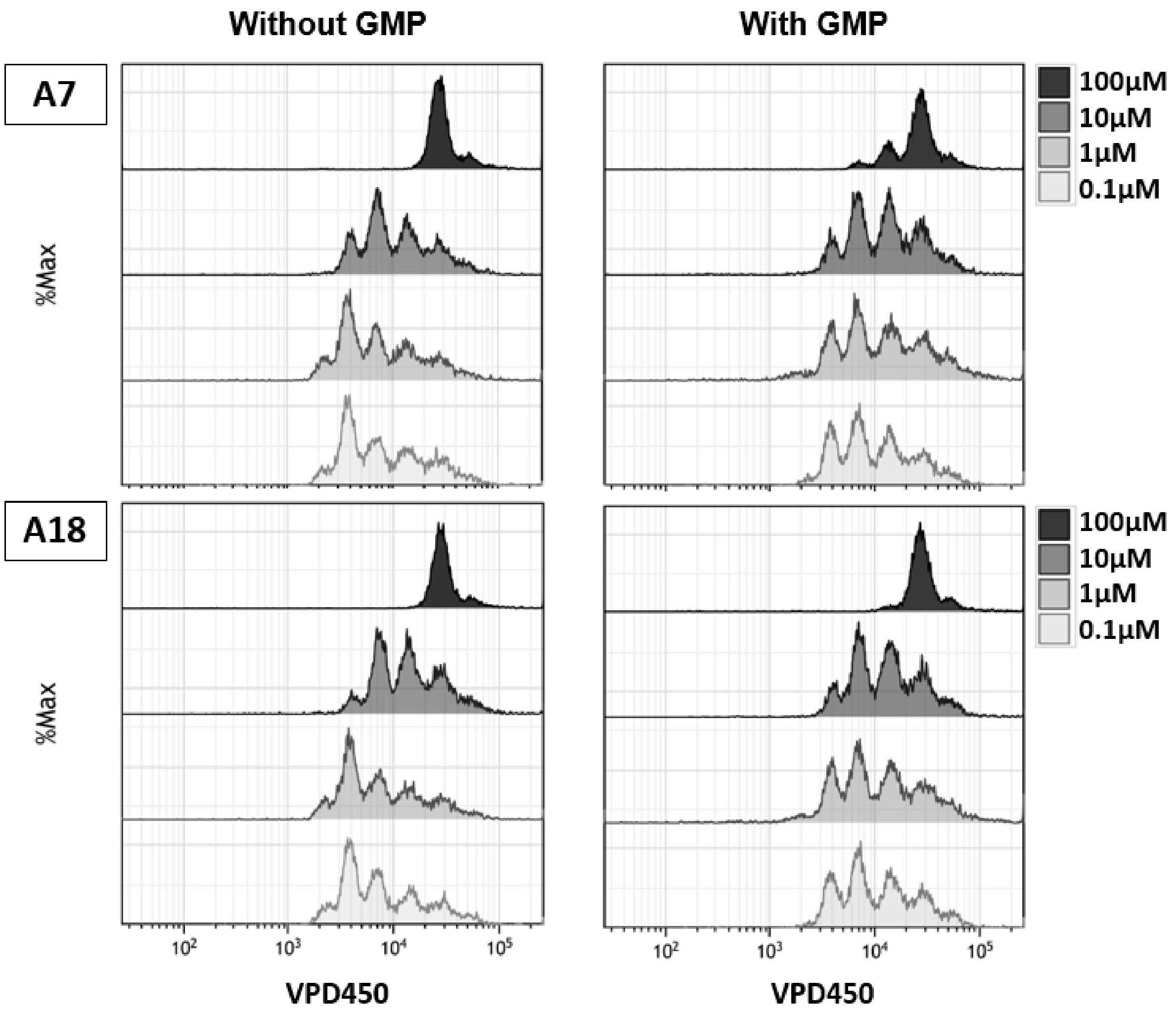

ESI Figure 2. Representative antiproliferative activity of **A7** and **A18**. VPD 450-labeled human PBMCs, in the presence of different concentrations (100; 10; 1 and 0.1 µM) **A7 or A18** and stimulated (magnetic beads coated with anti-CD3 and anti-CD28 antibodies) were cultured with or without the addition of 50 µM GMP for 72 h. Cells proliferation was analyzed using flow cytometry.
